# Supplementary material for: Application of the theory of regulatory fit to promote adherence to evidence-based breast cancer screening recommendations: experimental versus longitudinal evidence
Source: BMJ Open. 2020 Nov 12;10(11):e037748. doi: 10.1136/bmjopen-2020-037748 (PMC7662420; doi:10.1136/bmjopen-2020-037748)
Supplement: Supplementary data [file bmjopen-2020-037748supp001.pdf]

SUPPLEMENTAL MATERIAL

An Application of the Theory of Regulatory Fit to Promote Adherence to Evidence-Based Breast Cancer Screening Recommendations Reducing Unnecessary Breast Cancer Mammography in Women Below Under 50: Experimental vs. Longitudinal Evidence

Serena Petrocchi, Ramona Ludolph, Nanon H. Labrie, Peter J. Schulz

Correspondence to:

Dr. Serena Petrocchi, Institute of Communication and Health, Università della Svizzera italiana, Via Buffi 13, 6900 Lugano, Switzerland.

E-mail: serena.petrocchi@usi.ch

Phone: 0041 (0) 58 666 4821

Study 1

Pre-test measures

Health Status and Healthy Lifestyle. Questions measured overall health status as perceived by the participants on a 5-point Likert scale and healthy lifestyle behaviours (i.e., diet, physical activity, smoking habits, alcohol consumption; see,[1].

Breast Cancer/Mammography Experience and Knowledge of the Ticino screening program. Participants replied to a set of questions on: past diagnosis of breast cancer among first-grade relatives,[2], if they had a mammography in the past, if doctor recommended the mammography, if they had a breast biopsy, if they know the breast cancer screening program in Ticino, and its age thresholds.

Fear of Breast Cancer. Four of the original 8 items of the Fear of Breast Cancer scale,[3,4] were administered. Items asked participants to rate their emotional reaction about breast cancer saying how much they agreed with the statements ‘When I think about breast cancer, I feel nervous (or: I get upset, I get jittery, I feel anxious)’. Participants replied on a 5-point Likert scale from 1 (strongly disagree) to 5 (strongly agree). Data from the present sample show that internal consistency was good, Cronbach’s $\alpha = .88$, rs > .73, as well as the factor structure, χ^2 (1) = 2.04, p = .15, CFI = .99, RMSEA = .05.

Ego-involvement. The Personal Involvement Inventory,[5] were administered measuring participants’ involvement in breast cancer screening through affective and cognitive adjectives because previous research,[4]. The scale was administered as a 7-digit semantic differential (e.g., important/unimportant, relevant/irrelevant or worthless/valuable). The original item ‘of concern to me/of not concern to me’ was deleted based on results of a previous study,[4]. Data from the present sample show that internal consistency was good, Cronbach’s $\alpha = .91$, rs > .71, as well as and the factor structure, χ^2 (5) = 11.34, p = .04, CFI = .99, RMSEA = .06.

Perceived benefits of mammography screening. The perceived benefit of mammography screening was measured by four items,[6]: ‘Having a mammogram will help me find breast lumps

early'; 'If I find a lump early through a mammogram, my treatment for breast cancer may not be as bad'; 'Having a mammogram is the best way for me to find a very small breast lump'; 'Having a mammogram will decrease my chance of dying from breast cancer'. Participants replied on a 5-point Likert-scale from 1 (strongly disagree) to 5 (strongly agree). Data from the present sample show that internal consistency was modest, Cronbach's $\alpha = .75$, rs > .49 and the factor structure was good, χ^2 (1) = .51, p = .47, CFI = 1, RMSEA = .00.

Experimental manipulation

Regulatory Focus Priming Procedure. Prevention induced participants were asked to list two of their current obligations and then write down five actions they could take to avoid failure in fulfilling them,[7]. Promotion induced participants were asked to list two aspirations and write down five actions they could take to ensure their accomplishment,[7].

Video Messages. Participants in the promotion fit condition watched a video message emphasizing promotion concerns (i.e., they should adhere to evidence-based recommendations on mammography screening for safety and health protection reasons). Participants in the prevention fit condition watched a video emphasizing prevention concerns (i.e., they should not abstain from following the evidence-based recommendations on mammography screening to avoid negative/side effects). Participants in the control group did not receive any priming and read a general health leaflet. See Supplemental Table 1 for details of the voice-text of the two video messages and the control leaflet.

Supplemental Table 1. Voice-text of the video messages and control leaflet of the Study 1.

STUDY 1

Promotion video-message	Prevention video-message
The mammography screening is a method for the early detection of breast cancer. Using x-rays, mammograms can identify very small tumours generally longer before they are palpable. Mammograms is a method that is used early, often even without symptoms. In a screening program, experts recommend mammography from the age of 50. Here in Ticino, women aged 50 and over are invited to voluntarily undergo mammography every two years at one of the accredited Radiology Centres. For most women between the ages of 50 and 69, the benefits of screening are greater than the risks, but nevertheless it is important to be properly informed to make the best decision about mammography.	
**To protect their health, women under the age of 50, without a medical indication or family history of cancer, are excluded from the program.**	**To avoid adverse effects on their health, women under the age of 50 without a medical indication or family history of cancer are excluded from the program.**
Now I would like to explain the scientific reasoning behind the recommendation to not undergo a mammogram without a medical reason. Anyway, in case of doubt or symptoms, I suggest to contact your doctor. So, you are asking why women under the age of 50 are excluded from mammography screening. Scientific research shows that for women between the ages of 50 and 69 mammography screening is the most effective method for the early detection of breast cancer and for reducing the mortality rate associated with it. In contrast, for young women between the ages of 30 and 49, the disadvantages and risks to health are greater than the benefits. This is mainly due to the fact that women before menopause have a denser breast tissue.	
**Given the reasons I have just presented, one should avoid undergoing a mammogram early to prevent negative consequences.**	**Given the reasons I have just presented, to early undergo a mammogram can lead to negative consequences.**
For example, mammography could show anomalies that, after additional diagnostic tests, could be proved to be benign. This type of error is called a false positive. If for women aged between 50 and 69 this risk is minimal, for young women is higher due to the denser breast tissue. In addition, breast cancer could not be seen by mammograms because it is too small and therefore the exam may appear normal although cancer is present. This is a false-negative result. These risks always exist, but they are higher for young women. As all medical testing, waiting for the outcome of the mammogram can generate a state of anxiety and the procedure sometimes can be perceived as painful. Radiation exposure also have health consequences. Although the exposure is minimal, for women under the age of 50 the risk is higher than the benefits of mammography. Furthermore, screening could lead to over-treatment for tumours that are benign. Over diagnosis represents approximately 1-10% of diagnosed cancers. This would expose young women to the negative effects of anti-cancer therapies, without a real need. In the absence of scientific evidence of the effectiveness of mammographic screening for young women, the inclusion of young women in the program would entail additional costs for society. These financial resources could be used to prevent other diseases.	
**For these reasons, it is recommended that young women follow the indications for breast cancer screening.**	**For these reasons, it is recommended that young women do not ignore the indications related to breast cancer screening.**

Control Leaflet

Healthy eating associated with an active lifestyle is a useful way for disease prevention. An adequate and balanced diet plan guarantees an optimal supply of nutrients to meet the needs of your body. A balanced diet also allows to receive substances that play a protective and/or preventive role against diseases.
This booklet - thought for people of all ages without any particular diseases - explains the scientific reasoning behind the recommendation to follow a healthy diet even in the absence of particular weight or health disorders. Anyway, in case of doubts or problems, we suggest to contact your doctor.

'Man is what he eats.'

We eat several times a day, for a lifetime. With a life expectancy around 80, this corresponds to approximately 85.000 meals. Considering that each meal lasts an average of thirty minutes, we spend at least five years at a table. Adding the meal preparation time, the count easily rises to ten years or more. Nutrition is therefore a topic that deserves special attention. Balanced meals and a healthy lifestyle give a fundamental contribution to our daily well-being and efficiency.

«Fast casual» instead of «fast food»

'Fast casual' means eating healthy and fast and it is not a contradiction. The 'snack culture' is an old idea. Those who want to have a quick meal, however, must pay attention to its composition: dairy products, fruit, vegetables, salads and whole meal products are the basis for a new, healthy 'fast casual' menu.

The right fats for every need

The choice of fats must be made according to the intended use. Fats and oils, if heated for a long time, change with chemical reactions that can create unwanted substances harmful to health. In order to avoid such reactions, it is advisable to cook with fats that mainly contain saturated fatty acids, which are more stable at high temperatures.

Eating healthy is easy

Food provides the body with both the necessary energy and nutrients that allow it to function properly. The diet must therefore provide a correct caloric intake and a sufficient amount of nutrients. No food is so complete that it contains everything the body needs. The basic rule is therefore to eat everything and in a varied way.

Food pyramid

The food pyramid facilitates the quantitative choice between the various food groups. It is a useful tool for all those who want to have a healthy diet. It provides clear indications on what to eat, how often, and in what quantity.

How do I interpret it?

A balanced diet requires the foods at the base of the pyramid be consumed in greater quantities. Climbing up to the vertex, the quantities of food consumed should be limited. Nothing is forbidden, every food finds its place in a balanced diet, but the recommended quantities will depend on its location in the pyramid.

Give food the importance that deserves, eating healthier. Your health and well-being will be better!

Note: grey rounded rectangles show the common parts of promotion and prevention video-messages; orange rounded rectangles show the promotion video-message specific parts (text in bold); blue rounded rectangles show the prevention video-message specific parts (text in bold); the green rounded rectangle shows the content of the control leaflet. The Videos created for Study 1 can be retrieved from https://youtu.be/mperSG5_9yQ and https://youtu.be/KnhRUnDoSV0. Both videos last 3:28 minutes. The videos created for Study 2 can be retrieved from https://youtu.be/btM3HrvYDlQ, https://youtu.be/BZPjFPUQuvw, https://youtu.be/-lXzGpcmzD4, https://youtu.be/jRi8Y-sZvSc. A translation of the Italian voice-over has been provided in this Table.

Post-test Measures

Intention to ask for breast cancer screening. Intention was measured by the question "I am evaluating the idea to have a mammography screening for breast cancer in the next 2-3 years",[8]. Two further questions were added: "I have the intention to have a mammography screening for breast cancer in the next 2-3 years" and "I will take an appointment for a mammography screening for breast cancer in the next 2-3 years". Participants replied on a 5-point scale from 1 (definitely yes) to 5 (definitely not); participants' scores ranged 1-5, M = 2.61, and S.D. = 1.14, with higher scores indicating greater intention. Data from the present sample show that internal consistency was good, Cronbach's α = .97, rs > .94.

Study 2

Measures

Pre-test Measures

Pre-test covariates were measured as for study 1. Intention to ask for breast cancer screening was asked during the pre-test with the three items applied in Study 1.

Trait Regulatory Orientation. The Regulatory Focus Questionnaire,[9] was applied in the pre-test phase. The questions asked how frequently several specific events occur in the participant's life. Six questions capture the promotion focus, and the other five the prevention focus. Participants replied on a 5-point scale from 1 (never) to 5 (very often). The scores for promotion and prevention scales were calculated averaging the answers on given items after reverse score: data show good internal consistency for both promotion, $\alpha = .66$, rs > .33, and prevention, $\alpha = .74$, rs > .47. The individual's chronic orientation was calculated by subtracting promotion score to prevention score,[9].

Experimental manipulation

Video Messages. Six video-messages were developed for the present study:

- Two video-messages emphasising prevention concerns;
- Two video-messages emphasising promotion concerns;
- Two video-messages without any prompt to regulatory orientation.

Supplemental Table 2 shows the content of the voice-text of the six video-messages.

Post-test Measures

Intention to ask for breast cancer screening. As for Study 1. Participants' scores ranged 1-5, M = 2.99, and S.D. = 1.5, with higher scores indicating greater intention

Supplemental Table 2: voice-text of the video messages for Study 2.

	STUDY 2		
	Promotion video-messages	Prevention video-messages	Control video-messages
VIDEO 1	For the early detection of breast cancer, experts recommend mammography to women aged 50 and over. Mammography is the most effective medical examination for the early detection of breast cancer. It consists of an X-ray exposure that allows you to identify even very small tumors, before they are palpable or recognizable. Women over the age of 50 are invited to undergo a mammogram every 2 years at an accredited radiology center. For women between 50 and 69, the benefits of the exam outweigh the risks. And before the age of 50?		
VIDEO 1	**To respect their health, women under 50 are excluded from the breast cancer screening program. (PROMOTION FOCUS)**	**To avoid adverse health effects, women under 50 are excluded from the breast cancer screening program. (PREVENTION FOCUS)**	**Women under 50 are excluded from the breast cancer screening program. (CONTROL GROUP)**
VIDEO 1	It is a medical recommendation: before the age of 50, the risks of the examination are greater than the benefits.		
VIDEO 1	**In the absence of proven risk, to take care of your health, doctors advise to not undergo a mammogram before the age of 50. (PROMOTION FOCUS)**	**In the absence of proven risk, to avoid adverse effects for your health, doctors advise to not undergo a mammogram before the age of 50. (PREVENTION FOCUS)**	**In the absence of proven risk, doctors advise to not undergo a mammogram before the age of 50. (CONTROL GROUP)**
VIDEO 1	Mammography is a breast test that allows you to detect even many small tumours. Over 50 years, it is done every two years. Women under the age of 50 are excluded from the breast cancer screening program, except in case of genetic predisposition or family history of breast cancer. What are the reasons for this decision?		
VIDEO 1	**The observance of the age threshold determines a decreasing of the probability of false positives: breast anomalies that are benign form. (PROMOTION FOCUS)**	**The observance of the age threshold allows avoiding the negative consequences caused by false positives: breast abnormalities that are benign form. (PREVENTION FOCUS)**	**In young women, false positives are highly likely. False positive are breast anomalies that are benign form. (CONTROL GROUP)**
VIDEO 1	The risk of false positives in young women is higher, because the breast tissue is denser.		
VIDEO 1	**It is advised not to make mammograms before the age of 50 in order not to expose themselves to anti-cancer treatments not recommended as they are often directed to benign anomalies. (PROMOTION FOCUS)**	**It is advisable not to make mammograms before the age of 50 to avoid exposure to non-recommended anti-cancer treatments as they are often directed to benign anomalies. (PREVENTION FOCUS)**	**The breast cancer screening could lead to an exposition of non-recommended anti-cancer treatments, as they are often directed to benign anomalies. (CONTROL GROUP)**
VIDEO 1	In fact, breast cancer is much rarer in women under the age of 50.		
VIDEO 1	**Excluding younger women from screening allows them to be protected from unnecessary radiation exposure. Furthermore, this choice promotes psychological well-being against stress and anxiety. For these reasons, mammographic screening involves only women over 50 years. If you are under 50 and want to take care of your health, we recommend that you respect the age threshold. (PROMOTION FOCUS)**	**Excluding younger women from screening allows you to avoid unnecessary radiation exposure. Furthermore, this choice avoids psychological discomforts such as stress and anxiety. For these reasons, mammographic screening involves only women over 50 years. If you are under 50 years old and want to avoid negative consequences for your health, we recommend that you respect the age threshold. (PREVENTION FOCUS)**	**Radiation exposure poses health risks. In the case of mammography, the exposure is minimal, but for women under the age of 50 the risk is higher than the benefits. Furthermore, as any other medical procedure, waiting for the outcome can generate anxiety and stress. For these reasons, mammographic screening involves only women over 50 years. If you are under 50 years old, we recommend that you respect the age threshold. (CONTROL GROUP)**
VIDEO 1	A conscious prevention, it's worthit!		

Note: grey rounded rectangles show the common parts of the video-messages; the orange rounded rectangles show the promotion video-message specific parts (text in bold); the blue rounded rectangles show the prevention video-message specific parts (text in bold); the green rounded rectangle shows the content of the control leaflet.

References

[1]Shim, M., Kelly, B., & Hornik, R. (2006). Cancer information scanning and seeking behavior is associated with knowledge, lifestyle choices, and screening. *Journal of Health Communication, 11(2),* 157–172.

[2]Daley, M. B., Lerman, C. L., Ross, E., Schwartz, M. D., Sands, C. B., & Masny, A. (1996). Gail model breast cancer risk components are poor predictors of risk perception and screening behaviour. *Breast Cancer Research and Treatment, 41,* 59–70.

[3]Champion, V. L., Skinner, C. S., Menon, U., Rawl, S., Giesler, R. B., Monahan, P., & Daggy, J. (2004). A breast cancer fear scale: Psychometric development. *Journal of Health Psychology, 9(6),* 753-762.

[4] Labrie, N. H., Ludolph, R. A., & Schulz, P. J. (2020). Mammography perceptions and practices among women aged 30–49: The role of screening programme availability and cultural affiliation. Patient Education and Counseling, 103(2), 369-375.

[5]Zaichkowsky, J. L. (1994). The personal involvement inventory: Reduction, revision, and application to advertising. Journal of Advertising, 23 (4), 59-70.

[6]Champion, V. L., Mohahan, P. O., Springston, J. K., Russell, K, Zollinger, T. W., Saywell, R. M. Jr., & Maraj, M. (2008). Measuring mammography and breast cancer beliefs in African American women. *Journal of Health Psychology, 13(6),* 827-837.

[7] Freitas, A. L., & Higgins, E. T. (2002). Enjoying goal-directed action: The role of regulatory fit. *Psychological science*, 13(1), 1-6.

[8]Hersch J., Barratt A., Jansen J., Irwig L., McGeechan K., Jacklyn G., & McCaffery K. (2015). Use of a decision aid including information on over detection to support informed choice about breast cancer screening: a randomized controlled trial. *The Lancet*, *385(9978),* 1642-1652.

[9] Higgins, E. T., Friedman, R. S., Harlow, R. E., Idson, L. C., Ayduk, O. N., & Taylor, A. (2001). Achievement orientations from subjective histories of success: promotion pride versus prevention pride. *European Journal of Social Psychology, 31(1),* 3-23.
