# Supplementary material for: Application of the theory of regulatory fit to promote adherence to evidence-based breast cancer screening recommendations: experimental versus longitudinal evidence
Source: BMJ Open. 2020 Nov 12;10(11):e037748. doi: 10.1136/bmjopen-2020-037748 (PMC7662420; doi:10.1136/bmjopen-2020-037748)
Supplement: Author's manuscript [file bmjopen-2020-037748.draft_revisions.pdf]

BMJ Open

BMJ Open is committed to open peer review. As part of this commitment we make the peer review history of every article we publish publicly available.

When an article is published we post the peer reviewers' comments and the authors' responses online. We also post the versions of the paper that were used during peer review. These are the versions that the peer review comments apply to.

The versions of the paper that follow are the versions that were submitted during the peer review process. They are not the versions of record or the final published versions. They should not be cited or distributed as the published version of this manuscript.

BMJ Open is an open access journal and the full, final, typeset and author-corrected version of record of the manuscript is available on our site with no access controls, subscription charges or pay-per-view fees (http://bmjopen.bmj.com).

If you have any questions on BMJ Open's open peer review process please email info.bmjopen@bmj.com

BMJ Open

An Application of the Theory of Regulatory Fit to Promote Adherence to Evidence-Based Breast Cancer Screening Recommendations: Experimental vs. Longitudinal Evidence

Journal:	*BMJ Open*
Manuscript ID	bmjopen-2020-037748
Article Type:	Original research
Date Submitted by the Author:	18-Feb-2020

Complete List of Authors:	Petrocchi, Serena; Università della Svizzera Italiana,<br>Ludolph, Ramona; Università della Svizzera Italiana<br>Labrie, Nanon ; Vrije Universiteit Amsterdam, Athena Institute, Faculty of Beta Sciences<br>Schulz, Peter; Universita della Svizzera Italiana, Institute of Communication & Health

Keywords:	PUBLIC HEALTH, Breast tumours < ONCOLOGY, PREVENTIVE MEDICINE

SCHOLARONE™
Manuscripts

BMJ

I, the Submitting Author has the right to grant and does grant on behalf of all authors of the Work (as defined in the below author licence), an exclusive licence and/or a non-exclusive licence for contributions from authors who are: i) UK Crown employees; ii) where BMJ has agreed a CC-BY licence shall apply, and/or iii) in accordance with the terms applicable for US Federal Government officers or employees acting as part of their official duties; on a worldwide, perpetual, irrevocable, royalty-free basis to BMJ Publishing Group Ltd ("BMJ") its licensees and where the relevant Journal is co-owned by BMJ to the co-owners of the Journal, to publish the Work in this journal and any other BMJ products and to exploit all rights, as set out in our licence.

The Submitting Author accepts and understands that any supply made under these terms is made by BMJ to the Submitting Author unless you are acting as an employee on behalf of your employer or a postgraduate student of an affiliated institution which is paying any applicable article publishing charge ("APC") for Open Access articles. Where the Submitting Author wishes to make the Work available on an Open Access basis (and intends to pay the relevant APC), the terms of reuse of such Open Access shall be governed by a Creative Commons licence – details of these licences and which Creative Commons licence will apply to this Work are set out in our licence referred to above.

Other than as permitted in any relevant BMJ Author's Self Archiving Policies, I confirm this Work has not been accepted for publication elsewhere, is not being considered for publication elsewhere and does not duplicate material already published. I confirm all authors consent to publication of this Work and authorise the granting of this licence.

For peer review only

An Application of the Theory of Regulatory Fit to Promote Adherence to Evidence-Based Breast Cancer Screening Recommendations: Experimental vs. Longitudinal Evidence

Petrocchi, Serena, Ludolph, Ramona, Labrie, Nanon H.M., Schulz, Peter J.

Corresponding author

Dr. Serena Petrocchi

Institute of Communication and Health

Università della Svizzera italiana

Via Buffi 13

6900 Lugano

Switzerland

T: 0041 (0)58 666 4821

Fax: 0041 (0)58 666 4000

E: serena.petrocchi@usi.ch

Prof. Peter J. Schulz

Institute of Communication & Health

Università della Svizzera italiana

Via Buffi 13

6900 Lugano

Switzerland

Dr. Ramona Ludolph:

Institute of Communication & Health

Università della Svizzera italiana

Via Buffi 13

6900 Lugano

Switzerland

For peer review only - http://bmjopen.bmj.com/site/about/guidelines.xhtml

Dr. Nanon H.M. Labrie
Athena Institute, Faculty of Beta Sciences
Vrije Universiteit Amsterdam
De Boelelaan 1105, 1081 HV Amsterdam
The Netherlands: n.h.m.labrie@vu.nl

Keywords: Breast Cancer Screening; Theory of Regulatory Fit; Breast Cancer Prevention; Mammography

Word count: 4168 words

Declaration of authors' contribution

SP wrote the manuscript and all authors read and checked its draft and final version. RL wrote part of the introduction. PJS acquired funding. NHML, PJS, and RL designed Study 1 and prepared the materials. PJS, SP, and RL designed Study 2 and prepared the materials. SP and RL collected data for Study 1. SP collected data for Study 2. SP performed the analyses for Study 1 and Study 2.

For peer review only - http://bmjopen.bmj.com/site/about/guidelines.xhtml

An Application of the Theory of Regulatory Fit to Promote Adherence to Evidence-Based Breast Cancer Screening Recommendations: Experimental vs. Longitudinal Evidence

For peer review only - http://bmjopen.bmj.com/site/about/guidelines.xhtml

Abstract

Objectives. Reducing mammographic screening under the age of 50 without medical reasons is a public health concern. Following principles of the theory of regulatory fit, two studies investigated whether messages in regulatory fit with study participants' orientation were more persuasive than messages without such fit. **Design**. Study 1 was an experimental study in which promotion or prevention focus was primed, and then participants were exposed to a video message that fitted with the induced focus. A control group received a stimulus without focus. We compared women on promotion fit vs. prevention fit vs. control condition on their intention to seek mammography unless medically indicated. Study 2 added a longitudinal component to Study 1. The regulatory trait was measured through a questionnaire, and comparisons among fit vs. non-fit vs. control conditions were performed. **Participants**. Three hundred sixty women participated in Study 1, 292, in Study 2. They were from 30 to 45 years of age, with no history of breast cancer or BRCA 1/2 mutation. **Results**. In Study 1, regulatory fit decreased the intention to seek mammography unless medically indicated in women under 50. Study 2, however, did not show such an effect. In both studies, age, risk perception, and fear of breast cancer were significant covariates. **Conclusions**. Future research should consider how reducing the impact of negative emotions rather than try to overcome its effect when promoting adherence to evidence-based breast cancer screening recommendations.

Keywords: Breast Cancer Screening; Theory of Regulatory Fit; Breast Cancer Prevention; Mammography

For peer review only - http://bmjopen.bmj.com/site/about/guidelines.xhtml

Strengths and limitations of this Study

- Principles of the Theory of Regulatory Fit are applied in an experimental study (Study 1) and an experimental study with a longitudinal component (Study 2)
- The regulatory focus was primed in Study 1 and estimated through a questionnaire in Study 2
- Messages were tailored creating a regulatory fit (vs. non-fit) between the content of the message and the individual's orientation
- Limitations of the studies included high dropout rates, especially in Study 2, and selection bias (possibly due to fear of cancer)
- Reducing the number of unnecessary breast cancer screening must keep into account the role played by negative emotions

For peer review only - http://bmjopen.bmj.com/site/about/guidelines.xhtml

An Application of the Theory of Regulatory Fit to Promote Adherence to Evidence-Based Breast Cancer Screening Recommendations: Experimental vs. Longitudinal Evidence

Breast cancer is one of the most common forms of cancer in women worldwide and the principal cause of cancer-related death in the female population (Torre, Islami, Siegel, Ward, & Jemal, 2017). To promote early diagnosis, many EU Countries have initiated systematic breast cancer screening programs (Altobelli & Lattanzi, 2014). Regular screening from 50 to 70 years of age can help the early detection of breast cancer. Findings for women under the age of 50 did not find significant effects of breast cancer screening on reducing mortality rates due to breast cancer (Gøtzsche & Jørgensen, 2013). On the other hand, possible harms associated with regular mammography below the age of 50 are recognized (Armstrong, Moye, Williams, Berlin, & Reynolds, 2007; Barratt, Howard, Irwig, Salked, & Houssami, 2005; Gøtzsche & Nielsen, 2006; Gøtzsche & Jørgensen, 2013).

Nonetheless, many women below the age of 50 seek and receive screenings without medical reasons indicating it (Block, Jarlenski, Wu, & Bennett, 2013; Glaus, Fäh, Hornung, Senn, & Stiefel, 2004; Kapp, Reyerson, Couchlin, & Thompson, 2009; Klug, Hetzer, & Blettner, 2005; blind for review; Statistics Netherlands, 2015). Women tend to overestimate the mortality reduction determined by breast cancer screening (Chamot & Perneger, 2001; Gigerenzer, Mata, & Frank, 2009) and have unrealistic expectations regarding the breast cancer screening as reducing the risk of breast cancer (Domenighetti et al., 2003). Therefore, laypeople could consider counterintuitive the recommendation to avoid medically not indicated breast cancer screening, although scientifically sustained, because it violates the belief that cancer

For peer review only - http://bmjopen.bmj.com/site/about/guidelines.xhtml

screening can save lives. Assuming that a substantial part of breast cancer screening below the age of 50 is not due to medical indications, to promote the adherence to evidence-based recommendations on breast cancer screening among young women seems to be a vital research mandate. A way to overcome the impact of an individual's involvement and negative emotions as motivational factors for high breast cancer screening demand could arise by the activation of an alternative motivation system, such as the regulatory orientation (Higgins, 2000).

Theory of Regulatory Focus and Regulatory Fit

The Theory of Regulatory Focus (Higgins, 1997) states that people's regulatory orientation is a motivational principle, which influences behavioural choices, and it is characterized either by a promotion or by a prevention orientation. While individuals with a promotion focus are described as eagerly pursuing their goals and striving towards the realization of desired outcomes, those with a prevention focus are defined as being safety-driven and vigilant to prevent errors and undesired results (Higgins, 1997; Keller, 2006). The regulatory focus orientation can be primed (Cesario, Higgins, & Scholer, 2008; Freitas & Higgins, 2002; Higgins, 1997) or either estimated through questionnaires (Higgins et al., 2001).

A phenomenon called 'regulatory fit' occurs (Higgins, 2000) when a goal pursuit strategy matches the regulatory focus orientation. Behaviours shown under the conditions of a regulatory fit are perceived as adequate and rewarding (Cesario, Grant, & Higgins, 2004). The effects of regulatory fit have been extensively studied in the context of disease prevention and health promotion (Spiegel, Grant-Pillow, & Higgins, 2004; Zhao & Pechmann, 2007). Uskul, Keller, and Oyserman (2008) were among the first to apply the principles of regulatory fit to disease detection and, particularly, to inform people about the benefits of regular cancer screenings.

The purpose of the present research was to test whether health messages framed to correspond with a woman's regulatory focus orientation are effective in *reducing* the intention to ask for medically not indicated breast cancer screening under the age of 50, challenging lay people's common sense. Achievement from the present research would be twofold. Theoretically, significant results will improve knowledge on the impact that regulatory orientation, as a motivational system, has when applied to a counterintuitive topic for laypeople. The practical implication will include the possibility to reduce the demand for regular mammography without medical indications and, then, to moderate the possible harms associated.

Two studies were developed. Study 1 tested the hypothesis that the fit between the message frame and the women's regulatory orientation would reduce their intention to ask for medically not indicated breast cancer screening. Study 2 longitudinally tested the same association comparing two fit conditions vs. two non-fit conditions vs. a control condition. It was expected that the fit conditions would lead to a reduction of the intention to ask for medically not indicated breast cancer screening compared to the non-fit conditions and the control group.

Study 1

Methods

Participants

An a priori power analysis applying G*Power 3.1.9.2 (Faul, Erdfelder, Lang, & Buchner, 2007) estimated a sample of 249 participants ($\alpha = .05$, $d = .95$, $\eta^2 = 0.05$; see blind for review). Five hundred women from 30 to 45 years started the survey: 121 (16%) initiated the pre-test questionnaires but dropped out. Nineteen of the women were excluded from the final sample because they did not complete the experimental manipulation. Participants lived in Ticino, the Italian-speaking canton of Switzerland. No differences emerged in the pre-test variables between those who filled in only the

For peer review only - http://bmjopen.bmj.com/site/about/guidelines.xhtml

pre-test (N = 140) and who filled in the entire survey (N = 360). Participants were randomly assigned to prevention fit, promotion fit, and control condition (see Table 1). No differences were found between the intervention groups and the control group on socio-demographic variables.

[Insert Table 1 here]

Procedure

A pre-post-test design with two experimental conditions and a control group was applied (see Table 2 for full details).

[insert Table 2 here]

After the pre-test questionnaires, participants were randomized into promotion fit, prevention fit, or control condition. In the fit conditions, the two regulatory foci were primed (Higgins, 1997) and then followed by video-messages fitting with the primed focus. Immediately after priming, participants in the promotion fit condition watched a video message emphasizing promotion concerns (i.e., they should adhere to evidence-based recommendations on mammography screening for safety and health protection reasons). Participants in the prevention fit condition watched a video emphasizing prevention concerns (i.e., they should not abstain from following the evidence-based recommendations on mammography screening to avoid negative/side effects). Participants in the control group did not receive any priming and read a general health leaflet. Table 3 shows the content of the video messages and leaflet. In a pilot study, 30 women assessed the survey as clear and understandable.

[insert Table 3 here]

Participants replied to an online survey from June to September 2016. The research was repeatedly advertised on the Facebook page of the University and by public/private organizations in Ticino. Exclusion criteria were: a personal history of breast cancer, BRCA mutations, insufficient fluency in Italian. Women aged 46-49

For peer review only - http://bmjopen.bmj.com/site/about/guidelines.xhtml

were excluded to avoid discouraging them from undertaking breast cancer screening once they turned 50. The University's Ethical Committee approved Study 1 and Study 2. Participants received a 10 CHF supermarket voucher as compensation. Patients or the public were not involved in the design, or conduct, or reporting, or dissemination plans of our research.

Patient and Public Involvement

Results from previous studies involving participants from Switzerland informed the present research. In particular, the research questions and the outcome measures of the present research have been informed by patients' priorities, experience, and preferences collected by Author and colleagues (blind for review). Participants were not directly involved in the design, or conduct, or reporting, or recruitment, or dissemination of our research. Participants agreed to receive a summary of the essential results via email. The results of the whole sample were presented to the participants without any personal details.

Measures

Pre-test measures. Questions were posed on overall health status and health behaviours (i.e., diet, physical activity, smoking habits, alcohol consumption; see Shim, Kelly, & Hornik, 2006). Participants replied then to a set of questions on a past diagnosis of breast cancer among first-grade relatives (Daley et al., 1996). They also reported if they had a mammography in the past, if a doctor recommended mammography to them, if they had a breast biopsy, and if they know the breast cancer screening program in Ticino. Moreover, the women rated their fear for breast cancer (Champion et al., 2004; blind for review; data from the present sample show acceptable internal consistency, $\alpha = .88$, $rs > .73$, as well as the factor structure, χ^2 (1) = 2.04, $p = .15$, $CFI = .99$, $RMSEA = .05$). The Personal Involvement Inventory (Zaichkowsky, 1994) was administered as a 7-digit semantic differential. Data from

For peer review only - http://bmjopen.bmj.com/site/about/guidelines.xhtml

the present sample show acceptable internal consistency, $\alpha = .91$, $rs > .71$, as well as the factor structure, χ^2 (5) = 11.34, $p = .04$, $CFI = .99$, $RMSEA = .06$. Finally, four items measured the perceived benefit of mammography screening (Champion et al., 2008). Data from the present sample show modest internal consistency, $\alpha = .75$, $rs > .49$ and an acceptable factor structure, χ^2 (1) = .51, $p = .47$, $CFI = 1$, $RMSEA = .00$.

Regulatory Focus Induction. Regulatory fit manipulation was induced by completing the regulatory fit questionnaire (Freitas & Higgins 2002). Prevention induced participants were asked to list two of their current obligations and then write down five actions they could take to avoid failure in fulfilling them. Promotion induced participants were asked to list two aspirations and write down five actions they could take to ensure their accomplishment.

Intention to ask for breast cancer screening. Three items measured the intention to have a mammography screening for breast cancer in the next 2-3 years were applied (the measure shows acceptable internal consistency, $\alpha = .97$, $rs > .94$).

Analytic Strategy

Data were normalized through reverse scoring and logarithmic transformations. There were not missing data.

Results

The ANCOVA [$F(2, 319) = 49.57$, $p < .0001$, $\eta^2_p = .24$] revealed that the promotion fit condition, $t(319) = -8.80$, $p < .0001$, $r = .44$, as well as the prevention fit condition, $t(319) = -8.80$, $p < .0001$, $r = .44$, were both associated with lower intentions to ask for breast cancer screening compared to the control condition. There was no significant difference between the two intervention groups ($p > .05$). See Table 4 for descriptive statistics. Among the covariates, fear of breast cancer [$F(1, 319)$ = 6.81, $p = .010$, partial $\eta^2_p = .02$] and age [$F(1, 319) = 26.20$, $p < .0001$, partial η^2_p = .08] were positively related to the intention to ask for breast cancer screening.

For peer review only - http://bmjopen.bmj.com/site/about/guidelines.xhtml

[Insert Table 4 here]

A significant association between promotion fit/control condition and past diagnoses of breast cancer among first grade relatives was found, χ^2 (2) = 12.98, p = .002. Women in the promotion fit condition had a lower number of breast cancer diagnoses among first grade relatives then was expected (z = -1.96); while women in the control condition had a higher number than the expected (z = 2.8). An ANCOVA was conducted to test whether past diagnoses of breast cancer among first grade relatives may be a significant covariate. The analysis (N = 230) revealed significant effects on post-test intention of both the promotion fit manipulation [F(1, 190) = 6.56, p = .011, η2p = .03] and the past diagnoses of breast cancer among first grade relatives [F(1, 190) = 5.81, p = .017, η2p = .03]. Women in the promotion group showed lower intention to ask for breast cancer screening than women in the control group. Past diagnoses of breast cancer among first grade relatives increases the intention to ask for breast cancer screening (M= 3.59, SD = 1.55) comparing to the opposite condition (M = 2.79, SD = 1.35). The interaction between experimental manipulation*past diagnoses was not significant (p > .05).

Discussion

Limitations of Study 1 include that women received the intervention one time. Study 2 was designed to overcome this limitation.

Study 2

Methods

Participants

An a priori power analysis was calculated as for Study 1, and a sample size of 312 was estimated. Nine hundred seventy-three women from 30 to 45 filled in the pre-test questionnaires (i.e., pre-test sample). Completed questionnaires (i.e., analytical sample) were returned from 292 women with an attrition rate of 70%. Comparisons

For peer review only - http://bmjopen.bmj.com/site/about/guidelines.xhtml

between the pre-test sample and the analytical sample did not yield significant differences. The 292 participants were randomly assigned to five conditions: promotion fit, promotion non-fit, prevention fit, prevention non-fit, and control condition (see Table 1). Women aged 30 to 45 living in Ticino and Italy participated. Italian and Ticinese-Swiss participants share the same culture and language and follow the same rules for their breast cancer screening programs. No differences were found among the five groups regarding socio-demographic variables or other pre-test variables.

Procedure

A pre-post-test longitudinal design was applied with four experimental conditions, two fit conditions (promotion and prevention), two non-fit conditions (promotion and prevention), and a control group (see Table 2). In the pre-test (T0), participants replied to a set of questions comprising socio-demographic variables, covariates, and a questionnaire measuring women's regulatory focus. The latest was applied because working with the trait regulatory focus would be more stable than a primed focus in a longitudinal design. Participants were randomly assigned to the fit or non-fit condition or the control group. In the control group, half of them had a promotion orientation and half prevention orientation. Participants in the fit conditions watched two videos (T1 and T2) emphasizing the fit concerns (see Table 5). In the non-fit conditions, participants watched two videos (T1 and T2) emphasizing the non-fit concerns. In the control group, participants watched two videos (T1 and T2) treating the topic of breast cancer prevention, but without any regulatory prompt. A post-test questionnaire evaluates women's' intention to ask for opportunistic screening (T3). Ten days elapsed between each experimental phase.

[Insert Table 5 here]

For peer review only - http://bmjopen.bmj.com/site/about/guidelines.xhtml

Two health communication professionals with expertise on regulatory focus evaluated the voice-over and the graphical aspects of the videos. Three women from the general population assessed the videos as understandable and clear.

The research was advertised through the Facebook page of the University and by RCSMedia Group, an Italian-based publishing group that uses participant panels. Inclusion/exclusion criteria were as for study 1. Recruitment took place from June to October 2017. Participants included in Study 1 could not participate in Study 2. They received a 10 CHF/EU supermarket voucher. The final survey was tested by fifteen women aged 30 to 45, who assessed it as clear and understandable.

Patient and Public Involvement

As for Study 1.

Measures

Pre-test covariates were measured as for study 1; the intention was measured both in the pre-test and post-test.

Trait Regulatory Orientation. The Regulatory Focus Questionnaire (Higgins et al., 2001) was applied in the pre-test phase. The questions asked how frequently several specific events occur in the participant's life. Six questions capture the promotion focus, and the other five the prevention focus. Participants replied on a 5-point scale from 1 (never) to 5 (very often). The scores for promotion and prevention scales were calculated averaging the answers on given items: data show good internal consistency for both promotion, $\alpha = .66$, $rs > .33$, and prevention, $\alpha = .74$, $rs > .47$. The individual's chronic orientation was calculated following the original procedure (see Higgins et al., 2001).

Analytic Strategy

Data were normalized through reverse scoring and logarithmic transformations. There were not missing data. Repeated measures ANCOVA was applied.

For peer review only - http://bmjopen.bmj.com/site/about/guidelines.xhtml

Results

Replication of Study I

The within-subject effect reached significance, $F(1, 268.25) = 5.34$, $p = .021$, with a general decreasing of the intention from the pre-test (M = 3.38 , SD = 1.32) to the post-test measurement (M = 3, SD = 1.52). The intervention effect was not significant, $F(2, 286) = .05$, $p > .05$, and among the covariates, fear of breast cancer, $t = 2.87$, $p = .004$, $\beta = .25$, 95%Low CI = .09, 95%High CI = .40, risk perception, $t = 2.22$, $p = .027$, $\beta = .01$, 95%Low CI = .001, 95%High CI = .019, and age, $t = 6.36$, $p < .001$, $\beta = .11$, 95%Low CI = .07, 95%High CI = .15, were positively related to post-intention.

Main Analyses

There was no significant effect of the intervention, indicating that the scores of post-test intention among the five groups were in general the same, $F(4, 284) = .43$, $p > .05$. There was a significant within subjects effect, $F(1, 267.91) = 5.10$, $p = .025$, partial $\eta^2 = .02$, indicating that there was a general decrease of the intention from pre- to post-evaluation across groups. Among the covariates, fear of breast cancer, $t(284) = 2.76$, $p = .006$, $B = .24$, partial $\eta^2 = .03$ (95% Low CI = .07, 95% High CI = .42), age, $t(284) = 6.26$, $p < .0001$, $B = .11$, partial $\eta^2 = .12$ (95% Low CI = .08, 95% High CI = .15), and risk perception, $t(284) = 2.26$, $p = .024$, $B = .37$, partial $\eta^2 = .02$ (95% Low CI = .05, 95% High CI = .70), significantly increased post-test intention. See Table 4 for descriptive statistics.

The intervention effect was not significant either when the two fit conditions and the two non-fit conditions were collapsed in two categories (i.e., comparison among fit condition vs. un-fit condition vs control), $F(2, 289) < 1$, $p > .05$. There was a general decrease of the post-intention across groups, $F(1, 268.48) = 5.34$, $p = .022$, partial $\eta^2 = .02$, and significant effects of age, $t(286) = 6.26$, $p < .0001$, $B = .11$, partial $\eta^2 = .12$

(95% Low CI = .08, 95% High CI = .15), fear of breast cancer, t(286) = 2.82, p = .005, B = .25, partial η^2 = .03 (95% Low CI = .08, 95% High CI = .42), and risk perception, t(286) = 2.28, p = .023, B = .37, partial η^2 = .02 (95% Low CI = .05, 95% High CI = .70), in increasing the post-test intention. Risk perception was tested as a moderator, but the analysis did not reach significance.

Discussion

The application of regulatory fit in the area of health communication is beneficial across various health contexts and outcomes (blind for review). Previous research has applied those principles to disease detection intending to influence individuals' intention to engage in regular cancer screenings (Uskul, Keller, & Oyserman, 2008). However, no previous studies have tested messages designed according to the assumptions of regulatory fit to influence the intention to *not* engage in disease detection.

The present research shows inconsistent results. Study 1 confirmed the hypothesized effect of the intervention on the intention to seek mammography unless medically indicated, with a reduction of the intention when a fit between the message frame and the individual's regulatory focus occurred. However, evidence collected from Study 1 should be cautiously considered. Longitudinal results from Study 2 demonstrated that this effect was not significant over one month, although a general decrease of the intention across groups was observed. Further evidence is needed to confirm our longitudinal results. Still, it seems that the 'just-feels-right' experience appears to be not enough to convince women to avoid breast cancer screening without medical indications.

Our results could genuinely reflect the fact that the regulatory fit is not sufficient to induce a decrease in the women's intention or could be an artefact of the research itself. Study 1 and Study 2 applied two different ways to evoke a regulatory

For peer review only - http://bmjopen.bmj.com/site/about/guidelines.xhtml

orientation. Study 1 primed the individuals' regulatory orientation, whereas Study 2 measured it with a questionnaire. One could argue that the different ways to induce vs. measure the regulatory orientation could have influenced the persuasiveness of the message and so its effectiveness. However, researchers of regulatory orientation suggest that there is no difference between the two procedures (Cesario, Higgins, & Scholer, 2008). Therefore, we could exclude that the two methods have had a differential impact on post-test intention.

The relatively low sample size and the recruitment strategies could have influenced the power of the analyses, the sample composition, and, ultimately, the significance of the results. However, there is no power concern in Study 2 since the effect due to the intervention was not significant either when the two fit conditions and the two non-fit conditions were collapsed in two categories.

Finally, a variable might have moderated the association between intervention and intention. As Kees (2011) has demonstrated in their research, the individuals' consideration of future consequences of a particular behaviour has influenced the effectiveness of framing techniques in predicting risk perceptions, attitudes, and behavioural intentions regarding health-related advertisements. In our research, the risk perception was tested as a moderator variable, but the analyses yielded no significant results.

Fear of breast cancer, age, and risk perception (only in Study 2) were significantly related to women's intention. The predicting role of age is not surprising because, approaching the age of 50, women are invited to undertake regular mammography screening in Ticino. Risk perception and fear of breast cancer are the most sensitive variables. Breast cancer naturally evokes negative emotions (Consedine, Magai, Krivoshekova, & Ryzewicz, 2004; Hay, McCaul, & Magnan, 2006; blind for review; Nekhlyudov, Ross-Degnan, & Fletcher, 2003). Moreover, the benefits of

For peer review only - http://bmjopen.bmj.com/site/about/guidelines.xhtml

mammography screening are often overestimation (Chamot & Perneger, 2001; Domenighetti et al., 2003). Therefore, it is challenging to develop effective health messages dealing with the downsides of medically not indicated breast cancer screening based on factual information. As messages based on the principles of regulatory fit take the motivational orientations of recipients into account, they go beyond the effectiveness of purely providing information. Here, messages building on the theory of regulatory fit did not seem to offer a new way to overcome the 'emotional barrier' generated by the fear of breast cancer. However, study 2 demonstrated a general 'pedagogical effect' deriving from talking about the topic of breast cancer screening without evoking boomerang effect (i.e., an increase of intention instead of a decreasing).

The present research has several limitations. We experienced high dropout rates, especially in Study 2. The high dropout rates may be related to the topic of breast cancer itself or the fear associated with it. One could assume that women with a low level of fear of breast cancer may have decided to do not take part in our research, and this may have created a selection bias that could affect the generalizability of the results.

In conclusion, it seems that by framing health messages, conforming to a promotion or prevention focus, a decreasing of the intention to ask for merely preventive opportunistic mammography screening is observed, but only immediately after the exposure to the message. The influence decreases over time, and the messages lose their predictive effects after one month.

Even though our results did not confirm our hypothesis, they still have substantial implications for future research. The results demonstrated that fear of breast cancer and risk perception are the main challenges to face to promote adherence to evidence-based recommendations on breast cancer screening. Public health must

For peer review only - http://bmjopen.bmj.com/site/about/guidelines.xhtml

investigate what factors may increase the effectiveness of health information. Therefore, according to our evidence, future research may consider understanding how reducing the impact of negative emotions rather than try to overcome its effect. For example, Nabi (2016) found that humour in health messages reduces the anxiety associated with performing cancer screening. Humour may be implemented in health messages aimed to promote evidence-based breast cancer screening recommendations. Reducing the number of unnecessary breast cancer screening would thus allow the prevention of avoidable false positive and false negative diagnoses and unjustified mental and physical suffering for women. In the long term, this would also enable policy-makers and health professionals to allocate scarce resources for disease prevention, detection, and cure in a more effective way.

Funding Statement

This work was supported by the Swiss National Science Foundation [grant number FNS 100019-153131/1].

Competing interests statement

None declared.

Author contributions

SP wrote the manuscript, and all authors read and checked its draft and final version. RL wrote part of the introduction. PJS acquired funding. NHML, PJS, and RL designed Study 1 and prepared the materials. PJS, SP, and RL designed Study 2 and prepared the materials. SP and RL collected data for Study 1. SP collected data for Study 2. SP performed the analyses for Study 1 and Study 2.

References

For peer review only - http://bmjopen.bmj.com/site/about/guidelines.xhtml

Altobelli, E., & Lattanzi, A. (2014). Breast cancer in European Union: An update of screening programs as of March 2014 (Review). *International Journal of Oncology, 45(5),* 1785–1792.

Armstrong, K., Moye, E., Williams, S., Berlin, J. A., & Reynolds, E. E. (2007). Screening mammography in women 40 to 49 years of age: a systematic review for the American College of Physicians. *Annals of Internal Medicine, 146,* 516–26.

Barratt, A. L., Howard, K., Irwig, L., Salked, G., & Houssami, N. (2005). Model of outcomes of screening mammography: information to support informed choices. *British Medical Journal, 330,* 936–938.

Block, L. D., Jarlenski, M. P., Wu, A. W., & Bennett, W. L. (2013). Mammography use among women ages 40-49 after the 2009. US Preventive Services Task Force recommendation. *Journal of General Internal Medicine, 28(11),* 1447-1453.

Cesario, J., Grant, H., & Higgins, E. T. (2004). Regulatory fit and persuasion: transfer from' feeling right.' *Journal of Personality and Social Psychology, 86(3),* 388-404.

Cesario, J., Higgins, E. T., & Scholer, A. A. (2008). Regulatory fit and persuasion: basic principles and remaining questions. *Social Personality Psychological Compass*, *2*, 444-63.

Chamot, E., & Perneger, T. V. (2001). Misconceptions about efficacy of mammography screening: a public health dilemma. *Journal of Epidemiology & Community Health, 55,* 799-803.

Champion, V. L., Mohahan, P. O., Springston, J. K., Russell, K, Zollinger, T. W., Saywell, R. M. Jr., & Maraj, M. (2008). Measuring mammography and breast

For peer review only - http://bmjopen.bmj.com/site/about/guidelines.xhtml

cancer beliefs in African American women. *Journal of Health Psychology, 13(6),* 827-837.

Champion, V. L., Skinner, C. S., Menon, U., Rawl, S., Giesler, R. B., Monahan, P., & Daggy, J. (2004). A breast cancer fear scale: Psychometric development. *Journal of Health Psychology, 9(6),* 753-762.

Consedine, N. S., Magai, C., Krivoshekova, Y. S., Ryzewicz, L., & Neugut, A. I. (2004). Fear, anxiety, worry, and breast cancer screening behavior: a critical review. *Cancer Epidemiology and Prevention Biomarkers, 13(4),* 501-510.

Daley, M. B., Lerman, C. L., Ross, E., Schwartz, M. D., Sands, C. B., & Masny, A. (1996). Gail model breast cancer risk components are poor predictors of risk perception and screening behaviour. *Breast Cancer Research and Treatment, 41,* 59–70.

Domenighetti, G., D'avanzo, B., Egger, M., Berrino, F., Perneger, T., Mosconi, P., & Zwahlen, M. (2003). Women's perception of the benefits of mammography screening: population-based survey in four countries. *International journal of epidemiology, 32(5),* 816-821.

Faul, F., Erdfelder, E., Lang, A. G., & Buchner, A. (2007). G*Power 3: A flexible statistical power analysis program for the social, behavioural, and biomedical sciences. *Behavior Research Methods*, *39,* 175-191.

Freitas, A. L., & Higgins, E. T. (2002). Enjoying goal-directed action: The role of regulatory fit. *Psychological science, 13(1),* 1-6.

Gigerenzer, G., Mata, J., & Frank, R. (2009). Public knowledge of benefits of breast and prostate cancer screening in Europe. *Journal of the National Cancer Institute, 101(17),* 1216-1220.

Glaus, A., Fäh, B., Hornung, R., Senn, H., & Stiefel, F. (2004). Das Brustkrebs-Präventionsverhalten in der Schweiz: aus der Perspektive von Frauen aus drei

For peer review only - http://bmjopen.bmj.com/site/about/guidelines.xhtml

Sprachregionen der Schweiz. [Breast cancer prevention behaviour: a perspective of women from three language regions of Switzerland]. *Pflege, 17(6)*, 385-394.

Gøtzsche, P. C., & Nielsen, M. (2006). Screening for breast cancer with mammography. *Cochrane Database of Systematic Review*, *4,* CD001877.

Gøtzsche, P. C., & Jørgensen, K. J. (2013). Screening for breast cancer with mammography. *Cochrane Database of Systematic Reviews*, *6*, CD001877.

Hay, J. L., McCaul, K. D., & Magnan, R. E. (2006). Does worry about breast cancer predict screening behaviors? A meta-analysis of the prospective evidence. *Preventive medicine, 42(6)*, 401-408.

Higgins, E. T. (1997). Beyond pleasure and pain. *American Psychologist*, *52(12),* 1280-1300.

Higgins, E. T. (2000). Making a good decision: value from fit. *American Psychologist*, *55(11),* 1217-1227.

Higgins, E. T., Friedman, R. S., Harlow, R. E., Idson, L. C., Ayduk, O. N., & Taylor, A. (2001). Achievement orientations from subjective histories of success: promotion pride versus prevention pride. *European Journal of Social Psychology, 31(1),* 3-23.

Kapp, J. M., Ryerson, A. B., Coughlin, S. S., & Thompson, T. D. (2009). Racial and ethnic differences in mammography use among US women younger than age 40. *Breast Cancer Research and Treatment, 113(2),* 327-337.

Kees, J. (2011). Advertising framing effects and consideration of future consequences. *The Journal of Consumer Affairs, 45(1)*, 7-32.

Keller, P. A. (2006). Regulatory focus and efficacy of health messages. *Journal of Consumer Research, 33(1),* 109-114.

For peer review only - http://bmjopen.bmj.com/site/about/guidelines.xhtml

Klug, S. J., Hetzer, M., & Blettner, M. (2005). Screening for breast and cervical cancer in a large German city: participation, motivation and knowledge of risk factors. *The European Journal of Public Health, 15(1),* 70-77.

Nabi, R. L., (2016). Laughting in the face of fear (of disease detection): using humor to promote cancer self-examination behavior. *Health Communication*, *31(7),* 873-883.

Nekhlyudov, L., Ross-Degnan, D., & Fletcher, S. W. (2003). Beliefs and expectation of women under 50 years old regarding screening mammography: a qualitative study. *Journal of General Internal Medicine, 18(3),* 182-9.

Schwartz, L. M., Woloshin, S., Fowler Jr, F. J., & Welch, H. G. (2004). Enthusiasm for cancer screening in the United States. *Jama, 291(1),* 71-78.

Shim, M., Kelly, B., & Hornik, R. (2006). Cancer information scanning and seeking behavior is associated with knowledge, lifestyle choices, and screening. *Journal of Health Communication, 11(2),* 157–172.

Spiegel, S., Grant-Pillow, H., & Higgins, E. T. (2004). How regulatory fit enhances motivational strength during goal pursuit. *European Journal of Social Psychology, 39,* 39–54.

Statistics Netherlands (2015). CBS: Ruim de helft van de vrouwen laat uitstrijkje maken. [More than half of all women get a pap-smear]. Retrieved from: http://www.cbs.nl/nl-NL/menu/themas/gezondheid-welzijn/publicaties/artikelen/archief/2015/ruim-de-helft-van-de-vrouwen-laat-uitstrijkje-maken.htm

Torre, L.A., Islami, F., Siegel, R.L., Ward, E.M., Jemal, A., (2017). Global cancer in women: burden and trends. *Cancer Epidemiology, biomarkers & Prevention. CEBP Focus: Global Cancer in Women, 26(4),* 444-457.

For peer review only - http://bmjopen.bmj.com/site/about/guidelines.xhtml

Uskul, A. K., Keller, J., & Oyserman, D. (2008). Regulatory fit and health behavior. *Psychology and Health*, *23*(3), 327-346.

Zaichkowsky, J. L. (1994). The personal involvement inventory: Reduction, revision, and application to advertising. *Journal of Advertising, 23 (4),* 59-70.

Zhao, G., & Pechmann, C. (2007). The impact of regulatory focus on adolescents' response to antismoking advertising campaigns. *Journal of Marketing Research, XLIV*, 671-6

For peer review only - http://bmjopen.bmj.com/site/about/guidelines.xhtml

Table 1. Demographics of Study 1 and Study 2.

	Study 1			Study 2				
	**Promotion Fit (N = 122)**	**Prevention Fit (N = 130)**	**Control Group (N = 108)**	**Promotion Fit (N = 58)**	**Promotion Unfit (N = 57)**	**Prevention Fit (N = 74)**	**Prevention Unfit (N = 74)**	**Control Group (N = 29)**
**Age** (range 30-45): M and (SD)	36.55 (4.42)	38.07 (4.57)	38.37 (4.79)	38.1 (4.96)	38.53 (4.7)	38.31 (4.44)	37.93 (4.41)	37.02 (4.99)
**Marital Status**								
Married	73 (59%)	77 (60%)	69 (64%)	36 (62%)	41 (72%)	55 (74%)	53 (72%)	22 (76%)
Single	38 (31%)	38 (30%)	26 (24%)	20 (35%)	12 (21%)	14 (19%)	17 (23%)	6 (21%)
Divorced/Separated/Widowed	11 (10%)	15 (10%)	13 (12%)	2 (3%)	4 (7%)	5 (7%)	4 (5%)	1 (3%)
**Educational Level**								
Elementary/Junior School	2 (2%)	2 (2%)	3 (3%)	1 (2%)	-	1 (1%)	4 (5%)	-
High School	44 (34%)	56 (46%)	58 (54%)	18 (31%)	24 (43%)	40 (54%)	28 (38%)	8 (28%)
University or Post University Degree	84 (64%)	64 (52%)	47 (43%)	39 (66%)	33 (57%)	33 (45%)	42 (57%)	21 (72%)
**Occupation**								
Employed	102 (84%)	107 (82%)	74 (69%)	48 (83%)	50 (88%)	57 (77%)	67 (91%)	29 (90%)
Homemaker	11 (9%)	14 (11%)	22 (20%)	4 (7%)	3 (5%)	7 (9%)	6 (8%)	1 (3%)
Unemployed	8 (6%)	7 (5%)	10 (9%)	4 (7%)	4 (7%)	8 (11%)	1 (1%)	2 (7%)
Student	1 (1%)	2 (2%)	2 (2%)	2 (3%)	-	2 (3%)	-	-
**Nationality**								
Swiss	97 (80%)	101 (78%)	73 (68%)	10 (17%)	15 (26%)	16 (18%)	13 (18%)	-
Italian	21 (17%)	23 (18%)	26 (24%)	47 (81%)	40 (70%)	53 (71%)	58 (78%)	27 (93%)
Other	4 (3%)	6 (4%)	9 (8%)	1 (2%)	2 (4%)	5 (7%)	3 (4%)	4 (7%)
**Mother Tongue**								
Italian	117 (96%)	122 (94%)	94 (87%)	54 (93%)	54 (93%)	68 (92%)	71 (96%)	27 (93%)
Other	5 (4%)	8 (6%)	14 (13%)	4 (7%)	4 (7%)	6 (8%)	3 (4%)	2 (7%)

Table 2. Study 1 and Study 2 design and materials.

STUDY 1

PRE-TEST

Socio-Demo variables
Health Status and Healthy Lifestyle
Breast Cancer/Mammography Experience and Knowledge of the Ticino Screening Program
Fear of Breast Cancer
Ego involvement
Benefit for Mammography

INTERVENTION

PROMOTION FIT	PREVENTION FIT	CONTROL GROUP
(Promotion Focus Priming + Video)	(Prevention Focus Priming + Video)	(No Priming + General Health Leaflet)

POST-TEST

Intention to ask for breast cancer screening

STUDY 2

T0 - PRE-TEST

Socio-Demo variables
Health Status and Healthy Lifestyle
Breast Cancer/Mammography Experience and Knowledge of the Italian/Ticino Screening Program
Fear of Breast Cancer
Benefit for Mammography
Risk Perception
Intention to Ask for breast cancer screening
Regulatory Focus

INTERVENTION

T1 – Video message 1

PROMOTION FIT	PROMOTION NON-FIT	PREVENTION FIT	PREVENTION UN-FIT	CONTROL GROUP
(Promotion focused participants watched a promotion focused video)	(Promotion focused participants watched a prevention focused video)	(Prevention focused participants watched a prevention focused video)	(Prevention focused participants watcher a promotion focused video)	(half prevention half promotion focused participants watched a video without focus reference)

T2 – Video message 2

PROMOTION FIT	PROMOTION NON-FIT	PREVENTION FIT	PREVENTION UN-FIT	CONTROL GROUP
(Promotion focused participants watched a promotion focused video)	(Promotion focused participants watched a prevention focused video)	(Prevention focused participants watched a prevention focused video)	(Prevention focused participants watcher a promotion focused video)	(half prevention half promotion focused participants watched a video without focus reference)

POST-TEST

Intention to ask for breast cancer screening

Table 3: voice-text of the video messages and control leaflet for Study 1.

<table>
<tr><th colspan="3">STUDY 1</th></tr>
<tr><th>Promotion Fit video-message</th><th>Prevention Fit video-message</th><th>Control Leaflet</th></tr>
<tr><td colspan="2">The mammography screening is a method for the early detection of breast cancer. Using x-rays, mammograms can identify very small tumours generally longer before they are palpable. A mammogram is a method that is used early, often even without symptoms. In a screening program, experts recommend mammography from the age of 50. Here in Ticino, women aged 50 and over are invited to voluntarily undergo mammography every two years at one of the accredited Radiology Centres. For most women between the ages of 50 and 69, the benefits of screening are higher than the risks. However, nevertheless, it is essential to be adequately informed to make the best decision about mammography.</td><td rowspan="5">Healthy eating associated with an active lifestyle is a useful way for disease prevention. An adequate and balanced diet plan guarantees an optimal supply of nutrients to meet the needs of your body. A balanced diet also allows receiving substances that play a protective and/or preventive role against diseases.
This booklet - thought for people of all ages without any particular diseases - explains the scientific reasoning behind the recommendation to follow a healthy diet even in the absence of particular weight or health disorders. Anyway, in case of doubts or problems, we suggest contacting your doctor.
‘Man is what he eats.’
We eat several times a day, for a lifetime. With a life expectancy of around 80, this corresponds to approximately 85.000 meals. Considering that each meal lasts an average of thirty minutes, we spend at least five years at a table. Adding the meal preparation time, the count quickly rises to ten years or more. Nutrition is, therefore, a topic that deserves special attention. Balanced meals and a healthy lifestyle give a fundamental contribution to our daily well-being and efficiency.
«Fast-casual» instead of «fast food.»
‘Fast casual’ means eating healthy and fast, and it is not a contradiction. The ‘snack culture’ is an old idea. Those who want to have a quick meal, however, must pay attention to its composition: dairy products, fruit, vegetables, salads, and wholemeal products are the basis for a new, healthy ‘fast-casual’ menu.
The right fats for every need
The choice of fats must be made according to the intended use. Fats and oils, if heated for a long time, change with chemical reactions that can create unwanted substances harmful to health. In order to avoid such reactions, it is advisable to cook with fats that mainly contain saturated fatty acids, which are more stable at high temperatures.</td></tr>
<tr><td>To protect their health, women under the age of 50, without a medical indication or family history of cancer, are excluded from the program.</td><td>To avoid adverse effects on their health, women under the age of 50 without a medical indication or family history of cancer are excluded from the program.</td></tr>
<tr><td colspan="2">Now I would like to explain the scientific reasoning behind the recommendation to not undergo a mammogram without a medical reason. Anyway, in case of doubt or symptoms, I suggest to contact your doctor. So, you are asking why women under the age of 50 are excluded from mammography screening. Scientific research shows that for women between the ages of 50 and 69 mammography screening is the most effective method for the early detection of breast cancer and for reducing the mortality rate associated with it. In contrast, for young women between the ages of 30 and 49, the disadvantages and risks to health are greater than the benefits. This is mainly due to the fact that women before menopause have a denser breast tissue.</td></tr>
<tr><td>Given the reasons I have just presented, one should avoid undergoing a mammogram early to prevent negative consequences.</td><td>Given the reasons I have just presented, to early undergo a mammogram can lead to negative consequences.</td></tr>
<tr><td colspan="2">For example, mammography could show anomalies that, after additional diagnostic tests, could be proved to be benign. This type of error is called a false positive. If for women aged between 50 and 69 this risk is minimal, for young women is higher due to the denser breast tissue. In addition, breast cancer could not be seen by mammograms because it is too small and therefore the exam may appear normal although cancer is present. This is a false-negative result. These risks always exist, but they are higher for young women. As all medical testing, waiting for the outcome of the mammogram can generate a state of anxiety and the procedure sometimes can be perceived as painful. Radiation exposure also have health consequences. Although the exposure is minimal, for women under the age of 50 the risk is higher than the benefits of mammography. Furthermore, screening could lead to over-treatment for tumours that are benign. Over diagnosis represents approximately 1-10% of diagnosed cancers. This would expose young women to the negative effects of anti-cancer therapies, without a real need. In the absence of scientific evidence of the effectiveness of mammographic screening for young women, the inclusion of young women in the program would entail additional costs for society. These financial resources could be used to prevent other diseases.</td></tr>
</table>

**For these reasons, it is recommended that young women follow the indications for breast cancer screening.**	**For these reasons, it is recommended that young women do not ignore the indications related to breast cancer screening.**	Eating healthy is easy<br>Food provides the body with both the necessary energy and nutrients that allow it to function correctly. The diet must, therefore, provide a correct caloric intake and a sufficient amount of nutrients. No food is so complete that it contains everything the body needs. The basic rule is, therefore, to eat everything and in a varied way.<br>Food pyramid<br>The food pyramid facilitates the quantitative choice between the various food groups. It is a useful tool for all those who want to have a healthy diet. It provides clear indications on what to eat, how often, and in what quantity.<br>How do I interpret it?<br>A balanced diet requires the foods at the base of the pyramid to be consumed in higher quantities. Climbing up to the vertex, the quantities of food consumed should be limited. Nothing is forbidden, every food finds its place in a balanced diet, but the recommended quantities will depend on its location in the pyramid.<br><br>Give food the importance that deserves, eating healthier. Your health and well-being will be better!

Note: in bold are shown the specific parts of the promotion focus and prevention focus versions. The Videos created for Study 1 can be retrieved from https://youtu.be/mperSG5_9yQ and https://youtu.be/KnhRUnDoSV0. Both videos last 3:28 minutes. The videos created for Study 2 can be retrieved from https://youtu.be/btM3HrvYDlQ, https://youtu.be/BZPjFPUQuvw, https://youtu.be/-lXzGpcmzD4, https://youtu.be/jRi8Y-sZvSc. A translation of the Italian voice has been provided in Table B1 and B2

Table 4. Descriptive statistics of the pre- and post-test variables with frequencies (% frequencies between brackets) or means (standard deviations between brackets).

	STUDY 1			STUDY 2				
	**Promotion Fit**	**Prevention Fit**	**Control Group**	**Promotion Fit**	**Promotion Unfit**	**Prevention Fit**	**Prevention Unfit**	**Control Group**
**PRE-TEST VARIABLES**								
General Health Status	3.88 (.77)	3.77 (.87)	3.7 (.87)	3.79 (.79)	3.63 (.67)	3.66 (.76)	3.70 (.77)	3.76 (.69)
Physical Activity	2.45 (1.85)	2.47 (1.69)	2.43 (1.92)	.78 (.42)	.81 (.39)	.76 (.46)	.72 (.45)	.86 (.35)
Smoking Habits	1.86 (4.85)	1.99 (4.99)	3.32 (6.42)	3.53 (5.4)	3.12 (4.66)	4.93 (5.59)	3.19 (5.15)	7.22 (5.4)
Alcohol Consumption	1.92 (2.79)	1.71 (2.27)	1.42 (2.14)	3 (2.26)	2.66 (2.15)	3.27 (4.13)	2.67 (3.54)	3.1 (4)
Fear of Breast Cancer	3.4 (.85)	3.4 (.81)	3.4 (1)	3.75 (.95)	3.59 (.91)	3.79 (.95)	3.83 (.93)	3.68 (1)
Ego-Involvement	5.9 (1.1)	5.8 (1.27)	5.9 (1.3)	-	-	-	-	-
Benefit for Mammography	3.9 (.62)	3.8 (.62)	4 (.74)	4.1 (.75)	4.12 (.73)	4.16 (.68)	4.16 (.65)	3.94 (.78)
Intention to ask for bc screening	-	-	-	3.35 (1.33)	3.35 (1.29)	3.44 (1.22)	3.31 (1.40)	3.45 (1.41)
Diet								
No	46 (38%)	49 (37%)	39 (36%)	24 (41%)	30 (53%)	29 (39%)	27 (37%)	13 (45%)
Yes	76 (62%)	81 (62%)	69 (64%)	34 (59%)	27 (47%)	45 (61%)	47 (63%)	16 (55%)
BC Among Relatives								
No	117 (96%)	117 (90%)	89 (82%)	52 (90%)	48 (84%)	65 (88%)	67 (90%)	28 (97%)
Yes (Mother)	4 (3%)	8 (6%)	17 (16%)	5 (9%)	6 (11%)	7 (10%)	7 (10%)	1 (3%)
Do not know	1 (1%)	5 (4%)	2 (2%)	1 (1%)	3 (5%)	2 (2%)	-	-

Mammography								
No	100 (82%)	100 (77%)	72 (67%)	40 (69%)	38 (67%)	50 (68%)	50 (68%)	23 (79%)
Yes	22 (18%)	30 (23%)	36 (33%)	18 (31%)	19 (33%)	24 (32%)	24 (32%)	6 (21%)
Biopsy								
No	17 (77%)	27 (90%)	26 (72%)	57 (98%)	55 (97%)	67 (91%)	71 (96%)	29 (100%)
Yes	5 (23%)	3 (10%)	10 (28%)	1 (2%)	2 (3%)	7 (9%)	3 (4%)	-
Knowledge of bc								
Screening Program	76 (62%)	69 (53%)	64 (59%)	21 (36%)	23 (40%)	26 (35%)	27 (37%)	7 (24%)
No	46 (38%)	61 (47%)	44 (41%)	37 (64%)	34 (60%)	48 (65%)	47 (63%)	22 (76%)
Yes								
Knowledge of the age thresholds for bc Screening Program								
Do not know	16 (35%)	24 (39%)	13 (30%)	21 (36%)	32 (56%)	26 (35%)	27 (37%)	7 (24%)
Wrong	22 (48%)	28 (46%)	30 (68%)	29 (50%)	18 (32%)	34 (46%)	37 (50%)	18 (62%)
Correct	8 (17%)	9 (15%)	1 (2%)	8 (14%)	7 (12%)	14 (19%)	10 (13%)	14 (14%)
**POST-TEST VARIABLES**								
Intention to ask for bc screening	2.20 (1.05)	2.26 (1.06)	3.36 (1.33)	3.02 (1.61)	2.89 (1.48)	3.17 (1.48)	3 (1.54)	2.78 (1.49)

Note: bc = breast cancer

Table 5: voice-text of the video messages and control leaflet for Study 2.

	STUDY 2		
**VIDEO 1**	For the early detection of breast cancer, experts recommend mammography to women aged 50 and over. Mammography is the most effective medical examination for the early detection of breast cancer. It consists of an X-ray exposure that allows you to identify even very small tumours before they are palpable or recognizable. Women over the age of 50 are invited to undergo a mammogram every 2 years at an accredited radiology centre. For women between 50 and 69, the benefits of the exam outweigh the risks. And before the age of 50?		
	**To respect their health, women under 50 are excluded from the breast cancer screening program. (PROMOTION FOCUS)**	**To avoid adverse health effects, women under 50 are excluded from the breast cancer screening program. (PREVENTION FOCUS)**	**Women under 50 are excluded from the breast cancer screening program. (CONTROL GROUP)**
	It is a medical recommendation: before the age of 50, the risks of the examination are higher than the benefits.		
	**In the absence of proven risk, to take care of your health, doctors advise not to undergo a mammogram before the age of 50. (PROMOTION FOCUS)**	**In the absence of proven risk, to avoid adverse effects for your health, doctors advise not to undergo a mammogram before the age of 50. (PREVENTION FOCUS)**	**In the absence of proven risk, doctors advise not to undergo a mammogram before the age of 50. (CONTROL GROUP)**
**VIDEO 2**	Mammography is a breast test that allows you to detect even many small tumours. Over 50 years, it is done every two years. Women under the age of 50 are excluded from the breast cancer screening program, except in case of genetic predisposition or family history of breast cancer. What are the reasons for this decision?		
	**The observance of the age threshold determines a decreasing of the probability of false positives: breast anomalies that are benign form. (PROMOTION FOCUS)**	**The observance of the age threshold allows avoiding the negative consequences caused by false positives: breast abnormalities that are benign form. (PREVENTION FOCUS)**	**In young women, false positives are highly likely. False-positive are breast anomalies that are benign form. (CONTROL GROUP)**
	The risk of false positives in young women is higher because the breast tissue is denser.		
	**It is advised not to make mammograms before the age of 50 in order not to expose themselves to anti-cancer treatments not recommended as they are often directed to benign anomalies. (PROMOTION FOCUS)**	**It is advisable not to make mammograms before the age of 50 to avoid exposure to non-recommended anti-cancer treatments as they are often directed to benign anomalies. (PREVENTION FOCUS)**	**The breast cancer screening could lead to an exposition of non-recommended anti-cancer treatments, as they are often directed to benign anomalies. (CONTROL GROUP)**
	Breast cancer is much rarer in women under the age of 50.		
	**Excluding younger women from screening allows them to be protected from unnecessary radiation exposure. Furthermore, this choice promotes psychological well-being against stress and anxiety. For these reasons, mammographic screening involves only women over 50 years. If you are under 50 and want to take care of your health, we recommend that you respect the age threshold. (PROMOTION FOCUS)**	**Excluding younger women from screening allows you to avoid unnecessary radiation exposure. Furthermore, this choice avoids psychological discomforts, such as stress and anxiety. For these reasons, mammographic screening involves only women over 50 years. If you are under 50 years old and want to avoid negative consequences for your health, we recommend that you respect the age threshold. (PREVENTION FOCUS)**	**Radiation exposure poses health risks. In the case of mammography, the exposure is minimal, but for women, under the age of 50, the risk is higher than the benefits. Furthermore, as any other medical procedure, waiting for the outcome can generate anxiety and stress. For these reasons, mammographic screening involves only women over 50 years. If you are under 50 years old, we recommend that you respect the age threshold. (CONTROL GROUP)**
	Conscious prevention it is worth it!		

Note: in bold are shown the specific parts of the promotion focus, prevention focus, and control versions.

STROBE 2007 (v4) Statement—Checklist of items that should be included in reports of *cross-sectional studies*

Section/Topic	Item #	Recommendation	Reported on page #
**Title and abstract**	1	(*a*) Indicate the study's design with a commonly used term in the title or the abstract	#1; #2
		(*b*) Provide in the abstract an informative and balanced summary of what was done and what was found	#2
**Introduction**			
Background/rationale	2	Explain the scientific background and rationale for the investigation being reported	#4-6
Objectives	3	State specific objectives, including any prespecified hypotheses	#6
**Methods**			
Study design	4	Present key elements of study design early in the paper	#7; #11
Setting	5	Describe the setting, locations, and relevant dates, including periods of recruitment, exposure, follow-up, and data collection	#7-8; #12
Participants	6	(*a*) Give the eligibility criteria, and the sources and methods of selection of participants	#7-8; #12
Variables	7	Clearly define all outcomes, exposures, predictors, potential confounders, and effect modifiers. Give diagnostic criteria, if applicable	#8-9; #12
Data sources/ measurement	8*	For each variable of interest, give sources of data and details of methods of assessment (measurement). Describe comparability of assessment methods if there is more than one group	#8-9; #12
Bias	9	Describe any efforts to address potential sources of bias	#8-10; #12-14
Study size	10	Explain how the study size was arrived at	#6; #10-11
Quantitative variables	11	Explain how quantitative variables were handled in the analyses. If applicable, describe which groupings were chosen and why	#9; #13
Statistical methods	12	(*a*) Describe all statistical methods, including those used to control for confounding	#9-10; #13-14
		(*b*) Describe any methods used to examine subgroups and interactions	#9-10; #13-14
		(*c*) Explain how missing data were addressed	#9; #13
		(*d*) If applicable, describe analytical methods taking account of sampling strategy	Not applicable
		(*e*) Describe any sensitivity analyses	Not applicable
**Results**			

For peer review only

Participants	13*	(a) Report numbers of individuals at each stage of study—eg numbers potentially eligible, examined for eligibility, confirmed eligible, included in the study, completing follow-up, and analysed	#6-7; #10-11
		(b) Give reasons for non-participation at each stage	#6-7; #10-11
		(c) Consider use of a flow diagram	Not applicable
Descriptive data	14*	(a) Give characteristics of study participants (eg demographic, clinical, social) and information on exposures and potential confounders	#6-7; #10-11
		(b) Indicate number of participants with missing data for each variable of interest	#9; #12
Outcome data	15*	Report numbers of outcome events or summary measures	#9
Main results	16	(*a*) Give unadjusted estimates and, if applicable, confounder-adjusted estimates and their precision (eg, 95% confidence interval). Make clear which confounders were adjusted for and why they were included	#13-14
		(*b*) Report category boundaries when continuous variables were categorized	Not applicable
		(*c*) If relevant, consider translating estimates of relative risk into absolute risk for a meaningful time period	Not applicable
Other analyses	17	Report other analyses done—eg analyses of subgroups and interactions, and sensitivity analyses	Not applicable
**Discussion**			
Key results	18	Summarise key results with reference to study objectives	#14
Limitations	19	Discuss limitations of the study, taking into account sources of potential bias or imprecision. Discuss both direction and magnitude of any potential bias	#10; #14-17
Interpretation	20	Give a cautious overall interpretation of results considering objectives, limitations, multiplicity of analyses, results from similar studies, and other relevant evidence	#14-17
Generalisability	21	Discuss the generalisability (external validity) of the study results	#14-17
**Other information**			
Funding	22	Give the source of funding and the role of the funders for the present study and, if applicable, for the original study on which the present article is based	#17

*Give information separately for cases and controls in case-control studies and, if applicable, for exposed and unexposed groups in cohort and cross-sectional studies.

Note: An Explanation and Elaboration article discusses each checklist item and gives methodological background and published examples of transparent reporting. The STROBE checklist is best used in conjunction with this article (freely available on the Web sites of PLoS Medicine at http://www.plosmedicine.org/, Annals of Internal Medicine at http://www.annals.org/, and Epidemiology at http://www.epidem.com/). Information on the STROBE Initiative is available at www.strobe-statement.org.

For peer review only

BMJ Open

An Application of the Theory of Regulatory Fit to Promote Adherence to Evidence-Based Breast Cancer Screening Recommendations: Experimental vs. Longitudinal Evidence

Journal:	*BMJ Open*
Manuscript ID	bmjopen-2020-037748.R1
Article Type:	Original research
Date Submitted by the Author:	30-Jun-2020
Complete List of Authors:	Petrocchi, Serena; Università della Svizzera Italiana,<br>Ludolph, Ramona; Università della Svizzera Italiana<br>Labrie, Nanon ; Vrije Universiteit Amsterdam, Athena Institute, Faculty of Beta Sciences<br>Schulz, Peter; Universita della Svizzera Italiana, Institute of Communication & Health
**Primary Subject Heading**:	Communication
Secondary Subject Heading:	Health policy
Keywords:	PUBLIC HEALTH, Breast tumours < ONCOLOGY, PREVENTIVE MEDICINE

SCHOLARONE™
Manuscripts

I, the Submitting Author has the right to grant and does grant on behalf of all authors of the Work (as defined in the below author licence), an exclusive licence and/or a non-exclusive licence for contributions from authors who are: i) UK Crown employees; ii) where BMJ has agreed a CC-BY licence shall apply, and/or iii) in accordance with the terms applicable for US Federal Government officers or employees acting as part of their official duties; on a worldwide, perpetual, irrevocable, royalty-free basis to BMJ Publishing Group Ltd ("BMJ") its licensees and where the relevant Journal is co-owned by BMJ to the co-owners of the Journal, to publish the Work in this journal and any other BMJ products and to exploit all rights, as set out in our licence.

The Submitting Author accepts and understands that any supply made under these terms is made by BMJ to the Submitting Author unless you are acting as an employee on behalf of your employer or a postgraduate student of an affiliated institution which is paying any applicable article publishing charge ("APC") for Open Access articles. Where the Submitting Author wishes to make the Work available on an Open Access basis (and intends to pay the relevant APC), the terms of reuse of such Open Access shall be governed by a Creative Commons licence – details of these licences and which Creative Commons licence will apply to this Work are set out in our licence referred to above.

Other than as permitted in any relevant BMJ Author's Self Archiving Policies, I confirm this Work has not been accepted for publication elsewhere, is not being considered for publication elsewhere and does not duplicate material already published. I confirm all authors consent to publication of this Work and authorise the granting of this licence.

For peer review only

An Application of the Theory of Regulatory Fit to Promote Adherence to Evidence-Based Breast Cancer Screening Recommendations: Experimental vs. Longitudinal Evidence

Serena Petrocchi, Ramona Ludolph Ramona, Nanon H.M. Labrie, Peter J. Schulz

Corresponding author

Dr Serena Petrocchi

Institute of Communication and Health

Università della Svizzera italiana

Via Buffi 13

6900 Lugano

Switzerland

T: 0041 (0)58 666 4821

Fax: 0041 (0)58 666 4000

E: serena.petrocchi@usi.ch

Dr Ramona Ludolph:

Institute of Communication & Health

Università della Svizzera italiana

Via Buffi 13

6900 Lugano

Switzerland

Dr Nanon H.M. Labrie

Athena Institute

Vrije Universiteit Amsterdam

De Boelelaan 1105, 1081 HV Amsterdam

The Netherlands: n.h.m.labrie@vu.nl

Prof. Peter J. Schulz
Institute of Communication & Health
Università della Svizzera italiana
Via Buffi 13
6900 Lugano
Switzerland: peter.schulz@usi.ch

Keywords: breast cancer screening; theory of regulatory fit; breast cancer prevention; mammography

Word count: 4168 words

For peer review only - http://bmjopen.bmj.com/site/about/guidelines.xhtml

Abstract

Objectives. To prevent overtreatment caused by false positive results and overuse, it is advisable to reduce the demand for mammography screening without medical indication among women who are not yet eligible for inclusion in systematic screening programmes,.. Following the principles of regulatory fit theory, two studies investigated whether messages explaining the risks and benefits of mammography screening for those not yet eligible, are perceived as more persuasive when the elements they highlight match a woman's goal-pursuit orientation. One's goal-pursuit orientation can be promotion focused, oriented towards achieving gains, or prevention focused, oriented towards avoiding losses. **Design**. Study 1 was an experimental study in which women's goal-pursuit orientation was experimentally induced and then they were exposed to a matching video message about mammography screening. A control group received a neutral stimulus. Study 2 introduced a longitudinal component to Study 1, adding a condition in which the messages did not match with the group's goal-pursuit orientation. This time, participants' natural goal-pursuit orientation was measured through a validated questionnaire. **Participants**. 360 women participated in Study 1 and 292 in Study 2. Participants' age ranged from 30 to 45 years, and had no history of breast cancer or known BRCA 1/2 mutation. **Results**. In Study 1, a match between participants' goal-pursuit strategy and message content decreased the intention to seek mammography without a medical indication. Study 2, however, did not show such an effect. Fear of breast cancer and risk perception are significantly related to intention to seek mammography. **Conclusions**. Public health researchers should consider reducing the impact of negative emotions (i.e. fear of breast cancer) and risk perception when promoting adherence to evidence-based breast cancer screening recommendations.

For peer review only - http://bmjopen.bmj.com/site/about/guidelines.xhtml

Keywords: breast cancer screening; theory of regulatory fit; breast cancer prevention; mammography

Strengths and Limitations of this Study

- An experimental study (Study 1) and an experimental study with a longitudinal component (Study 2) were implemented applying principles from the theory of regulatory fit.
- An individual's goal-pursuit orientation was induced in Study 1 through a priming technique, and measured through a validated questionnaire in Study 2.
- Messages were tailored to create a match (or not) between message content and the individual's goal-pursuit orientation.
- Limitations of the studies included dropout rates (Study 2) and selection bias (due to cancer fear).

Breast cancer is one of the most common forms of cancer in women worldwide and the principal cause of cancer-related death in the female population,[1]. To promote early diagnosis, many EU countries have introduced systematic breast cancer screening programs,[2]. However, the age threshold to start inviting women to screening is in dispute,[3-5]. The balance between the benefits (i.e., reducing breast cancer mortality) and the harm associated with mammography (i.e. x-ray exposure, over diagnosis and false positive results; see,[4-8] is uncertain. Technologies for breast cancer screening have been constantly evolving, affecting evidence quality and suggested recommendations,[9]. For these reasons recommended age for starting screening have varied from 40,[10], to 45,[11,12], to 50 years,[13,14].

There has been a vast amount of research investigating the intentions of women to adhere to screening guidelines and encouraging women with characteristics that match with the national guidelines to attend systematic screening,[15-17]. However, many women below the established age threshold seek and receive mammography screenings without medical reasons in the U.S.,[18,19], Switzerland,[20,21], Germany,[22], and The Netherlands,[22]. Studies show that women tend to overestimate the mortality reduction determined by breast cancer screening,[23,24] and that they have unrealistic expectations regarding screening as reducing the risk of breast cancer,[25]. Moreover, social pressure in favour of breast cancer screening may stimulate a sense of moral obligation to participate,[26,27], even among young women.

Given the above-mentioned considerations, women under the age threshold for systematic breast cancer screening may consider the recommendation to avoid screening as counterintuitive, although scientifically supported, because of social pressure and the belief that cancer screening can save lives. The present research aimed to promote adherence to evidence-based recommendations on breast cancer

For peer review only - http://bmjopen.bmj.com/site/about/guidelines.xhtml

screening among young women by activating a motivation system, such as regulatory orientation,[28].

Theory of Regulatory Fit

According to a popular psychological theory proposed by [29], people show one of two regulatory orientations, which determines how they pursue their goals. They either show a promotion-focused orientation, meaning they eagerly strive towards the realization of desired outcomes, or they show a prevention-focused orientation, emphasizing the prevention of errors and losses and making them safety-driven, [29,30]. While every individual has a natural tendency to lean more towards one orientation than the other, thus making it a measurable trait,[31], the regulatory orientation can also be experimentally induced,[28,29,32].

If individuals adopt a behaviour or processes a message highlighting goal-pursuit strategies that match their regulatory orientation, they experience a phenomenon called “regulatory fit”,[28]. For example, if a person with a promotion orientation reads a message highlighting strategies to achieve gains, a fit condition occurs. The same applies to someone with a prevention orientation processing a message emphasizing strategies to avoid losses. Such a fit or match causes an “it just feels right” perception, increasing the perceived value of the behaviour[33].

Regulatory fit has been consistently found to influence outcomes such as evaluation, behaviour and behavioural intention,[34]. Some authors [33] showed that this “it-just-feels-right” experience is also transferred to the context of persuasion, with positive effect of regulatory fit on the perceived persuasiveness of a message. A study by [35] in the context of tobacco use prevention among adolescents is in line with this finding. The effects of regulatory fit have also been extensively studied in the context of disease prevention and health promotion,[35,36]. In particular, some authors [37] applied the principles of regulatory fit to inform people about the benefits of regular

For peer review only - http://bmjopen.bmj.com/site/about/guidelines.xhtml

cancer screenings. A systematic review,[38] finds that the use of the principle of regulatory fit has the potential to increase the effectiveness of health communication across a range of health contexts and outcomes, making it a promising tool for tackling the problem of unwarranted demand for mammography screening without medical reason according to the local guidelines.

The purpose of the present research was to test whether health messages framed to correspond with a woman's regulatory orientation are effective in *reducing* the intention to ask for breast cancer screening in non-at risk women under the age of 45, according to the local mammography screening guidelines. The following hypotheses have been tested:

HP1: a fit between the message frame and the regulatory orientation would lead to an immediate reduction of the intention to ask for breast cancer screening, in non-at risk women under the age threshold indicated by the local guidelines. This hypothesis was tested in experimental Study 1.

HP2: a fit between the message frame and the regulatory orientation would lead to a reduction of the intention to ask for breast cancer screening, stable over time. This hypothesis was tested in the longitudinal experimental Study 2.

Study 1

Methods

Participants

An a priori power analysis applying G*Power 3.1.9.2 ,[39] estimated a sample of 249 participants ($\alpha = .05$, $d = .95$, $\eta^2 = 0.05$; see,[38]). Participants living in the Italian-speaking, Swiss canton of Ticino completed an online survey from June to September 2016. The research was repeatedly advertised on the Facebook page of the University. Exclusion criteria were: a personal history of breast cancer, BRCA mutations, insufficient fluency in Italian. The survey required women to answer each question

before progressing to the next screen; as such there were no missing data. Participants received a 10 CHF supermarket voucher for their participation in Study 1. Before starting the questionnaire, participants completed a written informed consent by clicking on the corresponding button (i.e. “yes, I want to participate”; “no, I do not want to participate”).

Five hundred women from 30 to 45 years started the survey: 121 (16%) initiated the pre-test questionnaires but dropped out. Nineteen of the women were excluded from the final sample because they did not complete the experimental manipulation No differences emerged in the pre-test variables between those who filled in only the pre-test (N = 140) and who filled in the entire survey (N = 360). Participants were randomly assigned to prevention fit, promotion fit, and control condition (see Table 1). No differences were found between the intervention groups and the control group on socio-demographic variables.

For peer review only - http://bmjopen.bmj.com/site/about/guidelines.xhtml

Table 1. Demographics of Study 1 and Study 2.

	Study 1			Study 2				
	**Promotion Fit (N = 122)**	**Prevention Fit (N = 130)**	**Control Group (N = 108)**	**Promotion Fit (N = 58)**	**Promotion Unfit (N = 57)**	**Prevention Fit (N = 74)**	**Prevention Unfit (N = 74)**	**Control Group (N = 29)**
**Age** (range 30-45): M and (SD)	36.55 (4.42)	38.07 (4.57)	38.37 (4.79)	38.1 (4.96)	38.53 (4.7)	38.31 (4.44)	37.93 (4.41)	37.02 (4.99)
**Marital Status**								
Married	73 (59%)	77 (60%)	69 (64%)	36 (62%)	41 (72%)	55 (74%)	53 (72%)	22 (76%)
Single	38 (31%)	38 (30%)	26 (24%)	20 (35%)	12 (21%)	14 (19%)	17 (23%)	6 (21%)
Divorced/Separated/Widowed	11 (10%)	15 (10%)	13 (12%)	2 (3%)	4 (7%)	5 (7%)	4 (5%)	1 (3%)
**Educational Level**								
Elementary/Junior School	2 (2%)	2 (2%)	3 (3%)	1 (2%)	-	1 (1%)	4 (5%)	-
High School	44 (34%)	56 (46%)	58 (54%)	18 (31%)	24 (43%)	40 (54%)	28 (38%)	8 (28%)
University or Post University Degree	84 (64%)	64 (52%)	47 (43%)	39 (66%)	33 (57%)	33 (45%)	42 (57%)	21 (72%)
**Occupation**								
Employed	102 (84%)	107 (82%)	74 (69%)	48 (83%)	50 (88%)	57 (77%)	67 (91%)	29 (90%)
Homemaker	11 (9%)	14 (11%)	22 (20%)	4 (7%)	3 (5%)	7 (9%)	6 (8%)	1 (3%)
Unemployed	8 (6%)	7 (5%)	10 (9%)	4 (7%)	4 (7%)	8 (11%)	1 (1%)	2 (7%)
Student	1 (1%)	2 (2%)	2 (2%)	2 (3%)	-	2 (3%)	-	-
**Nationality**								
Swiss	97 (80%)	101 (78%)	73 (68%)	10 (17%)	15 (26%)	16 (18%)	13 (18%)	-
Italian	21 (17%)	23 (18%)	26 (24%)	47 (81%)	40 (70%)	53 (71%)	58 (78%)	27 (93%)
Other	4 (3%)	6 (4%)	9 (8%)	1 (2%)	2 (4%)	5 (7%)	3 (4%)	4 (7%)
**Mother Tongue**								
Italian	117 (96%)	122 (94%)	94 (87%)	54 (93%)	54 (93%)	68 (92%)	71 (96%)	27 (93%)
Other	5 (4%)	8 (6%)	14 (13%)	4 (7%)	4 (7%)	6 (8%)	3 (4%)	2 (7%)

Process, Measures and Data Collection

A pre-post-test design with two experimental conditions and a control group was applied (see Figure 1 for full details).

[Insert Figure 1 here]

At pre-test, the survey included measures of health status and health behaviours, a set of questions on past diagnosis of breast cancer, mammography, biopsy and knowledge of the Ticino screening program. Women were rated on their fear of breast cancer, level of involvement in breast cancer and confidence in the benefit of mammography (see online supplementary material).

Regulatory priming manipulation was induced following,[40] procedure (online supplementary material). Participants were then randomly assigned into a promotion fit, prevention fit or control condition. In the fit conditions, immediately after priming, the participants watched a video-message highlighting goal-pursuit strategies matching with the primed focus (online supplementary material). The control group received a leaflet without any prompt for the regulatory orientation (online supplementary material). In a pilot study, 30 women assessed the survey as clear and understandable. The University's Ethical Committee approved Study 1 and Study 2.

Patient and Public Involvement

Results from previous studies involving participants from Switzerland informed the present research (see,[21]). Participants were not directly involved in the design, conduct, recruitment, reporting or dissemination of the study results. An expert panel, composed of two health communication professionals with expertise on regulatory fit theory, evaluated the message contents and the graphical aspects of the videos.

Analytic Strategy

For peer review only - http://bmjopen.bmj.com/site/about/guidelines.xhtml

Data were normalized through reverse scoring and logarithmic transformations. There were no missing data. An ANCOVA tested the main hypothesis (HP1) of the study. The fit vs. control conditions variable was inserted as independent variable. All the variables measured at the pre-test were inserted as covariates. Chi-square tests were conducted to evaluate whether the covariates might interact with the three experimental conditions in determining the intention to ask for breast cancer screening.

Results

The ANCOVA analysis revealed that women in the two experimental conditions showed less intention to ask for breast cancer screening compared to the women in the control condition. Thus, when there is a fit between individual orientation (i.e., a tendency to promote positive expected outcomes or to prevent negative outcomes for one's health) and the given message, then a persuasive effect is induced. There was no meaningful difference between the two manipulation conditions. Older women and women with higher levels of fear of breast cancer showed a greater intention to ask for breast cancer screening than younger ones and those with lower levels of fear. This evidence supports the assumption that regulatory orientation represents a motivational system able to overcome the impact of negative emotions and strengthen an individual's involvement in decision-making orientation. Descriptive data and results from the ANCOVA are displayed in Table 2.

For peer review only - http://bmjopen.bmj.com/site/about/guidelines.xhtml

Table 2. Descriptive statistics of the pre- and post-test variables with frequencies (% frequencies between brackets) or means (standard deviations between brackets), and results of the analyses.

	STUDY 1			STUDY 2				
	**Promotion Fit**	**Prevention Fit**	**Control Group**	**Promotion Fit**	**Promotion Unfit**	**Prevention Fit**	**Prevention Unfit**	**Control Group**
**PRE-TEST VARIABLES**								
General Health Status	3.88 (.77)	3.77 (.87)	3.7 (.87)	3.79 (.79)	3.63 (.67)	3.66 (.76)	3.70 (.77)	3.76 (.69)
Physical Activity	2.45 (1.85)	2.47 (1.69)	2.43 (1.92)	.78 (.42)	.81 (.39)	.76 (.46)	.72 (.45)	.86 (.35)
Smoking Habits	1.86 (4.85)	1.99 (4.99)	3.32 (6.42)	3.53 (5.4)	3.12 (4.66)	4.93 (5.59)	3.19 (5.15)	7.22 (5.4)
Alcohol Consumption	1.92 (2.79)	1.71 (2.27)	1.42 (2.14)	3 (2.26)	2.66 (2.15)	3.27 (4.13)	2.67 (3.54)	3.1 (4)
Fear of Breast Cancer	3.4 (.85)	3.4 (.81)	3.4 (1)	3.75 (.95)	3.59 (.91)	3.79 (.95)	3.83 (.93)	3.68 (1)
Ego-Involvement	5.9 (1.1)	5.8 (1.27)	5.9 (1.3)	-	-	-	-	-
Benefit for Mammography	3.9 (.62)	3.8 (.62)	4 (.74)	4.1 (.75)	4.12 (.73)	4.16 (.68)	4.16 (.65)	3.94 (.78)
Intention to ask for bc screening	-	-	-	3.35 (1.33)	3.35 (1.29)	3.44 (1.22)	3.31 (1.40)	3.45 (1.41)
Diet								
No	46 (38%)	49 (37%)	39 (36%)	24 (41%)	30 (53%)	29 (39%)	27 (37%)	13 (45%)
Yes	76 (62%)	81 (62%)	69 (64%)	34 (59%)	27 (47%)	45 (61%)	47 (63%)	16 (55%)
BC Among Relatives								
No	117 (96%)	117 (90%)	89 (82%)	52 (90%)	48 (84%)	65 (88%)	67 (90%)	28 (97%)
Yes (Mother)	4 (3%)	8 (6%)	17 (16%)	5 (9%)	6 (11%)	7 (10%)	7 (10%)	1 (3%)
Do not know	1 (1%)	5 (4%)	2 (2%)	1 (1%)	3 (5%)	2 (2%)	-	-

For peer review only - http://bmjopen.bmj.com/site/about/guidelines.xhtml

Mammography								
No	100 (82%)	100 (77%)	72 (67%)	40 (69%)	38 (67%)	50 (68%)	50 (68%)	23 (79%)
Yes	22 (18%)	30 (23%)	36 (33%)	18 (31%)	19 (33%)	24 (32%)	24 (32%)	6 (21%)
Biopsy								
No	17 (77%)	27 (90%)	26 (72%)	57 (98%)	55 (97%)	67 (91%)	71 (96%)	29 (100%)
Yes	5 (23%)	3 (10%)	10 (28%)	1 (2%)	2 (3%)	7 (9%)	3 (4%)	-
Knowledge of BC Screening Program	76 (62%)	69 (53%)	64 (59%)	21 (36%)	23 (40%)	26 (35%)	27 (37%)	7 (24%)
No	46 (38%)	61 (47%)	44 (41%)	37 (64%)	34 (60%)	48 (65%)	47 (63%)	22 (76%)
Yes								
Knowledge of the age thresholds for BC Screening Program								
Do not know	16 (35%)	24 (39%)	13 (30%)	21 (36%)	32 (56%)	26 (35%)	27 (37%)	7 (24%)
Wrong	22 (48%)	28 (46%)	30 (68%)	29 (50%)	18 (32%)	34 (46%)	37 (50%)	18 (62%)
Correct	8 (17%)	9 (15%)	1 (2%)	8 (14%)	7 (12%)	14 (19%)	10 (13%)	14 (14%)
**POST-TEST VARIABLES**								
Intention to ask for BC screening	2.20 (1.05)	2.26 (1.06)	3.36 (1.33)	3.02 (1.61)	2.89 (1.48)	3.17 (1.48)	3 (1.54)	2.78 (1.49)
Results from Ancova[a] or Repeted Measures Ancova[b]	$F^a(2, 319) = 49.57$, $p < .0001$, $\eta^2_p = .24$<br><br>Promotion fit vs. Control Condition $t(319) = -8.80$, $p < .0001$, $r = .44$<br>Prevention Fit vs. Control Condition $t(319) = -8.80$, $p < .0001$, $r = .44$<br><br>Significant covariates<br>Fear of Breast Cancer: $F(1, 319) = 6.81$, $p = .010$, partial $\eta^2_p = .02$]<br>Age: $F(1, 319) = 26.20$, $p < .0001$, partial $\eta^2_p = .08$			Within subject comparison between pre- and post- intention: $F^b (1, 267.91) = 5.10$, p = .025, partial $\eta^2 = .02$<br>Between subject comparisons among groups: $F^b (4, 284) = .43$, p > .05<br><br>Significant covariates:<br>Fear of breast cancer: t(284) = 2.76, p = .006, B = .24, partial $\eta^2 = .03$ (95% Low CI = .07, 95% High CI = .42)<br>Age, t(284) = 6.26, p < .0001, B = .11, partial $\eta^2 = .12$ (95% Low CI = .08, 95% High CI = .15),<br>Risk perception, t(284) = 2.26, p = .024, B = .37, partial $\eta^2 = .02$ (95% Low CI = .05, 95% High CI = .70),				

Note: BC = Breast Cancer

Further analyses were conducted to evaluate whether the covariates might interact with the three experimental conditions in determining the intention to ask for breast cancer screening. Analyses revealed only one association among the three groups of women and the past diagnoses of breast cancer among first degree-relatives, χ^2 (2) = 12.98, p = .002. Women in the promotion fit condition had a lower number of breast cancer diagnoses among first-degree relatives than was expected (z = -1.96), while women in the control condition had a higher number than expected (z = 2.8). The subsequent ANCOVA did not find any significant interaction between past diagnosis of breast cancer among first-degree relatives and the experimental manipulations, therefore demonstrating that the regulatory fit genuinely influences the intention.

Study 2

Methods

Participants

A priori power analysis estimated a sample size of 312. Recruitment took place from June to October 2017. The research was advertised through the Facebook page of the University and by RCSMedia Group, an Italy-based publishing group that uses participant panels. Inclusion/exclusion criteria were as for Study 1, with the addition that participants included in Study 1 could not participate in Study 2. Participants completed a written informed consent as for Study 1, and at the end received a 10 CHF/EU supermarket voucher.

973 women aged from 30 to 45 filled in the pre-test questionnaires (i.e., pre-test sample). Completed questionnaires (i.e., analytical sample) were returned by 292 women with an attrition rate of 70%. Comparisons between the pre-test sample and the analytical sample did not yield significant differences. 292 participants were randomly assigned to five conditions: promotion fit, promotion non-fit, prevention fit, prevention non-fit, and control condition (see Table 1). This time, women aged 30 to 45 living in

Ticino and Italy participated. Italian and Ticino-Swiss participants are not only comparable from a cultural and linguistic point-of-view, but also screening guidelines in Ticino and Italy are alike, inviting 50 to74-year-olds biennially for mammography screening. No differences were found among the five groups regarding socio-demographic variables or other pre-test variables.

Process, Measures and Data Collection

A pre-post-test longitudinal design was applied with four experimental conditions, two fit conditions (promotion and prevention), two non-fit conditions (promotion and prevention) and a control group (see Figure 2).

[Insert Figure 2 approx. here]

In the pre-test (T0), participants replied to the same questions as for Study 1 (see online supplemental material). In Study 2, the regulatory focus orientation was measured with a questionnaire (online supplemental material), rather than induced as in Study 1, because working with the trait regulatory focus would be more stable than a primed focus in a longitudinal design. Subsequently, participants were randomly assigned to the fit or non-fit condition or control group. Participants in the fit conditions watched two videos (at T1 and T2) emphasizing the fit concerns (online supplemental material). In the non-fit conditions, participants watched two videos (at T1 and T2) emphasizing the non-fit concerns (online supplemental material). In the control group, participants watched two videos (at T1 and T2) treating the topic of breast cancer prevention, but without any regulatory prompt (online supplemental material).

A post-test questionnaire evaluated the women's intention to ask for opportunistic screening (T3). Ten days elapsed between each experimental phase. Three women from the general population assessed the videos as comprehensible and

clear. The final survey was tested by fifteen women aged 30 to 45, who assessed it as clear and comprehensible.

Patient and Public Involvement

As for Study 1.

Analytic Strategy

Data were normalized through reverse scoring and logarithmic transformations. There were no missing data. A repeated measure ANCOVA tested the main hypothesis (HP2) of the study. The fit vs. unfit vs. control conditions variable was inserted as independent variable. All the variables measured at the pre-test were inserted as covariates.

Results

There was a general significant decrease of the intention from pre- to post-evaluation across groups, but no significant differences among them, indicating that the scores of the post-test intention among the five groups were in general the same. Among the covariates older women, greater fear of breast cancer and greater risk perception were associated with greater post-test intention compared to the opposite. Table 2 shows descriptive statistics and results from the analysis.

The intervention effect was not significant either when the two fit conditions and the two non-fit conditions were collapsed into two categories (i.e., comparison among fit condition vs. un-fit condition vs. control) as done in Study 1, even though a general decrease in the post-intention across groups was found as before. Risk perception was tested as a moderator, but the analysis was not significant.

Discussion

The application of regulatory fit in the area of health communication is beneficial across various health contexts and outcomes,[38] (Ludolph & Schulz, 2015). The scientific community recognizes an undoubtable value in studies,[15-17]

investigating the intentions of women to adhere to breast cancer screening guidelines, with the aim to encourage them to attend screening, rather than to avoid it. Anyway, there is a widespread debate on what the age threshold to start inviting women to screening should be,[3-5]. Possible beneficial effects of screening and the harm associated with it have to be balanced for informed decision-making. The most recent European Guidelines,[11] suggest that, in absence of risk conditions, women under the age of 45 should not receive breast cancer screenings regularly.

No previous studies have tested messages designed according to the assumptions of regulatory fit to influence the intention to *not* engage in disease detection screening. This would challenge the intuitive perception that breast cancer screening leads to a mortality reduction determined by breast cancer,[23,24] and the unrealistic expectations regarding screening as reducing the risk of breast cancer,[25].

The present research shows inconsistent results. Study 1 confirmed the hypothesized effect of the intervention on the intention to seek mammography before the age of 45, with a reduction of the intention when a fit between the message frame and the individual's regulatory focus occurred. Longitudinal results from Study 2 demonstrated that this effect was not significant over one month, although a general decrease of the intention across groups was observed. Even though further evidence is needed to confirm our results, it still seems that the 'just-feels-right' experience appears to be insufficient to convince non-at risk women under the age threshold to avoid systematic breast cancer screening in the long run.

Our results could genuinely reflect the fact that the regulatory fit is not sufficient to induce a long-term decrease in women's intentions or could be an artefact of the research itself. Study 1 and Study 2 applied two different ways to evoke a regulatory orientation. Study 1 primed the individuals' regulatory orientation, whereas Study 2 measured it with a questionnaire to overcome a limitation of Study 1 and explore a

different aspect of the theory. One could argue that the different ways to induce vs. measure the regulatory orientation could have influenced the persuasiveness of the message and so its effectiveness. However, researchers of regulatory orientation suggest that there is no difference between the two procedures,[32]. Therefore, we could exclude that the two methods have had a differential impact on post-test intention. Possible differences in the cultural milieu of Italian-speaking Swiss and Italian participants might make the population primed to receive or primed to ignore the intervention. However, to the best of our knowledge there is no study comparing different cultural environments in the propensity to be primed or not.

The relatively small sample size and the recruitment strategies could have influenced the power of the analyses, the sample composition and, ultimately, the significance of the results. However, there is no such concern in Study 2 since the effect due to the intervention was not significant either when the two fit conditions and the two non-fit conditions were collapsed into two categories.

Finally, a variable might have moderated the association between intervention and intention. As [41] demonstrated, individuals' consideration of future consequences of a particular behaviour influences the effectiveness of framing techniques in predicting risk perceptions, attitudes and behavioural intentions regarding health-related advertisements. In our research, the risk perception was tested as a moderator variable, but the analyses yielded no significant results.

Fear of breast cancer, age, and risk perception (only in Study 2) were significantly related to women's intentions. The predicting role of age is not surprising because, approaching the age of 50, women are invited to undertake regular mammography screening in Ticino and in Italy. Risk perception and fear of breast cancer are the most sensitive variables. Breast cancer naturally evokes negative emotions,[21,42-44]. Moreover, the benefits of mammography screening often seem to

be overestimated,[23,25]. Therefore, it is challenging to develop effective health messages promoting the adherence to breast cancer screening guidelines for young women based on factual information. As messages based on the principles of regulatory fit take the motivational orientations of recipients into account, they go beyond the effectiveness of purely providing information. Here, messages building on the theory of regulatory fit did not seem to offer a new way to overcome the 'emotional barrier' generated by the fear of breast cancer. However, Study 2 demonstrated a general 'pedagogical effect' deriving from talking about the topic of breast cancer screening without evoking a boomerang effect (i.e. an increase of intention instead of a decrease).

The present research has several limitations. We experienced high dropout rates, especially in Study 2. The high dropout rates may be related to the topic of breast cancer itself or the fear associated with it. One could assume that women with a low level of fear of breast cancer may have decided not to take part in our research, and this may have created a selection bias that could affect the generalizability of the results. A second limitation concerns the fact that we measured the intention to ask for breast cancer screening, not the actual behaviour. Although according to many theories in the field of health promotion (e.g. Health Belief Model), the intention is a valid predictor of the actual behaviour, it would be beneficial if future research followed women until the moment they actually have a mammography.

In conclusion, it seems that by framing health messages that conform to a promotion or prevention focus, a decrease in the intention to ask for merely preventive opportunistic mammography screening is observed; but this takes place only immediately after message exposure. The influence decreases over time, and the messages lose their predictive effects after one month. This may be because breast cancer fear/opinions are very deeply ingrained in women and one/two messages cannot

change that. Accordingly, possibly results from Study 1 are valid, but repeated exposure to more than one regulatory fit message is needed to change viewpoints in the long term.

Even though our results only partially confirmed our hypothesis, there are substantial implications for future research. The results demonstrate that fear of breast cancer and risk perception are the main challenges to face in order to promote adherence to evidence-based recommendations on breast cancer screening. Public health researchers must investigate what factors may increase the effectiveness of health information. According to our evidence, future research may consider understanding how to reduce the impact of negative emotions rather than try to overcome their effect. For example, a research [45] found that humour in health messages reduces the anxiety associated with performing cancer screening. Humour may be implemented in health messages aimed to promote evidence-based breast cancer screening recommendations. Reducing the number of unnecessary breast cancer screenings would thus allow the prevention of avoidable false positive and false negative diagnoses and unjustifiable mental and physical suffering for women. In the long term, this would also enable policy-makers and health professionals to allocate scarce resources for disease prevention, detection and treatment in a more effective way.

Funding Statement

This work was supported by the Swiss National Science Foundation [grant number FNS 100019-153131/1].

Competing interests statement

None declared.

Author contributions

SP and RL drafted the first version of the manuscript. All authors contributed to writing and critically revising it and approved its final version. PJS acquired funding. NHML, PJS and RL designed Study 1 and prepared the materials. PJS, SP and RL designed Study 2 and prepared the materials. SP and RL collected data for Study 1. SP collected data for Study 2. SP performed the analyses for Study 1 and Study 2.

Acknowledge

We are very grateful to Prof. John Hodgson for his thoughtful revisions.

Data Availability Statement

The data that support the findings of this study are available from the corresponding author, [SP], upon reasonable request.

References

[1] Torre, L.A., Islami, F., Siegel, R.L., Ward, E.M., Jemal, A., (2017). Global cancer in women: burden and trends. *Cancer Epidemiology, biomarkers & Prevention. CEBP Focus: Global Cancer in Women, 26(4),* 444-457.

[2] Altobelli, E., & Lattanzi, A. (2014). Breast cancer in European Union: An update of screening programs as of March 2014 (Review). *International Journal of Oncology, 45(5),* 1785–1792.

[3]Bucchi, L., Ravaioli, A., Baldacchini, F., Giuliani, O., Mancini, S., Vattiato, R., ... & Di Felice, E. (2019). Annual mammography at age 45–49 years and biennial

mammography at age 50–69 years: comparing performance measures in an organised screening setting. *European radiology*, *29*(10), 5517-5527.

[4]Gøtzsche, P. C., & Jørgensen, K. J. (2013). Screening for breast cancer with mammography. *Cochrane Database of Systematic Reviews*, *6*, CD001877.

[5] van den Ende, C., Oordt-Speets, A. M., Vroling, H., & van Agt, H. M. (2017). Benefits and harms of breast cancer screening with mammography in women aged 40–49 years: A systematic review. *International Journal of Cancer*, *141*(7), 1295-1306.

[6] Armstrong, K., Moye, E., Williams, S., Berlin, J. A., & Reynolds, E. E. (2007). Screening mammography in women 40 to 49 years of age: a systematic review for the American College of Physicians. *Annals of Internal Medicine, 146,* 516–26.

[7]Barratt, A. L., Howard, K., Irwig, L., Salked, G., & Houssami, N. (2005). Model of outcomes of screening mammography: information to support informed choices. *British Medical Journal, 330,* 936–938.

[8]Gøtzsche, P. C., & Nielsen, M. (2006). Screening for breast cancer with mammography. *Cochrane Database of Systematic Review*, *4,* CD001877.

[9]Carter, S. M., Williams, J., Parker, L., Pickles, K., Jacklyn, G., Rychetnik, L., & Barratt, A. (2015). Screening for cervical, prostate, and breast cancer: interpreting the evidence. *American Journal of Preventive Medicine*, *49*(2), 274-285. [10]Mainiero, M. B., Lourenco, A., Mahoney, M. C., Newell, M. S., Bailey, L., Barke, L. D., ... & Jokich, P. M. (2016). ACR appropriateness criteria breast cancer screening. *Journal of the American College of Radiology*, 13(11), R45-R49.

[11]European Commission Initiative on Breast Cancer (ECIBC) Guideline (2018). European guidelines on breast cancer screening and diagnosis. Retrieved from: https://healthcare-quality.jrc.ec.europa.eu/european-breast-cancer-guidelines/screening-ages-and-frequencies#recs-group-40-44

[11]Oeffinger KC, Fontham ET, Etzioni R et al (2015) Breast cancer screening for women at average risk: 2015 guideline update from the American Cancer Society. *JAMA* 314:1599–1614

[13]Lauby-Secretan, B., Scoccianti, C., Loomis, D., Benbrahim-Tallaa, L., Bouvard, V., Bianchini, F., & Straif, K. (2015). Breast-cancer screening—viewpoint of the IARC Working Group. *New England journal of medicine*, 372(24), 2353-2358.

[14]Siu AL (2016) Screening for breast cancer: U.S. Preventive Services Task Force recommendation statement. *Annals Internal Medicine* 164:279–296

[15]Ahmad, F., Cameron, J. I., & Stewart, D. E. (2005). A tailored intervention to promote breast cancer screening among South Asian immigrant women. *Social science & medicine*, 60(3), 575-586.

[16]Azaiza, F., & Cohen, M. (2006). Health beliefs and rates of breast cancer screening among Arab women. *Journal of Women's Health*, 15(5), 520-530.

[17]Payán, D. D., Maggard-Gibbons, M., Flórez, K. R., Mejía, N., Hemmelgarn, M., Kanouse, D., ... & Lara, M. (2020). Taking Care of Yourself and Your Risk for Breast Cancer (CUIDARSE): A Randomized Controlled Trial of a Health Communication Intervention for Latinas. *Health Education & Behavior*, 1090198120920529.

[18]Block, L. D., Jarlenski, M. P., Wu, A. W., & Bennett, W. L. (2013). Mammography use among women ages 40-49 after the 2009. US Preventive Services Task Force recommendation. *Journal of General Internal Medicine, 28(11),* 1447-1453.

[19]Kapp, J. M., Ryerson, A. B., Coughlin, S. S., & Thompson, T. D. (2009). Racial and ethnic differences in mammography use among US women younger than age 40. Breast Cancer Research and Treatment, 113(2), 327-337.

[20]Glaus, A., Fäh, B., Hornung, R., Senn, H., & Stiefel, F. (2004). Das Brustkrebs-Präventionsverhalten in der Schweiz: aus der Perspektive von Frauen aus drei

Sprachregionen der Schweiz. [Breast cancer prevention behaviour: a perspective of women from three language regions of Switzerland]. Pflege, 17(6), 385-394.

[21]Labrie, N. H., Ludolph, R. A., & Schulz, P. J. (2020). Mammography perceptions and practices among women aged 30–49: The role of screening programme availability and cultural affiliation. Patient Education and Counseling, 103(2), 369-375.

[22]Klug, S. J., Hetzer, M., & Blettner, M. (2005). Screening for breast and cervical cancer in a large German city: participation, motivation and knowledge of risk factors. The European Journal of Public Health, 15(1), 70-77.

[22]Statistics Netherlands (2015). CBS: Ruim de helft van de vrouwen laat uitstrijkje maken. [More than half of all women get a pap-smear]. Retrieved from: http://www.cbs.nl/nl-NL/menu/themas/gezondheid-welzijn/publicaties/artikelen/archief/2015/ruim-de-helft-van-de-vrouwen-laat-uitstrijkje-maken.htm

[23]Chamot, E., & Perneger, T. V. (2001). Misconceptions about efficacy of mammography screening: a public health dilemma. Journal of Epidemiology & Community Health, 55, 799-803.

[24]Gigerenzer, G., Mata, J., & Frank, R. (2009). Public knowledge of benefits of breast and prostate cancer screening in Europe. Journal of the National Cancer Institute, 101(17), 1216-1220.

[25]Domenighetti, G., D'avanzo, B., Egger, M., Berrino, F., Perneger, T., Mosconi, P., & Zwahlen, M. (2003). Women's perception of the benefits of mammography screening: population-based survey in four countries. International journal of epidemiology, 32(5), 816-821.

[26]Parker, L., Carter, S., Williams, J., Pickles, K., & Barratt, A. (2017). Avoiding harm and supporting autonomy are under-prioritised in cancer-screening policies and practices. European Journal of Cancer, 85, 1-5.

[27]Willis, K. (2012). Choice, Trust and Risk-The Policy Context and Mammography Screening. In Mammography: recent advances (pp. 3-24). InTech Publishers, Croatia.

[28]Higgins, E. T. (2000). Making a good decision: value from fit. American Psychologist, 55(11), 1217-1227.

[29]Higgins, E. T. (1997). Beyond pleasure and pain. American Psychologist, 52(12), 1280-1300.

[30]Keller, P. A. (2006). Regulatory focus and efficacy of health messages. *Journal of Consumer Research, 33(1),* 109-114.

[31]Higgins, E. T., Friedman, R. S., Harlow, R. E., Idson, L. C., Ayduk, O. N., & Taylor, A. (2001). Achievement orientations from subjective histories of success: promotion pride versus prevention pride. *European Journal of Social Psychology, 31(1),* 3-23.

[32]Cesario, J., Higgins, E. T., & Scholer, A. A. (2008). Regulatory fit and persuasion: basic principles and remaining questions. *Social Personality Psychological Compass*, *2*, 444-63.

[33]Cesario, J., Grant, H., & Higgins, E. T. (2004). Regulatory fit and persuasion: transfer from' feeling right.' *Journal of Personality and Social Psychology, 86(3),* 388-404.

[34]Motyka, S., Grewal, D., Puccinelli, N. M., Roggeveen, A. L., Avnet, T., Daryanto, A., ... & Wetzels, M. (2014). Regulatory fit: A meta-analytic synthesis. Journal of Consumer Psychology, 24(3), 394-410.

[35]Zhao, G., & Pechmann, C. (2007). The impact of regulatory focus on adolescents' response to antismoking advertising campaigns. Journal of Marketing Research, XLIV, 671-6

[36]Spiegel, S., Grant-Pillow, H., & Higgins, E. T. (2004). How regulatory fit enhances motivational strength during goal pursuit. European Journal of Social Psychology, 39, 39–54.

[37]Uskul, A. K., Keller, J., & Oyserman, D. (2008). Regulatory fit and health behavior. Psychology and Health, 23(3), 327-346.

[38]Ludolph, R., & Schulz, P. J. (2015). Does regulatory fit lead to more effective health communication? A systematic review. Social science & medicine, 128, 142-150.

[39]Faul, F., Erdfelder, E., Lang, A. G., & Buchner, A. (2007). G*Power 3: A flexible statistical power analysis program for the social, behavioural, and biomedical sciences. *Behavior Research Methods*, 39, 175-191.

[40]Freitas, A. L., & Higgins, E. T. (2002). Enjoying goal-directed action: The role of regulatory fit. *Psychological science*, 13(1), 1-6.

[41] Kees, J. (2011). Advertising framing effects and consideration of future consequences. *The Journal of Consumer Affairs*, 45(1), 7-32.

[42]Consedine, N. S., Magai, C., Krivoshekova, Y. S., Ryzewicz, L., & Neugut, A. I. (2004). Fear, anxiety, worry, and breast cancer screening behavior: a critical review. *Cancer Epidemiology and Prevention Biomarkers, 13(4),* 501-510.

[43]Hay, J. L., McCaul, K. D., & Magnan, R. E. (2006). Does worry about breast cancer predict screening behaviors? A meta-analysis of the prospective evidence. *Preventive medicine, 42(6)*, 401-408.

[44]Nekhlyudov, L., Ross-Degnan, D., & Fletcher, S. W. (2003). Beliefs and expectation of women under 50 years old regarding screening mammography: a qualitative study. *Journal of General Internal Medicine, 18(3),* 182-9.

[45]Nabi, R. L., (2016). Laughting in the face of fear (of disease detection): using humor to promote cancer self-examination behavior. Health Communication, 31(7), 873-883.

Figure 1. Flowchart of the Study 1.

Figure 2. Flowchart of the Study 2.

Figure 1. Flowchart of the Study 1.

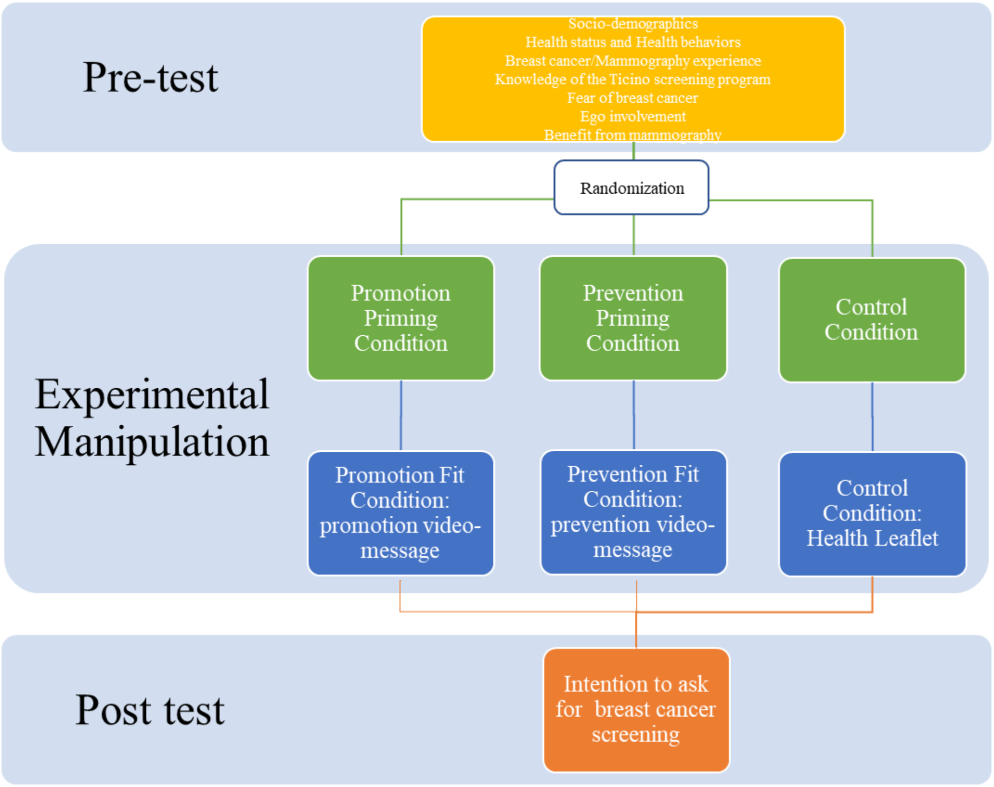

Figure 2. Flowchart of the Study 2.

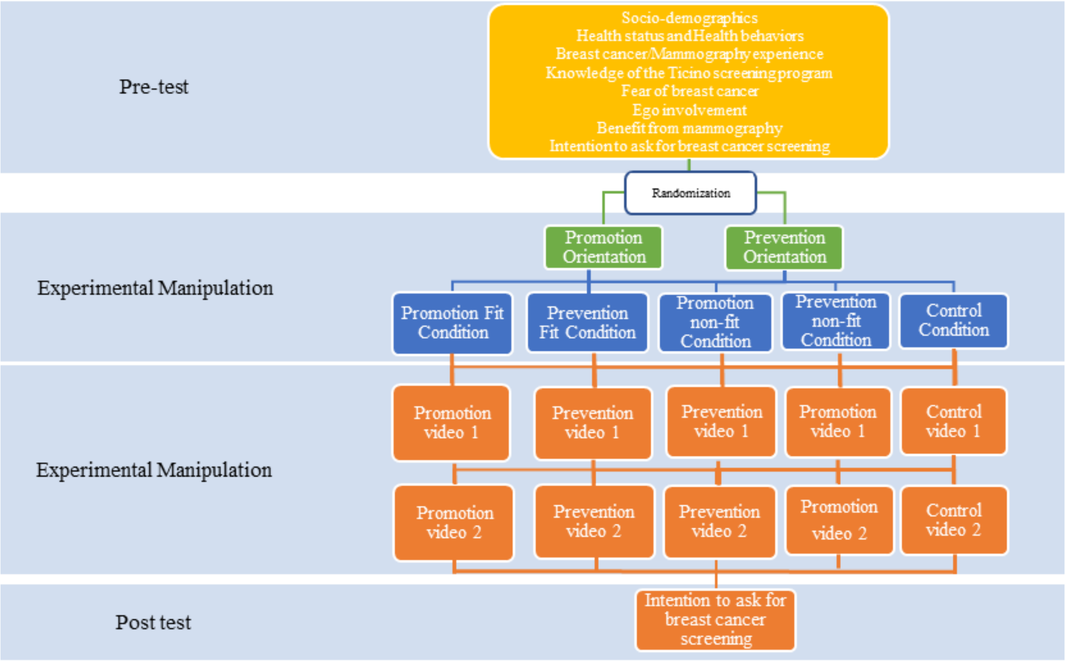

SUPPLEMENTAL MATERIAL

An Application of the Theory of Regulatory Fit to Promote Adherence to Evidence-Based Breast Cancer Screening Recommendations Reducing Unnecessary Breast Cancer Mammography in Women Below Under 50: Experimental vs. Longitudinal Evidence

Serena Petrocchi, Ramona Ludolph, Nanon H. Labrie, Peter J. Schulz

Correspondence to:

Dr. Serena Petrocchi, Institute of Communication and Health, Università della Svizzera italiana, Via Buffi 13, 6900 Lugano, Switzerland.

E-mail: serena.petrocchi@usi.ch

Phone: 0041 (0) 58 666 4821

For peer review only

Study 1

Pre-test measures

Health Status and Healthy Lifestyle. Questions measured overall health status as perceived by the participants on a 5-point Likert scale and healthy lifestyle behaviours (i.e., diet, physical activity, smoking habits, alcohol consumption; see,[1].

Breast Cancer/Mammography Experience and Knowledge of the Ticino screening program. Participants replied to a set of questions on: past diagnosis of breast cancer among first-grade relatives,[2], if they had a mammography in the past, if doctor recommended the mammography, if they had a breast biopsy, if they know the breast cancer screening program in Ticino, and its age thresholds.

Fear of Breast Cancer. Four of the original 8 items of the Fear of Breast Cancer scale,[3,4] were administered. Items asked participants to rate their emotional reaction about breast cancer saying how much they agreed with the statements 'When I think about breast cancer, I feel nervous (or: I get upset, I get jittery, I feel anxious)'. Participants replied on a 5-point Likert scale from 1 (strongly disagree) to 5 (strongly agree). Data from the present sample show that internal consistency was good, Cronbach's $\alpha = .88$, rs > .73, as well as the factor structure, χ^2 (1) = 2.04, p = .15, CFI = .99, RMSEA = .05.

Ego-involvement. The Personal Involvement Inventory,[5] were administered measuring participants' involvement in breast cancer screening through affective and cognitive adjectives because previous research has found that . The scale was administered as a 7-digit semantic differential (e.g., important/unimportant, relevant/irrelevant or worthless/valuable). The original item 'of concern to me/of not concern to me' was deleted based on results of a previous study,[4]. Data from the present sample show that internal consistency was good, Cronbach's $\alpha = .91$, rs > .71, as well as and the factor structure, χ^2 (5) = 11.34, p = .04, CFI = .99, RMSEA = .06.

Perceived benefits of mammography screening. The perceived benefit of mammography screening was measured by four items,[6]: 'Having a mammogram will help me find breast lumps

For peer review only

early'; 'If I find a lump early through a mammogram, my treatment for breast cancer may not be as bad'; 'Having a mammogram is the best way for me to find a very small breast lump'; 'Having a mammogram will decrease my chance of dying from breast cancer'. Participants replied on a 5-point Likert-scale from 1 (strongly disagree) to 5 (strongly agree). Data from the present sample show that internal consistency was modest, Cronbach's $\alpha = .75$, $rs > .49$ and the factor structure was good, χ^2 (1) = .51, p = .47, CFI = 1, RMSEA = .00.

Experimental manipulation

Regulatory Focus Priming Procedure. Prevention induced participants were asked to list two of their current obligations and then write down five actions they could take to avoid failure in fulfilling them,[7]. Promotion induced participants were asked to list two aspirations and write down five actions they could take to ensure their accomplishment,[7].

Video Messages. Participants in the promotion fit condition watched a video message emphasizing promotion concerns (i.e., they should adhere to evidence-based recommendations on mammography screening for safety and health protection reasons). Participants in the prevention fit condition watched a video emphasizing prevention concerns (i.e., they should not abstain from following the evidence-based recommendations on mammography screening to avoid negative/side effects). Participants in the control group did not receive any priming and read a general health leaflet. See Supplemental Table 1 for details of the voice-text of the two video messages and the control leaflet.

For peer review only

Supplemental Table 1. Voice-text of the video messages and control leaflet of the Study 1.

STUDY 1

Promotion video-message | **Prevention video-message**

The mammography screening is a method for the early detection of breast cancer. Using x-rays, mammograms can identify very small tumours generally longer before they are palpable. Mammograms is a method that is used early, often even without symptoms. In a screening program, experts recommend mammography from the age of 50. Here in Ticino, women aged 50 and over are invited to voluntarily undergo mammography every two years at one of the accredited Radiology Centres. For most women between the ages of 50 and 69, the benefits of screening are greater than the risks, but nevertheless it is important to be properly informed to make the best decision about mammography.

Promotion video-message	Prevention video-message
**To protect their health, women under the age of 50, without a medical indication or family history of cancer, are excluded from the program.**	**To avoid adverse effects on their health, women under the age of 50 without a medical indication or family history of cancer are excluded from the program.**

Now I would like to explain the scientific reasoning behind the recommendation to not undergo a mammogram without a medical reason. Anyway, in case of doubt or symptoms, I suggest to contact your doctor. So, you are asking why women under the age of 50 are excluded from mammography screening. Scientific research shows that for women between the ages of 50 and 69 mammography screening is the most effective method for the early detection of breast cancer and for reducing the mortality rate associated with it. In contrast, for young women between the ages of 30 and 49, the disadvantages and risks to health are greater than the benefits. This is mainly due to the fact that women before menopause have a denser breast tissue.

Promotion video-message	Prevention video-message
**Given the reasons I have just presented, one should avoid undergoing a mammogram early to prevent negative consequences.**	**Given the reasons I have just presented, to early undergo a mammogram can lead to negative consequences.**

For example, mammography could show anomalies that, after additional diagnostic tests, could be proved to be benign. This type of error is called a false positive. If for women aged between 50 and 69 this risk is minimal, for young women is higher due to the denser breast tissue. In addition, breast cancer could not be seen by mammograms because it is too small and therefore the exam may appear normal although cancer is present. This is a false-negative result. These risks always exist, but they are higher for young women. As all medical testing, waiting for the outcome of the mammogram can generate a state of anxiety and the procedure sometimes can be perceived as painful. Radiation exposure also have health consequences. Although the exposure is minimal, for women under the age of 50 the risk is higher than the benefits of mammography. Furthermore, screening could lead to over-treatment for tumours that are benign. Over diagnosis represents approximately 1-10% of diagnosed cancers. This would expose young women to the negative effects of anti-cancer therapies, without a real need. In the absence of scientific evidence of the effectiveness of mammographic screening for young women, the inclusion of young women in the program would entail additional costs for society. These financial resources could be used to prevent other diseases.

Promotion video-message	Prevention video-message
**For these reasons, it is recommended that young women follow the indications for breast cancer screening.**	**For these reasons, it is recommended that young women do not ignore the indications related to breast cancer screening.**

Control Leaflet

Healthy eating associated with an active lifestyle is a useful way for disease prevention. An adequate and balanced diet plan guarantees an optimal supply of nutrients to meet the needs of your body. A balanced diet also allows to receive substances that play a protective and/or preventive role against diseases.
This booklet - thought for people of all ages without any particular diseases - explains the scientific reasoning behind the recommendation to follow a healthy diet even in the absence of particular weight or health disorders. Anyway, in case of doubts or problems, we suggest to contact your doctor.

'Man is what he eats.'

We eat several times a day, for a lifetime. With a life expectancy around 80, this corresponds to approximately 85.000 meals. Considering that each meal lasts an average of thirty minutes, we spend at least five years at a table. Adding the meal preparation time, the count easily rises to ten years or more. Nutrition is therefore a topic that deserves special attention. Balanced meals and a healthy lifestyle give a fundamental contribution to our daily well-being and efficiency.

«Fast casual» instead of «fast food»

'Fast casual' means eating healthy and fast and it is not a contradiction. The 'snack culture' is an old idea. Those who want to have a quick meal, however, must pay attention to its composition: dairy products, fruit, vegetables, salads and whole meal products are the basis for a new, healthy 'fast casual' menu.

The right fats for every need

The choice of fats must be made according to the intended use. Fats and oils, if heated for a long time, change with chemical reactions that can create unwanted substances harmful to health. In order to avoid such reactions, it is advisable to cook with fats that mainly contain saturated fatty acids, which are more stable at high temperatures.

Eating healthy is easy

Food provides the body with both the necessary energy and nutrients that allow it to function properly. The diet must therefore provide a correct caloric intake and a sufficient amount of nutrients. No food is so complete that it contains everything the body needs. The basic rule is therefore to eat everything and in a varied way.

Food pyramid

The food pyramid facilitates the quantitative choice between the various food groups. It is a useful tool for all those who want to have a healthy diet. It provides clear indications on what to eat, how often, and in what quantity.

How do I interpret it?

A balanced diet requires the foods at the base of the pyramid be consumed in greater quantities. Climbing up to the vertex, the quantities of food consumed should be limited. Nothing is forbidden, every food finds its place in a balanced diet, but the recommended quantities will depend on its location in the pyramid.

Give food the importance that deserves, eating healthier. Your health and well-being will be better!

Note: grey rounded rectangles show the common parts of promotion and prevention video-messages; orange rounded rectangles show the promotion video-message specific parts (text in bold); blue rounded rectangles show the prevention video-message specific parts (text in bold); the green rounded rectangle shows the content of the control leaflet. The Videos created for Study 1 can be retrieved from https://youtu.be/mperSG5_9yQ and https://youtu.be/KnhRUnDoSV0. Both videos last 3:28 minutes. The videos created for Study 2 can be retrieved from https://youtu.be/btM3HrvYDlQ, https://youtu.be/BZPjFPUQuvw, https://youtu.be/-lXzGpcmzD4, https://youtu.be/jRi8Y-sZvSc. A translation of the Italian voice-over has been provided in this Table.

For peer review only

Post-test Measures

Intention to ask for breast cancer screening. Intention was measured by the question "I am evaluating the idea to have a mammography screening for breast cancer in the next 2-3 years",[8]. Two further questions were added: "I have the intention to have a mammography screening for breast cancer in the next 2-3 years" and "I will take an appointment for a mammography screening for breast cancer in the next 2-3 years". Participants replied on a 5-point scale from 1 (definitely yes) to 5 (definitely not). Data from the present sample show that internal consistency was good, Cronbach's $\alpha = .97$, rs > .94.

For peer review only

Study 2

Measures

Pre-test Measures

Pre-test covariates were measured as for study 1. Intention to ask for breast cancer screening was asked during the pre-test with the three items applied in Study 1.

Experimental manipulation

Trait Regulatory Orientation. The Regulatory Focus Questionnaire,[9] was applied in the pre-test phase. The questions asked how frequently several specific events occur in the participant's life. Six questions capture the promotion focus, and the other five the prevention focus. Participants replied on a 5-point scale from 1 (never) to 5 (very often). The scores for promotion and prevention scales were calculated averaging the answers on given items: data show good internal consistency for both promotion, $\alpha = .66$, rs > .33, and prevention, $\alpha = .74$, rs > .47. The individual's chronic orientation was calculated following the original procedure,[9].

Video Messages. Six video-messages were developed for the present study:

- Two video-messages emphasising prevention concerns;
- Two video-messages emphasising promotion concerns;
- Two video-messages without any prompt to regulatory orientation.

Supplemental Table 2 shows the content of the voice-text of the six video-messages.

Post-test Measures

Intention to ask for breast cancer screening. As for Study 1.

For peer review only

Supplemental Table 2: voice-text of the video messages for Study 2.

STUDY 2			
	Promotion video-messages	Prevention video-messages	Control video-messages
VIDEO 1	For the early detection of breast cancer, experts recommend mammography to women aged 50 and over. Mammography is the most effective medical examination for the early detection of breast cancer. It consists of an X-ray exposure that allows you to identify even very small tumors, before they are palpable or recognizable. Women over the age of 50 are invited to undergo a mammogram every 2 years at an accredited radiology center. For women between 50 and 69, the benefits of the exam outweigh the risks. And before the age of 50?		
VIDEO 1	**To respect their health, women under 50 are excluded from the breast cancer screening program.** (PROMOTION FOCUS)	**To avoid adverse health effects, women under 50 are excluded from the breast cancer screening program.** (PREVENTION FOCUS)	**Women under 50 are excluded from the breast cancer screening program.** (CONTROL GROUP)
VIDEO 1	It is a medical recommendation: before the age of 50, the risks of the examination are greater than the benefits.		
VIDEO 1	**In the absence of proven risk, to take care of your health, doctors advise to not undergo a mammogram before the age of 50.** (PROMOTION FOCUS)	**In the absence of proven risk, to avoid adverse effects for your health, doctors advise to not undergo a mammogram before the age of 50.** (PREVENTION FOCUS)	**In the absence of proven risk, doctors advise to not undergo a mammogram before the age of 50.** (CONTROL GROUP)
VIDEO 1	Mammography is a breast test that allows you to detect even many small tumours. Over 50 years, it is done every two years. Women under the age of 50 are excluded from the breast cancer screening program, except in case of genetic predisposition or family history of breast cancer. What are the reasons for this decision?		
VIDEO 1	**The observance of the age threshold determines a decreasing of the probability of false positives: breast anomalies that are benign form.** (PROMOTION FOCUS)	**The observance of the age threshold allows avoiding the negative consequences caused by false positives: breast abnormalities that are benign form.** (PREVENTION FOCUS)	**In young women, false positives are highly likely. False positive are breast anomalies that are benign form.** (CONTROL GROUP)
VIDEO 1	The risk of false positives in young women is higher, because the breast tissue is denser.		
VIDEO 1	**It is advised not to make mammograms before the age of 50 in order not to expose themselves to anti-cancer treatments not recommended as they are often directed to benign anomalies.** (PROMOTION FOCUS)	**It is advisable not to make mammograms before the age of 50 to avoid exposure to non-recommended anti-cancer treatments as they are often directed to benign anomalies.** (PREVENTION FOCUS)	**The breast cancer screening could lead to an exposition of non-recommended anti-cancer treatments, as they are often directed to benign anomalies.** (CONTROL GROUP)
VIDEO 1	In fact, breast cancer is much rarer in women under the age of 50.		
VIDEO 1	**Excluding younger women from screening allows them to be protected from unnecessary radiation exposure. Furthermore, this choice promotes psychological well-being against stress and anxiety. For these reasons, mammographic screening involves only women over 50 years. If you are under 50 and want to take care of your health, we recommend that you respect the age threshold.** (PROMOTION FOCUS)	**Excluding younger women from screening allows you to avoid unnecessary radiation exposure. Furthermore, this choice avoids psychological discomforts such as stress and anxiety. For these reasons, mammographic screening involves only women over 50 years. If you are under 50 years old and want to avoid negative consequences for your health, we recommend that you respect the age threshold.** (PREVENTION FOCUS)	**Radiation exposure poses health risks. In the case of mammography, the exposure is minimal, but for women under the age of 50 the risk is higher than the benefits. Furthermore, as any other medical procedure, waiting for the outcome can generate anxiety and stress. For these reasons, mammographic screening involves only women over 50 years. If you are under 50 years old, we recommend that you respect the age threshold.** (CONTROL GROUP)
VIDEO 1	A conscious prevention, it's worthit!		

Note: grey rounded rectangles show the common parts of the video-messages; the orange rounded rectangles show the promotion video-message specific parts (text in bold); the blue rounded rectangles show the prevention video-message specific parts (text in bold); the green rounded rectangle shows the content of the control leaflet.

References

[1]Shim, M., Kelly, B., & Hornik, R. (2006). Cancer information scanning and seeking behavior is associated with knowledge, lifestyle choices, and screening. *Journal of Health Communication, 11(2),* 157–172.

[2]Daley, M. B., Lerman, C. L., Ross, E., Schwartz, M. D., Sands, C. B., & Masny, A. (1996). Gail model breast cancer risk components are poor predictors of risk perception and screening behaviour. *Breast Cancer Research and Treatment, 41,* 59–70.

[3]Champion, V. L., Skinner, C. S., Menon, U., Rawl, S., Giesler, R. B., Monahan, P., & Daggy, J. (2004). A breast cancer fear scale: Psychometric development. *Journal of Health Psychology, 9(6),* 753-762.

[4] Labrie, N. H., Ludolph, R. A., & Schulz, P. J. (2020). Mammography perceptions and practices among women aged 30–49: The role of screening programme availability and cultural affiliation. Patient Education and Counseling, 103(2), 369-375.

[5]Zaichkowsky, J. L. (1994). The personal involvement inventory: Reduction, revision, and application to advertising. Journal of Advertising, 23 (4), 59-70.

[6]Champion, V. L., Mohahan, P. O., Springston, J. K., Russell, K, Zollinger, T. W., Saywell, R. M. Jr., & Maraj, M. (2008). Measuring mammography and breast cancer beliefs in African American women. *Journal of Health Psychology, 13(6),* 827-837.

[7] Freitas, A. L., & Higgins, E. T. (2002). Enjoying goal-directed action: The role of regulatory fit. *Psychological science*, 13(1), 1-6.

[8]Hersch J., Barratt A., Jansen J., Irwig L., McGeechan K., Jacklyn G., & McCaffery K. (2015). Use of a decision aid including information on over detection to support informed choice about breast cancer screening: a randomized controlled trial. *The Lancet, 385(9978),* 1642-1652.

[9] Higgins, E. T., Friedman, R. S., Harlow, R. E., Idson, L. C., Ayduk, O. N., & Taylor, A. (2001). Achievement orientations from subjective histories of success: promotion pride versus prevention pride. *European Journal of Social Psychology, 31(1),* 3-23.

For peer review only

STROBE 2007 (v4) Statement—Checklist of items that should be included in reports of *cross-sectional studies*

Section/Topic	Item #	Recommendation	Reported on page #
**Title and abstract**	1	(*a*) Indicate the study's design with a commonly used term in the title or the abstract	#1; #2
		(*b*) Provide in the abstract an informative and balanced summary of what was done and what was found	#2
**Introduction**			
Background/rationale	2	Explain the scientific background and rationale for the investigation being reported	#4-7
Objectives	3	State specific objectives, including any prespecified hypotheses	#6-7
**Methods**			
Study design	4	Present key elements of study design early in the paper	#7; #11
Setting	5	Describe the setting, locations, and relevant dates, including periods of recruitment, exposure, follow-up, and data collection	#8; #11
Participants	6	(*a*) Give the eligibility criteria, and the sources and methods of selection of participants	#7-8; #10
Variables	7	Clearly define all outcomes, exposures, predictors, potential confounders, and effect modifiers. Give diagnostic criteria, if applicable	#8; #12
Data sources/ measurement	8*	For each variable of interest, give sources of data and details of methods of assessment (measurement). Describe comparability of assessment methods if there is more than one group	#8; #11
Bias	9	Describe any efforts to address potential sources of bias	#8-10; #10-12
Study size	10	Explain how the study size was arrived at	#7; #11
Quantitative variables	11	Explain how quantitative variables were handled in the analyses. If applicable, describe which groupings were chosen and why	#8-9; #12
Statistical methods	12	(*a*) Describe all statistical methods, including those used to control for confounding	#9; #12
		(*b*) Describe any methods used to examine subgroups and interactions	#9-11; #12
		(*c*) Explain how missing data were addressed	#9; #12
		(*d*) If applicable, describe analytical methods taking account of sampling strategy	Not applicable
		(*e*) Describe any sensitivity analyses	Not applicable
**Results**			

For peer review only

Participants	13*	(a) Report numbers of individuals at each stage of study—eg numbers potentially eligible, examined for eligibility, confirmed eligible, included in the study, completing follow-up, and analysed	#7; #10-11
		(b) Give reasons for non-participation at each stage	#7; #10-11
		(c) Consider use of a flow diagram	#8
Descriptive data	14*	(a) Give characteristics of study participants (eg demographic, clinical, social) and information on exposures and potential confounders	#7; #12
		(b) Indicate number of participants with missing data for each variable of interest	#9; #12
Outcome data	15*	Report numbers of outcome events or summary measures	#9-12
Main results	16	(*a*) Give unadjusted estimates and, if applicable, confounder-adjusted estimates and their precision (eg, 95% confidence interval). Make clear which confounders were adjusted for and why they were included	#9-12
		(*b*) Report category boundaries when continuous variables were categorized	Not applicable
		(*c*) If relevant, consider translating estimates of relative risk into absolute risk for a meaningful time period	Not applicable
Other analyses	17	Report other analyses done—eg analyses of subgroups and interactions, and sensitivity analyses	Not applicable
**Discussion**			
Key results	18	Summarise key results with reference to study objectives	#12-16
Limitations	19	Discuss limitations of the study, taking into account sources of potential bias or imprecision. Discuss both direction and magnitude of any potential bias	#15
Interpretation	20	Give a cautious overall interpretation of results considering objectives, limitations, multiplicity of analyses, results from similar studies, and other relevant evidence	#12-16
Generalisability	21	Discuss the generalisability (external validity) of the study results	#12-16
**Other information**			
Funding	22	Give the source of funding and the role of the funders for the present study and, if applicable, for the original study on which the present article is based	#16

*Give information separately for cases and controls in case-control studies and, if applicable, for exposed and unexposed groups in cohort and cross-sectional studies.

Note: An Explanation and Elaboration article discusses each checklist item and gives methodological background and published examples of transparent reporting. The STROBE checklist is best used in conjunction with this article (freely available on the Web sites of PLoS Medicine at http://www.plosmedicine.org/, Annals of Internal Medicine at http://www.annals.org/, and Epidemiology at http://www.epidem.com/). Information on the STROBE Initiative is available at www.strobe-statement.org.

For peer review only

BMJ Open

An Application of the Theory of Regulatory Fit to Promote Adherence to Evidence-Based Breast Cancer Screening Recommendations: Experimental vs. Longitudinal Evidence

Journal:	*BMJ Open*
Manuscript ID	bmjopen-2020-037748.R2
Article Type:	Original research
Date Submitted by the Author:	09-Sep-2020
Complete List of Authors:	Petrocchi, Serena; Università della Svizzera Italiana,<br>Ludolph, Ramona; Università della Svizzera Italiana<br>Labrie, Nanon ; Vrije Universiteit Amsterdam, Athena Institute, Faculty of Beta Sciences<br>Schulz, Peter; Universita della Svizzera Italiana, Institute of Communication & Health
**Primary Subject Heading**:	Communication
Secondary Subject Heading:	Health policy
Keywords:	PUBLIC HEALTH, Breast tumours < ONCOLOGY, PREVENTIVE MEDICINE

SCHOLARONE™
Manuscripts

I, the Submitting Author has the right to grant and does grant on behalf of all authors of the Work (as defined in the below author licence), an exclusive licence and/or a non-exclusive licence for contributions from authors who are: i) UK Crown employees; ii) where BMJ has agreed a CC-BY licence shall apply, and/or iii) in accordance with the terms applicable for US Federal Government officers or employees acting as part of their official duties; on a worldwide, perpetual, irrevocable, royalty-free basis to BMJ Publishing Group Ltd ("BMJ") its licensees and where the relevant Journal is co-owned by BMJ to the co-owners of the Journal, to publish the Work in this journal and any other BMJ products and to exploit all rights, as set out in our licence.

The Submitting Author accepts and understands that any supply made under these terms is made by BMJ to the Submitting Author unless you are acting as an employee on behalf of your employer or a postgraduate student of an affiliated institution which is paying any applicable article publishing charge ("APC") for Open Access articles. Where the Submitting Author wishes to make the Work available on an Open Access basis (and intends to pay the relevant APC), the terms of reuse of such Open Access shall be governed by a Creative Commons licence – details of these licences and which Creative Commons licence will apply to this Work are set out in our licence referred to above.

Other than as permitted in any relevant BMJ Author's Self Archiving Policies, I confirm this Work has not been accepted for publication elsewhere, is not being considered for publication elsewhere and does not duplicate material already published. I confirm all authors consent to publication of this Work and authorise the granting of this licence.

For peer review only

An Application of the Theory of Regulatory Fit to Promote Adherence to Evidence-Based Breast Cancer Screening Recommendations: Experimental vs. Longitudinal Evidence

Serena Petrocchi, Ramona Ludolph Ramona, Nanon H.M. Labrie, Peter J. Schulz

Corresponding author
Dr Serena Petrocchi
Institute of Communication and Health
Università della Svizzera italiana
Via Buffi 13
6900 Lugano
Switzerland
T: 0041 (0)58 666 4821
Fax: 0041 (0)58 666 4000
E: serena.petrocchi@usi.ch

Dr Ramona Ludolph:
Institute of Communication & Health
Università della Svizzera italiana
Via Buffi 13
6900 Lugano
Switzerland

Dr Nanon H.M. Labrie
Athena Institute
Vrije Universiteit Amsterdam
De Boelelaan 1105, 1081 HV Amsterdam
The Netherlands: n.h.m.labrie@vu.nl

For peer review only - http://bmjopen.bmj.com/site/about/guidelines.xhtml

Prof. Peter J. Schulz
Institute of Communication & Health
Università della Svizzera italiana
Via Buffi 13
6900 Lugano
Switzerland: peter.schulz@usi.ch

Keywords: breast cancer screening; theory of regulatory fit; breast cancer prevention; mammography

Word count: 4168 words

For peer review only - http://bmjopen.bmj.com/site/about/guidelines.xhtml

Abstract

Objectives. To reduce overtreatment caused by overuse of screening, it is advisable to reduce the demand for mammography screening outside the recommended guidelines among women who are not yet eligible for inclusion in systematic screening programmes. According to principles of regulatory fit theory, people make decisions motivated by either orientation to achieving and maximizing gains or avoiding losses. A study developed in two phases investigated whether video messages, explaining the risks and benefits of mammography screening for those not yet eligible, are perceived as persuasive. **Design**. Phase 1 was an experimental study in which women's motivation orientation was experimentally induced and then they were exposed to a matching video message about mammography screening. A control group received a neutral stimulus. Phase 2 introduced a longitudinal component to Study 1, adding a condition in which the messages did not match with the group's motivation orientation. Participants' natural motivation orientation was measured through a validated questionnaire. **Participants**. 360 women participated in Phase 1 and other 292 in Phase 2. Participants' age ranged from 30 to 45 years, and had no history of breast cancer or known BRCA 1/2 mutation. **Results**. In Phase 1, a match between participants' motivation orientation and message content decreased the intention to seek mammography screening outside the recommended guidelines. Phase 2, however, did not show such an effect. Fear of breast cancer and risk perception were significantly related to intention to seek mammography screening. **Conclusions**. Public health researchers should consider reducing the impact of negative emotions (i.e. fear of breast cancer) and risk perception when promoting adherence to evidence-based breast cancer screening recommendations.

Keywords: breast cancer screening; theory of regulatory fit; breast cancer prevention; mammography screening

Strengths and Limitations of this Study

- An experimental study (Phase 1) and an experimental study with a longitudinal component (Phase 2) were implemented applying principles from the theory of regulatory fit.
- An individual's goal-pursuit orientation was induced in Phase 1 through a priming technique, and measured through a validated questionnaire in Phase 2.
- Messages were tailored to create a match (or not) between message content and the individual's goal-pursuit orientation.
- Limitations of the studies included dropout rates (Phase 2) and selection bias (due to cancer fear).

Breast cancer is one of the most common forms of cancer in women worldwide and the principal cause of cancer-related death in the female population,[1]. To promote early diagnosis, many EU countries have introduced systematic breast cancer screening programs,[2]. However, the age threshold to start inviting women to screening is in dispute,[3-5]. The balance between the benefits (i.e., reducing breast cancer mortality) and the harm associated with mammography (i.e. x-ray exposure, over diagnosis and false positive results; see,[4-8]) is uncertain. Technologies for breast cancer screening have been constantly evolving, affecting evidence quality and suggested recommendations,[9]. For these reasons recommended age for starting screening have varied from 40,[10], to 45,[11,12], to 50 years,[13,14].

In the last years, there has been a vast amount of research on screen intention, including barriers, enablers, and how to get women with characteristics matching with the recommended guidelines to adhere to the screening programs,[see 15-17]. There was also a progressive shift from persuading women to undergo screening to increasing their informed decision making[18]. Targeted information programs and invitation materials encouraging women to learn about the screening procedures increased levels of knowledge and supported decision-making about their participation,[19,20]. Web-based dynamic decision aids, including pros, cons, controversies, and overdiagnosis-overtreatment issues, have been found to improve the quality of information without reducing the screening participation rate,[21].

Other research tested communication programs intending to inform women approaching 70 years of age about the benefits and harms of continuing screening,[22,23]. Similarly, non high-risk women below the recommended age threshold seek and receive mammography screenings outside the suggested guidelines in the U.S.,[24,25], Switzerland,[26,27], Germany,[28], and The Netherlands,[29]. Studies show that women tend to overestimate the mortality reduction determined by

For peer review only - http://bmjopen.bmj.com/site/about/guidelines.xhtml

breast cancer screening,[30,31] and that they have unrealistic expectations regarding screening as reducing the risk of breast cancer,[32]. Moreover, social pressure in favour of breast cancer screening may stimulate a sense of moral obligation to participate,[33,34], even among young women.

Given the above-mentioned considerations, women under the age threshold for systematic breast cancer screening may consider the recommendation to avoid screening as counterintuitive, although scientifically supported, because of social pressure and the belief that cancer screening can save lives. The present research aimed to promote adherence to evidence-based recommendations on breast cancer screening among young women by activating a motivation system, such as regulatory orientation,[35].

Theory of Regulatory Fit

According to a popular psychological theory proposed by [36], people show one of two regulatory orientations, which determines how they pursue their goals. They either show a promotion-focused orientation, meaning they eagerly strive towards the realization of desired outcomes, or they show a prevention-focused orientation, emphasizing the prevention of errors and losses and making them safety-driven, [36,37]. While every individual has a natural tendency to lean more towards one orientation than the other, thus making it a measurable trait,[38], the regulatory orientation can also be experimentally induced,[35,36,39].

If individuals adopt a behaviour or processes a message highlighting goal-pursuit strategies that match their regulatory orientation, they experience a phenomenon called “regulatory fit”,[35]. For example, if a person with a promotion orientation reads a message highlighting strategies to achieve gains, a fit condition occurs. The same applies to someone with a prevention orientation processing a

message emphasizing strategies to avoid losses. Such a fit or match causes an “it just feels right” perception, increasing the perceived value of the behaviour[40].

The application of regulatory fit in the area of health communication is beneficial across various health contexts and outcomes,[41]. Regulatory fit has been consistently found to influence outcomes such as evaluation, behaviour and behavioural intention,[42]. Some authors [40] showed that this “it-just-feels-right” experience is also transferred to the context of persuasion, with positive effect of regulatory fit on the perceived persuasiveness of a message. A study by [43] in the context of tobacco use prevention among adolescents is in line with this finding. The effects of regulatory fit have also been extensively studied in the context of disease prevention and health promotion,[44,45]. In particular, some authors [43] applied the principles of regulatory fit to inform people about the benefits of regular cancer screenings. A systematic review,[41] finds that the use of the principle of regulatory fit has the potential to increase the effectiveness of health communication across a range of health contexts and outcomes, making it a promising tool for tackling the problem of unwarranted demand for mammography screening outside the recommended guidelines.

No previous studies have tested messages designed according to the assumptions of regulatory fit to influence the intention to *not* engage in disease detection screening. This would challenge the intuitive perception that breast cancer screening leads to a mortality reduction determined by breast cancer,[30,31] and the unrealistic expectations regarding screening as reducing the risk of breast cancer,[32]. The purpose of the present research was to test whether health messages framed to correspond with a woman's regulatory orientation are effective in *reducing* the intention to ask for breast cancer screening in non high-risk women under the age of

For peer review only - http://bmjopen.bmj.com/site/about/guidelines.xhtml

45, according to the local mammography screening guidelines. The following hypotheses have been tested:

HP1: a fit between the message frame and the regulatory orientation would lead to an immediate reduction of the intention to ask for breast cancer screening, in non-high-risk women under the age threshold indicated by the local guidelines.

HP2: a fit between the message frame and the regulatory orientation would lead to a reduction of the intention to ask for breast cancer screening, stable over time.

To this end, a study has been developed organized in two distinct phases: Phase 1 was an experimental study testing HP1, while Phase 2 added a longitudinal component and tested HP2.

Methods

Participants

Phase 1

An a priori power analysis applying G*Power 3.1.9.2 ,[46] estimated a sample of 249 participants (α = .05, d = .95, η^2 = 0.05; see,[41]). Participants living in the Italian-speaking, Swiss canton of Ticino completed an online survey from June to September 2016. The research was repeatedly advertised on the Facebook page of the University. Exclusion criteria were: a personal history of breast cancer, BRCA mutations, insufficient fluency in Italian. The survey required women to answer each question before progressing to the next screen; as such there were no missing data. Participants received a 10 CHF supermarket voucher for their participation in Phase 1. Before starting the questionnaire, participants completed a written informed consent by clicking on the corresponding button (i.e. “yes, I want to participate”; “no, I do not want to participate”).

For peer review only - http://bmjopen.bmj.com/site/about/guidelines.xhtml

Five hundred women from 30 to 45 years started the survey: 121 (16%) initiated the pre-test questionnaires but dropped out. Nineteen of the women were excluded from the final sample because they did not complete the experimental manipulation No differences emerged in the pre-test variables between those who filled in only the pre-test (N = 140) and who filled in the entire survey (N = 360). Participants were randomly assigned to prevention fit, promotion fit, and control condition (see Table 1). No differences were found between the intervention groups and the control group on socio-demographic variables.

For peer review only - http://bmjopen.bmj.com/site/about/guidelines.xhtml

Table 1. Demographics of Phase 1 and Phase 2.

	Phase 1			Phase 2				
	**Promotion Fit (N = 122)**	**Prevention Fit (N = 130)**	**Control Group (N = 108)**	**Promotion Fit (N = 58)**	**Promotion Non-fit (N = 57)**	**Prevention Fit (N = 74)**	**Prevention Non-fit (N = 74)**	**Control Group (N = 29)**
**Age** (range 30-45): M and (SD)	36.55 (4.42)	38.07 (4.57)	38.37 (4.79)	38.1 (4.96)	38.53 (4.7)	38.31 (4.44)	37.93 (4.41)	37.02 (4.99)
**Marital Status**								
Married	73 (59%)	77 (60%)	69 (64%)	36 (62%)	41 (72%)	55 (74%)	53 (72%)	22 (76%)
Single	38 (31%)	38 (30%)	26 (24%)	20 (35%)	12 (21%)	14 (19%)	17 (23%)	6 (21%)
Divorced/Separated/Widowed	11 (10%)	15 (10%)	13 (12%)	2 (3%)	4 (7%)	5 (7%)	4 (5%)	1 (3%)
**Educational Level**								
Elementary/Junior School	2 (2%)	2 (2%)	3 (3%)	1 (2%)	-	1 (1%)	4 (5%)	-
High School	44 (34%)	56 (46%)	58 (54%)	18 (31%)	24 (43%)	40 (54%)	28 (38%)	8 (28%)
University or Post University Degree	84 (64%)	64 (52%)	47 (43%)	39 (66%)	33 (57%)	33 (45%)	42 (57%)	21 (72%)
**Occupation**								
Employed	102 (84%)	107 (82%)	74 (69%)	48 (83%)	50 (88%)	57 (77%)	67 (91%)	29 (90%)
Homemaker	11 (9%)	14 (11%)	22 (20%)	4 (7%)	3 (5%)	7 (9%)	6 (8%)	1 (3%)
Unemployed	8 (6%)	7 (5%)	10 (9%)	4 (7%)	4 (7%)	8 (11%)	1 (1%)	2 (7%)
Student	1 (1%)	2 (2%)	2 (2%)	2 (3%)	-	2 (3%)	-	-
**Nationality**								
Swiss	97 (80%)	101 (78%)	73 (68%)	10 (17%)	15 (26%)	16 (18%)	13 (18%)	-
Italian	21 (17%)	23 (18%)	26 (24%)	47 (81%)	40 (70%)	53 (71%)	58 (78%)	27 (93%)
Other	4 (3%)	6 (4%)	9 (8%)	1 (2%)	2 (4%)	5 (7%)	3 (4%)	4 (7%)
**Mother Tongue**								
Italian	117 (96%)	122 (94%)	94 (87%)	54 (93%)	54 (93%)	68 (92%)	71 (96%)	27 (93%)
Other	5 (4%)	8 (6%)	14 (13%)	4 (7%)	4 (7%)	6 (8%)	3 (4%)	2 (7%)

For peer review only - http://bmjopen.bmj.com/site/about/guidelines.xhtml

Phase 2

A priori power analysis estimated a sample size of 312. Recruitment took place from June to October 2017. The research was advertised through the Facebook page of the University and by RCSMedia Group, an Italy-based publishing group that uses participant panels. Inclusion/exclusion criteria were as for Phase 1, with the addition that participants included in Phase 1 could not participate in Phase 2. Participants completed a written informed consent as for Phase 1, and at the end received a 10 CHF/EU supermarket voucher.

973 women aged from 30 to 45 filled in the pre-test questionnaires (i.e., pre-test sample). Completed questionnaires (i.e., analytical sample) were returned by 292 women with an attrition rate of 70%. Comparisons between the pre-test sample and the analytical sample did not yield significant differences. 292 women participated in the research (see Table 1). This time, women aged 30 to 45 living in Ticino and Italy participated. Italian and Ticino-Swiss participants are not only comparable from a cultural and linguistic point-of-view, but also screening guidelines in Ticino and Italy are alike, inviting 50 to74-year-olds biennially for mammography screening. No differences were found among the five groups regarding socio-demographic variables or other pre-test variables.

Process, Measures and Data Collection

Phase 1

A pre-post-test design with two experimental conditions and a control group was applied (see Figure 1 for full details).

[Insert Figure 1 here]

At pre-test, the survey included measures of health status and health behaviours, a set of questions on past diagnosis of breast cancer, mammography,

For peer review only - http://bmjopen.bmj.com/site/about/guidelines.xhtml

biopsy and knowledge of the Ticino screening program. Women were rated on their fear of breast cancer, level of involvement in breast cancer and confidence in the benefit of mammography (see online supplementary material).

Participants were randomly assigned into a promotion fit, prevention fit or control condition. Regulatory priming manipulation was then induced following,[47] procedure (online supplementary material). In the fit conditions, immediately after priming, the participants watched a video-message highlighting goal-pursuit strategies matching with the primed focus (online supplementary material). The control group received a leaflet without any prompt for the regulatory orientation (online supplementary material). In a pilot study, 30 women assessed the survey as clear and understandable. The University's Ethical Committee approved Phase 1 and Phase 2.

Phase 2

A pre-post-test longitudinal design was applied with four experimental conditions, two fit conditions (promotion and prevention), two non-fit conditions (promotion and prevention) and a control group (see Figure 2).

[Insert Figure 2 approx. here]

In the pre-test (T0), participants replied to the same questions as for Phase 1 (see online supplemental material). In Phase 2, the regulatory focus orientation was measured with a questionnaire (online supplemental material), rather than induced as in Phase 1, because working with the trait regulatory focus would be more stable than a primed focus in a longitudinal design. Women were then identified according to their goal-pursuit main orientation (prevention orientation vs. promotion orientation). Subsequently, participants were randomly assigned to the fit or non-fit condition or control group. In other words, randomisation was performed separately for prevention-oriented women and promotion-oriented women to ensure a balanced representation of

For peer review only - http://bmjopen.bmj.com/site/about/guidelines.xhtml

orientations between the match and non-match conditions. Participants in the fit conditions watched two videos (at T1 and T2) emphasizing the fit concerns (online supplemental material). In the non-fit conditions, participants watched two videos (at T1 and T2) emphasizing the non-fit concerns (online supplemental material). In the control group, participants watched two videos (at T1 and T2) treating the topic of breast cancer prevention, but without any regulatory prompt (online supplemental material).

A post-test questionnaire evaluated the women's intention to ask for opportunistic screening (T3). Ten days elapsed between each experimental phase. Three women from the general population assessed the videos as comprehensible and clear. The final survey was tested by fifteen women aged 30 to 45, who assessed it as clear and comprehensible.

Patient and Public Involvement

Phase 1 and Phase 2

Results from previous studies involving participants from Switzerland informed the present research (see,[27]). Participants were not directly involved in the design, conduct, recruitment, reporting or dissemination of the study results. An expert panel, composed of two health communication professionals with expertise on regulatory fit theory, evaluated the message contents and the graphical aspects of the videos.

Analytic Strategy

Phase 1 and Phase 2

In both Phase 1 and Phase 2, data were normalized through reverse scoring and logarithmic transformations and there were no missing data.

For peer review only - http://bmjopen.bmj.com/site/about/guidelines.xhtml

In Phase 1, an ANCOVA tested the main hypothesis (HP1) of the study. The fit vs. control conditions variable was inserted as independent variable. All the variables measured at the pre-test were inserted as covariates. Chi-square tests were conducted to evaluate whether the covariates might interact with the three experimental conditions in determining the intention to ask for breast cancer screening.

In Phase 2, a repeated measure ANCOVA tested the main hypothesis (HP2) of the study. The fit vs. non-fit vs. control conditions variable was inserted as independent variable. All the variables measured at the pre-test were inserted as covariates.

Results

Phase 1

The ANCOVA analysis revealed that women in the two experimental conditions showed less intention to ask for breast cancer screening compared to the women in the control condition. Thus, when there is a fit between individual orientation (i.e., a tendency to promote positive expected outcomes or to prevent negative outcomes for one’s health) and the given message, then a persuasive effect is induced. There was no meaningful difference between the two manipulation conditions. Older women and women with higher levels of fear of breast cancer showed a greater intention to ask for breast cancer screening than younger ones and those with lower levels of fear. This evidence supports the assumption that regulatory orientation represents a motivational system able to overcome the impact of negative emotions and strengthen an individual’s involvement in decision-making orientation. Descriptive data and results from the ANCOVA are displayed in Table 2.

For peer review only - http://bmjopen.bmj.com/site/about/guidelines.xhtml

For peer review only - http://bmjopen.bmj.com/site/about/guidelines.xhtml

Table 2. Descriptive statistics of the pre- and post-test variables with frequencies (% frequencies between brackets) or means (standard deviations between brackets), and results of the analyses.

	Phase 1			Phase 2				
	**Promotion Fit**	**Prevention Fit**	**Control Group**	**Promotion Fit**	**Promotion Non-fit**	**Prevention Fit**	**Prevention Non-fit**	**Control Group**
**PRE-TEST VARIABLES**								
General Health Status	3.88 (.77)	3.77 (.87)	3.7 (.87)	3.79 (.79)	3.63 (.67)	3.66 (.76)	3.70 (.77)	3.76 (.69)
Physical Activity	2.45 (1.85)	2.47 (1.69)	2.43 (1.92)	.78 (.42)	.81 (.39)	.76 (.46)	.72 (.45)	.86 (.35)
Smoking Habits	1.86 (4.85)	1.99 (4.99)	3.32 (6.42)	3.53 (5.4)	3.12 (4.66)	4.93 (5.59)	3.19 (5.15)	7.22 (5.4)
Alcohol Consumption	1.92 (2.79)	1.71 (2.27)	1.42 (2.14)	3 (2.26)	2.66 (2.15)	3.27 (4.13)	2.67 (3.54)	3.1 (4)
Fear of Breast Cancer	3.4 (.85)	3.4 (.81)	3.4 (1)	3.75 (.95)	3.59 (.91)	3.79 (.95)	3.83 (.93)	3.68 (1)
Ego-Involvement	5.9 (1.1)	5.8 (1.27)	5.9 (1.3)	-	-	-	-	-
Benefit for Mammography	3.9 (.62)	3.8 (.62)	4 (.74)	4.1 (.75)	4.12 (.73)	4.16 (.68)	4.16 (.65)	3.94 (.78)
Intention to ask for bc screening	-	-	-	3.35 (1.33)	3.35 (1.29)	3.44 (1.22)	3.31 (1.40)	3.45 (1.41)
Diet								
No	46 (38%)	49 (37%)	39 (36%)	24 (41%)	30 (53%)	29 (39%)	27 (37%)	13 (45%)
Yes	76 (62%)	81 (62%)	69 (64%)	34 (59%)	27 (47%)	45 (61%)	47 (63%)	16 (55%)
BC Among Relatives								
No	117 (96%)	117 (90%)	89 (82%)	52 (90%)	48 (84%)	65 (88%)	67 (90%)	28 (97%)
Yes (Mother)	4 (3%)	8 (6%)	17 (16%)	5 (9%)	6 (11%)	7 (10%)	7 (10%)	1 (3%)
Do not know	1 (1%)	5 (4%)	2 (2%)	1 (1%)	3 (5%)	2 (2%)	-	-

For peer review only - http://bmjopen.bmj.com/site/about/guidelines.xhtml

Mammography								
No	100 (82%)	100 (77%)	72 (67%)	40 (69%)	38 (67%)	50 (68%)	50 (68%)	23 (79%)
Yes	22 (18%)	30 (23%)	36 (33%)	18 (31%)	19 (33%)	24 (32%)	24 (32%)	6 (21%)
Biopsy								
No	17 (77%)	27 (90%)	26 (72%)	57 (98%)	55 (97%)	67 (91%)	71 (96%)	29 (100%)
Yes	5 (23%)	3 (10%)	10 (28%)	1 (2%)	2 (3%)	7 (9%)	3 (4%)	-
Knowledge of BC								
Screening Program	76 (62%)	69 (53%)	64 (59%)	21 (36%)	23 (40%)	26 (35%)	27 (37%)	7 (24%)
No	46 (38%)	61 (47%)	44 (41%)	37 (64%)	34 (60%)	48 (65%)	47 (63%)	22 (76%)
Yes								
Knowledge of the age thresholds for BC Screening Program								
Do not know	16 (35%)	24 (39%)	13 (30%)	21 (36%)	32 (56%)	26 (35%)	27 (37%)	7 (24%)
Wrong	22 (48%)	28 (46%)	30 (68%)	29 (50%)	18 (32%)	34 (46%)	37 (50%)	18 (62%)
Correct	8 (17%)	9 (15%)	1 (2%)	8 (14%)	7 (12%)	14 (19%)	10 (13%)	14 (14%)
**POST-TEST VARIABLES**								
Intention to ask for BC screening	2.20 (1.05)	2.26 (1.06)	3.36 (1.33)	3.02 (1.61)	2.89 (1.48)	3.17 (1.48)	3 (1.54)	2.78 (1.49)
Results from Ancova[a] or Repeted Measures Ancova[b]	$F^a(2, 319) = 49.57$, $p < .0001$, $\eta^2_p = .24$<br><br>Promotion fit vs. Control Condition $t(319) = -8.80$, $p < .0001$, $r = .44$<br>Prevention Fit vs. Control Condition $t(319) = -8.80$, $p < .0001$, $r = .44$<br><br>Significant covariates<br>Fear of Breast Cancer: $F(1, 319) = 6.81$, $p = .010$, partial $\eta^2_p = .02$]<br>Age: $F(1, 319) = 26.20$, $p < .0001$, partial $\eta^2_p = .08$			Within subject comparison between pre- and post- intention: $F^b (1, 267.91) = 5.10$, p = .025, partial $\eta^2 = .02$<br>Between subject comparisons among groups: $F^b (4, 284) = .43$, p > .05<br><br>Significant covariates:<br>Fear of breast cancer: t(284) = 2.76, p = .006, B = .24, partial $\eta^2 = .03$ (95% Low CI = .07, 95% High CI = .42)<br>Age, t(284) = 6.26, p < .0001, B = .11, partial $\eta^2 = .12$ (95% Low CI = .08, 95% High CI = .15),<br>Risk perception, t(284) = 2.26, p = .024, B = .37, partial $\eta^2 = .02$ (95% Low CI = .05, 95% High CI = .70),				

Note: BC = Breast Cancer

For peer review only - http://bmjopen.bmj.com/site/about/guidelines.xhtml

Further analyses were conducted to evaluate whether the covariates might interact with the three experimental conditions in determining the intention to ask for breast cancer screening. Analyses revealed only one association among the three groups of women and the past diagnoses of breast cancer among first degree-relatives, χ^2 (2) = 12.98, p = .002. Women in the promotion fit condition had a lower number of breast cancer diagnoses among first-degree relatives than was expected (z = -1.96), while women in the control condition had a higher number than expected (z = 2.8). The subsequent ANCOVA did not find any significant interaction between past diagnosis of breast cancer among first-degree relatives and the experimental manipulations, therefore demonstrating that the regulatory fit genuinely influences the intention.

Phase 2

There was a general significant decrease of the intention from pre- to post-evaluation across groups, but no significant differences among them, indicating that the scores of the post-test intention among the five groups were in general the same. Among the covariates older women, greater fear of breast cancer and greater risk perception were associated with greater post-test intention compared to the opposite. Table 2 shows descriptive statistics and results from the analysis.

The intervention effect was not significant either when the two fit conditions and the two non-fit conditions were collapsed into two categories (i.e., comparison among fit condition vs. un-fit condition vs. control) as done in Phase 1, even though a general decrease in the post-intention across groups was found as before. Risk perception was tested as a moderator, but the analysis was not significant.

General discussion

The present research shows inconsistent results. Phase 1 confirmed the hypothesized effect of the intervention on the intention to seek mammography screening before the age of 45, with a reduction of the intention when a fit between the message frame and the individual's regulatory focus occurred. Longitudinal results from Phase 2 demonstrated that this effect was not significant over one month, although a general decrease of the intention across groups was observed. Even though further evidence is needed to confirm our results, it still seems that the 'just-feels-right' experience appears to be insufficient to convince non high-risk women under the age threshold to avoid systematic breast cancer screening in the long run.

Our results could genuinely reflect the fact that the regulatory fit is not sufficient to induce a long-term decrease in women's intentions or could be an artefact of the research itself. Phase 1 and Phase 2 applied two different ways to evoke a regulatory orientation. Phase 1 primed the individuals' regulatory orientation, whereas Phase 2 measured it with a questionnaire to overcome a limitation of Phase 1 and explore a different aspect of the theory. One could argue that the different ways to induce vs. measure the regulatory orientation could have influenced the persuasiveness of the message and so its effectiveness. However, researchers of regulatory orientation suggest that there is no difference between the two procedures,[39]. Therefore, we could exclude that the two methods have had a differential impact on post-test intention. Possible differences in the cultural milieu of Italian-speaking Swiss and Italian participants might make the population primed to receive or primed to ignore the intervention. However, to the best of our knowledge there is no study comparing different cultural environments in the propensity to be primed or not.

The relatively small sample size and the recruitment strategies could have influenced the power of the analyses, the sample composition and, ultimately, the significance of the results. However, there is no such concern in Phase 2 since the

effect due to the intervention was not significant either when the two fit conditions and the two non-fit conditions were collapsed into two categories.

Finally, a variable might have moderated the association between intervention and intention. As [48] demonstrated, individuals' consideration of future consequences of a particular behaviour influences the effectiveness of framing techniques in predicting risk perceptions, attitudes and behavioural intentions regarding health-related advertisements. In our research, the risk perception was tested as a moderator variable, but the analyses yielded no significant results.

Fear of breast cancer, age, and risk perception (only in Phase 2) were significantly related to women's intentions. The predicting role of age is not surprising because, approaching the age of 50, women are invited to undertake regular mammography screening in Ticino and in Italy. Risk perception and fear of breast cancer are the most sensitive variables. Breast cancer naturally evokes negative emotions,[27,49-51]. Moreover, the benefits of mammography screening often seem to be overestimated,[30,31]. Therefore, it is challenging to develop effective health messages promoting the adherence to breast cancer screening guidelines for young women based on factual information. As messages based on the principles of regulatory fit take the motivational orientations of recipients into account, they go beyond the effectiveness of purely providing information. Here, messages building on the theory of regulatory fit did not seem to offer a new way to overcome the 'emotional barrier' generated by the fear of breast cancer. However, Phase 2 demonstrated a general 'pedagogical effect' deriving from talking about the topic of breast cancer screening without evoking a boomerang effect (i.e. an increase of intention instead of a decrease).

The present research has several limitations. We experienced high dropout rates, especially in Phase 2. The high dropout rates may be related to the topic of breast

cancer itself or the fear associated with it. One could assume that women with a low level of fear of breast cancer may have decided not to take part in our research, and this may have created a selection bias that could affect the generalizability of the results. A second limitation concerns the fact that we measured the intention to ask for breast cancer screening, not the actual behaviour. Although according to many theories in the field of health promotion (e.g. Health Belief Model), the intention is a valid predictor of the actual behaviour, it would be beneficial if future research followed women until the moment they actually have a mammography.

In conclusion, it seems that by framing health messages that conform to a promotion or prevention focus, a decrease in the intention to ask for merely preventive opportunistic mammography screening is observed; but this takes place only immediately after message exposure. The influence decreases over time, and the messages lose their predictive effects after one month. This may be because breast cancer fear/opinions are very deeply ingrained in women and one/two messages cannot change that. Accordingly, possibly results from Phase 1 are valid, but repeated exposure to more than one regulatory fit message is needed to change viewpoints in the long term.

Even though our results only partially confirmed our hypothesis, there are substantial implications for future research. The results demonstrate that fear of breast cancer and risk perception are the main challenges to face in order to promote adherence to evidence-based recommendations on breast cancer screening. Public health researchers must investigate what factors may increase the effectiveness of health information. According to our evidence, future research may consider understanding how to reduce the impact of negative emotions rather than try to overcome their effect. For example, a research [52] found that humour in health messages reduces the anxiety associated with performing cancer screening. Humour

may be implemented in health messages aimed to promote evidence-based breast cancer screening recommendations. Reducing the number of unnecessary breast cancer screenings would thus allow the prevention of avoidable false positive and false negative diagnoses and unjustifiable mental and physical suffering for women. In the long term, this would also enable policy-makers and health professionals to allocate scarce resources for disease prevention, detection and treatment in a more effective way.

Funding Statement

This work was supported by the Swiss National Science Foundation [grant number FNS 100019-153131/1].

Competing interests statement

None declared.

Author contributions

SP and RL drafted the first version of the manuscript. All authors contributed to writing and critically revising it and approved its final version. PJS acquired funding. NHML, PJS and RL designed Phase 1 and prepared the materials. PJS, SP and RL designed Phase 2 and prepared the materials. SP and RL collected data for Phase 1. SP collected data for Phase 2. SP performed the analyses for Phase 1 and Phase 2.

Acknowledge

We are very grateful to Prof. John Hodgson for his thoughtful revisions.

Data Availability Statement

The data sets supporting the findings of Phase 1 and Phase 2 are available from the corresponding author, [SP], upon reasonable request.

References

[1] Torre, L.A., Islami, F., Siegel, R.L., Ward, E.M., Jemal, A., (2017). Global cancer in women: burden and trends. *Cancer Epidemiology, biomarkers & Prevention. CEBP Focus: Global Cancer in Women, 26(4),* 444-457.

[2] Altobelli, E., & Lattanzi, A. (2014). Breast cancer in European Union: An update of screening programs as of March 2014 (Review). *International Journal of Oncology, 45(5),* 1785–1792.

[3]Bucchi, L., Ravaioli, A., Baldacchini, F., Giuliani, O., Mancini, S., Vattiato, R., ... & Di Felice, E. (2019). Annual mammography at age 45–49 years and biennial mammography at age 50–69 years: comparing performance measures in an organised screening setting. *European radiology*, *29*(10), 5517-5527.

[4]Gøtzsche, P. C., & Jørgensen, K. J. (2013). Screening for breast cancer with mammography. *Cochrane Database of Systematic Reviews*, *6*, CD001877.

[5] van den Ende, C., Oordt-Speets, A. M., Vroling, H., & van Agt, H. M. (2017). Benefits and harms of breast cancer screening with mammography in women aged 40–49 years: A systematic review. *International Journal of Cancer*, *141*(7), 1295-1306.

[6] Armstrong, K., Moye, E., Williams, S., Berlin, J. A., & Reynolds, E. E. (2007). Screening mammography in women 40 to 49 years of age: a systematic review for the American College of Physicians. *Annals of Internal Medicine, 146,* 516–26.

[7]Barratt, A. L., Howard, K., Irwig, L., Salked, G., & Houssami, N. (2005). Model of outcomes of screening mammography: information to support informed choices. *British Medical Journal, 330,* 936–938.

[8]Gøtzsche, P. C., & Nielsen, M. (2006). Screening for breast cancer with mammography. *Cochrane Database of Systematic Review*, *4,* CD001877.

[9]Carter, S. M., Williams, J., Parker, L., Pickles, K., Jacklyn, G., Rychetnik, L., & Barratt, A. (2015). Screening for cervical, prostate, and breast cancer: interpreting the evidence. *American Journal of Preventive Medicine*, *49*(2), 274-285. [10]Mainiero, M. B., Lourenco, A., Mahoney, M. C., Newell, M. S., Bailey, L., Barke, L. D., ... & Jokich, P. M. (2016). ACR appropriateness criteria breast cancer screening. *Journal of the American College of Radiology*, 13(11), R45-R49.

[11]European Commission Initiative on Breast Cancer (ECIBC) Guideline (2018). European guidelines on breast cancer screening and diagnosis. Retrieved from: https://healthcare-quality.jrc.ec.europa.eu/european-breast-cancer-guidelines/screening-ages-and-frequencies#recs-group-40-44

[11]Oeffinger KC, Fontham ET, Etzioni R et al (2015) Breast cancer screening for women at average risk: 2015 guideline update from the American Cancer Society. *JAMA* 314:1599–1614

[13]Lauby-Secretan, B., Scoccianti, C., Loomis, D., Benbrahim-Tallaa, L., Bouvard, V., Bianchini, F., & Straif, K. (2015). Breast-cancer screening—viewpoint of the IARC Working Group. *New England journal of medicine*, 372(24), 2353-2358.

[14]Siu AL (2016) Screening for breast cancer: U.S. Preventive Services Task Force recommendation statement. *Annals Internal Medicine* 164:279–296

[15]Ahmad, F., Cameron, J. I., & Stewart, D. E. (2005). A tailored intervention to promote breast cancer screening among South Asian immigrant women. *Social science & medicine*, 60(3), 575-586.

[16]Azaiza, F., & Cohen, M. (2006). Health beliefs and rates of breast cancer screening among Arab women. *Journal of Women's Health*, 15(5), 520-530.

[17]Payán, D. D., Maggard-Gibbons, M., Flórez, K. R., Mejía, N., Hemmelgarn, M., Kanouse, D., ... & Lara, M. (2020). Taking Care of Yourself and Your Risk for Breast Cancer (CUIDARSE): A Randomized Controlled Trial of a Health Communication Intervention for Latinas. *Health Education & Behavior*, 1090198120920529.

[18]Woloshin, S., Schwartz, L. M., Black, W. C., & Kramer, B. S. (2012). Cancer screening campaigns--getting past uninformative persuasion. The New England journal of medicine, 367(18), 1677.

[19]Kregting, L. M., van Ravesteyn, N. T., Spijker, W., Dierks, T., Aitken, C. A., Geuzinge, H. A., & Korfage, I. J. (2020). Effects of a leaflet on breast cancer screening knowledge, explicit attitudes, and implicit associations. Patient Education and Counseling.

[20]Quaife, S. L., Ruparel, M., Beeken, R. J., McEwen, A., Isitt, J., Nolan, G., ... & Wardle, J. (2016). The Lung Screen Uptake Trial (LSUT): protocol for a randomised controlled demonstration lung cancer screening pilot testing a targeted invitation strategy for high risk and 'hard-to-reach'patients. BMC cancer, 16(1), 1-9.

[21]Roberto, A., Colombo, C., Candiani, G., Satolli, R., Giordano, L., Jaramillo, L., ... & Valenza, M. (2020). A dynamic web-based decision aid to improve informed choice in organised breast cancer screening. A pragmatic randomised trial in Italy. British Journal of Cancer, 1-8.

[22]Mathieu, E., Barratt, A., Davey, H. M., McGeechan, K., Howard, K., & Houssami, N. (2007). Informed choice in mammography screening: a randomized trial of a decision aid for 70-year-old women. Archives of internal medicine, 167(19), 2039-2046.

[23]Smith, J., Dodd, R. H., Hersch, J., Cvejic, E., McCaffery, K., & Jansen, J. (2020). Effect of different communication strategies about stopping cancer screening on

screening intention and cancer anxiety: a randomised online trial of older adults in Australia. BMJ Open, 10(6), e034061.

[24]Block, L. D., Jarlenski, M. P., Wu, A. W., & Bennett, W. L. (2013). Mammography use among women ages 40-49 after the 2009. US Preventive Services Task Force recommendation. *Journal of General Internal Medicine, 28(11),* 1447-1453.

[25]Kapp, J. M., Ryerson, A. B., Coughlin, S. S., & Thompson, T. D. (2009). Racial and ethnic differences in mammography use among US women younger than age 40. Breast Cancer Research and Treatment, 113(2), 327-337.

[26]Glaus, A., Fäh, B., Hornung, R., Senn, H., & Stiefel, F. (2004). Das Brustkrebs-Präventionsverhalten in der Schweiz: aus der Perspektive von Frauen aus drei Sprachregionen der Schweiz. [Breast cancer prevention behaviour: a perspective of women from three language regions of Switzerland]. Pflege, 17(6), 385-394.

[27]Labrie, N. H., Ludolph, R. A., & Schulz, P. J. (2020). Mammography perceptions and practices among women aged 30–49: The role of screening programme availability and cultural affiliation. Patient Education and Counseling, 103(2), 369-375.

[28]Klug, S. J., Hetzer, M., & Blettner, M. (2005). Screening for breast and cervical cancer in a large German city: participation, motivation and knowledge of risk factors. The European Journal of Public Health, 15(1), 70-77.

[29]Statistics Netherlands (2015). CBS: Ruim de helft van de vrouwen laat uitstrijkje maken. [More than half of all women get a pap-smear]. Retrieved from: http://www.cbs.nl/nl-NL/menu/themas/gezondheid-welzijn/publicaties/artikelen/archief/2015/ruim-de-helft-van-de-vrouwen-laat-uitstrijkje-maken.htm

[30]Chamot, E., & Perneger, T. V. (2001). Misconceptions about efficacy of mammography screening: a public health dilemma. Journal of Epidemiology & Community Health, 55, 799-803.

[31]Gigerenzer, G., Mata, J., & Frank, R. (2009). Public knowledge of benefits of breast and prostate cancer screening in Europe. Journal of the National Cancer Institute, 101(17), 1216-1220.

[32]Domenighetti, G., D'avanzo, B., Egger, M., Berrino, F., Perneger, T., Mosconi, P., & Zwahlen, M. (2003). Women's perception of the benefits of mammography screening: population-based survey in four countries. International journal of epidemiology, 32(5), 816-821.

[33]Parker, L., Carter, S., Williams, J., Pickles, K., & Barratt, A. (2017). Avoiding harm and supporting autonomy are under-prioritised in cancer-screening policies and practices. European Journal of Cancer, 85, 1-5.

[34]Willis, K. (2012). Choice, Trust and Risk-The Policy Context and Mammography Screening. In Mammography: recent advances (pp. 3-24). InTech Publishers, Croatia.

[35]Higgins, E. T. (2000). Making a good decision: value from fit. American Psychologist, 55(11), 1217-1227.

[36]Higgins, E. T. (1997). Beyond pleasure and pain. American Psychologist, 52(12), 1280-1300.

[37]Keller, P. A. (2006). Regulatory focus and efficacy of health messages. *Journal of Consumer Research, 33(1),* 109-114.

[38]Higgins, E. T., Friedman, R. S., Harlow, R. E., Idson, L. C., Ayduk, O. N., & Taylor, A. (2001). Achievement orientations from subjective histories of success: promotion pride versus prevention pride. *European Journal of Social Psychology, 31(1),* 3-23.

[39]Cesario, J., Higgins, E. T., & Scholer, A. A. (2008). Regulatory fit and persuasion: basic principles and remaining questions. *Social Personality Psychological Compass*, *2*, 444-63.

[40]Cesario, J., Grant, H., & Higgins, E. T. (2004). Regulatory fit and persuasion: transfer from' feeling right.' *Journal of Personality and Social Psychology, 86(3),* 388-404.

[41]Ludolph, R., & Schulz, P. J. (2015). Does regulatory fit lead to more effective health communication? A systematic review. Social science & medicine, 128, 142-150.

[42]Motyka, S., Grewal, D., Puccinelli, N. M., Roggeveen, A. L., Avnet, T., Daryanto, A., ... & Wetzels, M. (2014). Regulatory fit: A meta-analytic synthesis. Journal of Consumer Psychology, 24(3), 394-410.

[44]Uskul, A. K., Keller, J., & Oyserman, D. (2008). Regulatory fit and health behavior. Psychology and Health, 23(3), 327-346.

[45]Zhao, G., & Pechmann, C. (2007). The impact of regulatory focus on adolescents' response to antismoking advertising campaigns. Journal of Marketing Research, XLIV, 671-6

[44]Spiegel, S., Grant-Pillow, H., & Higgins, E. T. (2004). How regulatory fit enhances motivational strength during goal pursuit. European Journal of Social Psychology, 39, 39–54.

[46]Faul, F., Erdfelder, E., Lang, A. G., & Buchner, A. (2007). G*Power 3: A flexible statistical power analysis program for the social, behavioural, and biomedical sciences. *Behavior Research Methods*, 39, 175-191.

[47]Freitas, A. L., & Higgins, E. T. (2002). Enjoying goal-directed action: The role of regulatory fit. *Psychological science*, 13(1), 1-6.

[48] Kees, J. (2011). Advertising framing effects and consideration of future consequences. *The Journal of Consumer Affairs*, 45(1), 7-32.

[49]Consedine, N. S., Magai, C., Krivoshekova, Y. S., Ryzewicz, L., & Neugut, A. I. (2004). Fear, anxiety, worry, and breast cancer screening behavior: a critical review. *Cancer Epidemiology and Prevention Biomarkers, 13(4),* 501-510.

[50]Hay, J. L., McCaul, K. D., & Magnan, R. E. (2006). Does worry about breast cancer predict screening behaviors? A meta-analysis of the prospective evidence. *Preventive medicine, 42(6)*, 401-408.

[51]Nekhlyudov, L., Ross-Degnan, D., & Fletcher, S. W. (2003). Beliefs and expectation of women under 50 years old regarding screening mammography: a qualitative study. *Journal of General Internal Medicine, 18(3),* 182-9.

[52]Nabi, R. L., (2016). Laughting in the face of fear (of disease detection): using humor to promote cancer self-examination behavior. Health Communication, 31(7), 873-883.

Figure 1. Flowchart of the Study 1.

Figure 2. Flowchart of the Study 2.

Figure 1. Flowchart of the Study 1.

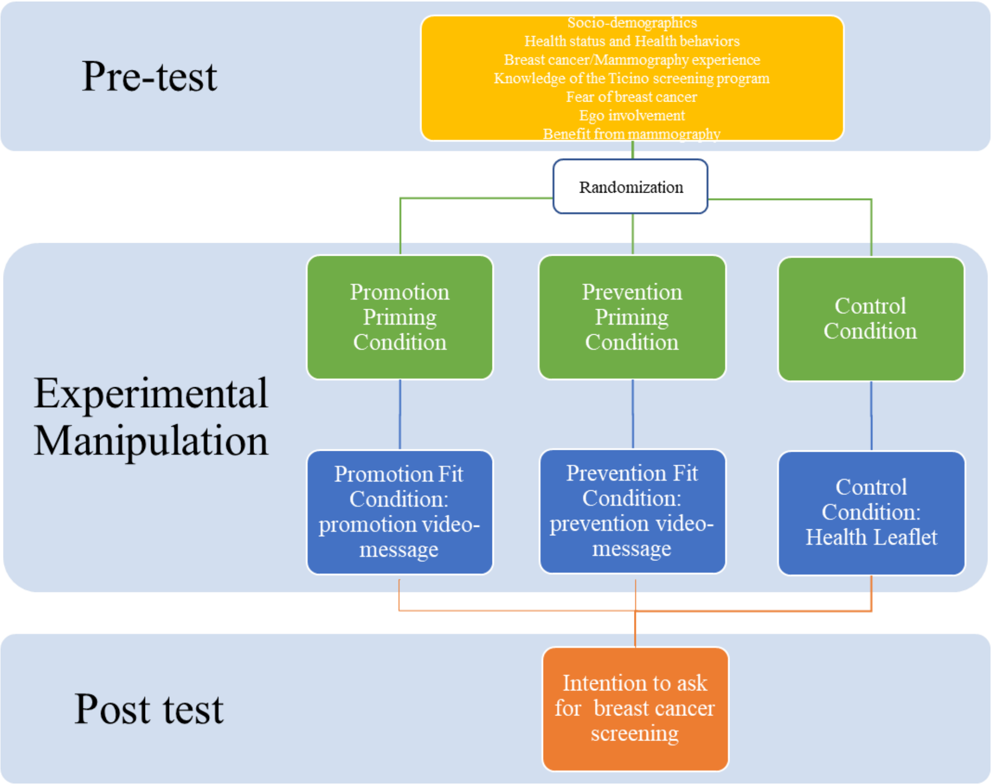

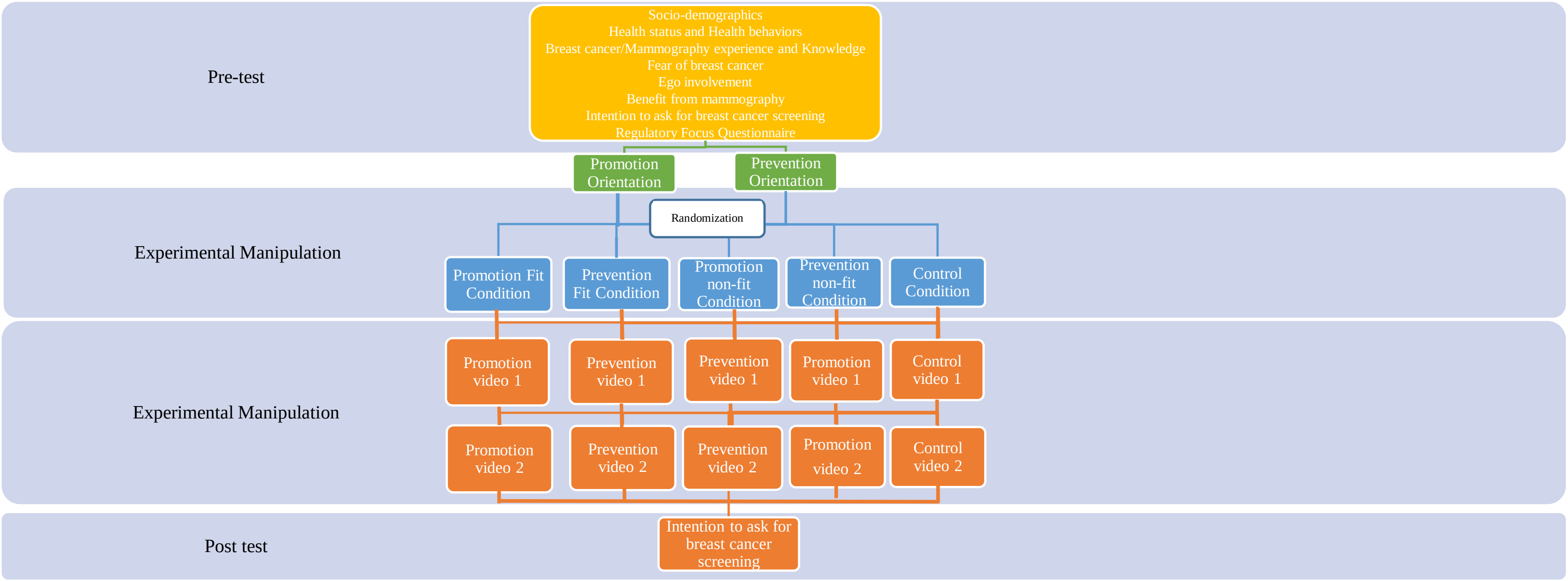
Pre-test
Socio-demographics
Health status and Health behaviors
Breast cancer/Mammography experience and Knowledge
Fear of breast cancer
Ego involvement
Benefit from mammography
Intention to ask for breast cancer screening
Regulatory Focus Questionnaire
Promotion Orientation
Prevention Orientation
Randomization
Experimental Manipulation
Promotion Fit Condition
Prevention Fit Condition
Promotion non-fit Condition
Prevention non-fit Condition
Control Condition
Experimental Manipulation
Promotion video 1
Prevention video 1
Prevention video 1
Promotion video 1
Control video 1
Promotion video 2
Prevention video 2
Prevention video 2
Promotion video 2
Control video 2
Post test
Intention to ask for breast cancer screening

SUPPLEMENTAL MATERIAL

An Application of the Theory of Regulatory Fit to Promote Adherence to Evidence-Based Breast Cancer Screening Recommendations Reducing Unnecessary Breast Cancer Mammography in Women Below Under 50: Experimental vs. Longitudinal Evidence

Serena Petrocchi, Ramona Ludolph, Nanon H. Labrie, Peter J. Schulz

Correspondence to:

Dr. Serena Petrocchi, Institute of Communication and Health, Università della Svizzera italiana, Via Buffi 13, 6900 Lugano, Switzerland.

E-mail: serena.petrocchi@usi.ch

Phone: 0041 (0) 58 666 4821

For peer review only

Study 1

Pre-test measures

Health Status and Healthy Lifestyle. Questions measured overall health status as perceived by the participants on a 5-point Likert scale and healthy lifestyle behaviours (i.e., diet, physical activity, smoking habits, alcohol consumption; see,[1].

Breast Cancer/Mammography Experience and Knowledge of the Ticino screening program. Participants replied to a set of questions on: past diagnosis of breast cancer among first-grade relatives,[2], if they had a mammography in the past, if doctor recommended the mammography, if they had a breast biopsy, if they know the breast cancer screening program in Ticino, and its age thresholds.

Fear of Breast Cancer. Four of the original 8 items of the Fear of Breast Cancer scale,[3,4] were administered. Items asked participants to rate their emotional reaction about breast cancer saying how much they agreed with the statements ‘When I think about breast cancer, I feel nervous (or: I get upset, I get jittery, I feel anxious)’. Participants replied on a 5-point Likert scale from 1 (strongly disagree) to 5 (strongly agree). Data from the present sample show that internal consistency was good, Cronbach's α = .88, rs > .73, as well as the factor structure, χ^2 (1) = 2.04, p = .15, CFI = .99, RMSEA = .05.

Ego-involvement. The Personal Involvement Inventory,[5] were administered measuring participants' involvement in breast cancer screening through affective and cognitive adjectives because previous research,[4]. The scale was administered as a 7-digit semantic differential (e.g., important/unimportant, relevant/irrelevant or worthless/valuable). The original item ‘of concern to me/of not concern to me’ was deleted based on results of a previous study,[4]. Data from the present sample show that internal consistency was good, Cronbach's α = .91, rs > .71, as well as and the factor structure, χ^2 (5) = 11.34, p = .04, CFI = .99, RMSEA = .06.

Perceived benefits of mammography screening. The perceived benefit of mammography screening was measured by four items,[6]: ‘Having a mammogram will help me find breast lumps

For peer review only

early'; 'If I find a lump early through a mammogram, my treatment for breast cancer may not be as bad'; 'Having a mammogram is the best way for me to find a very small breast lump'; 'Having a mammogram will decrease my chance of dying from breast cancer'. Participants replied on a 5-point Likert-scale from 1 (strongly disagree) to 5 (strongly agree). Data from the present sample show that internal consistency was modest, Cronbach's $\alpha = .75$, rs > .49 and the factor structure was good, χ^2 (1) = .51, p = .47, CFI = 1, RMSEA = .00.

Experimental manipulation

Regulatory Focus Priming Procedure. Prevention induced participants were asked to list two of their current obligations and then write down five actions they could take to avoid failure in fulfilling them,[7]. Promotion induced participants were asked to list two aspirations and write down five actions they could take to ensure their accomplishment,[7].

Video Messages. Participants in the promotion fit condition watched a video message emphasizing promotion concerns (i.e., they should adhere to evidence-based recommendations on mammography screening for safety and health protection reasons). Participants in the prevention fit condition watched a video emphasizing prevention concerns (i.e., they should not abstain from following the evidence-based recommendations on mammography screening to avoid negative/side effects). Participants in the control group did not receive any priming and read a general health leaflet. See Supplemental Table 1 for details of the voice-text of the two video messages and the control leaflet.

For peer review only

Supplemental Table 1. Voice-text of the video messages and control leaflet of the Study 1.

STUDY 1

Promotion video-message | **Prevention video-message**

The mammography screening is a method for the early detection of breast cancer. Using x-rays, mammograms can identify very small tumours generally longer before they are palpable. Mammograms is a method that is used early, often even without symptoms. In a screening program, experts recommend mammography from the age of 50. Here in Ticino, women aged 50 and over are invited to voluntarily undergo mammography every two years at one of the accredited Radiology Centres. For most women between the ages of 50 and 69, the benefits of screening are greater than the risks, but nevertheless it is important to be properly informed to make the best decision about mammography.

Promotion: **To protect their health, women under the age of 50, without a medical indication or family history of cancer, are excluded from the program.**

Prevention: **To avoid adverse effects on their health, women under the age of 50 without a medical indication or family history of cancer are excluded from the program.**

Now I would like to explain the scientific reasoning behind the recommendation to not undergo a mammogram without a medical reason. Anyway, in case of doubt or symptoms, I suggest to contact your doctor. So, you are asking why women under the age of 50 are excluded from mammography screening. Scientific research shows that for women between the ages of 50 and 69 mammography screening is the most effective method for the early detection of breast cancer and for reducing the mortality rate associated with it. In contrast, for young women between the ages of 30 and 49, the disadvantages and risks to health are greater than the benefits. This is mainly due to the fact that women before menopause have a denser breast tissue.

Promotion: **Given the reasons I have just presented, one should avoid undergoing a mammogram early to prevent negative consequences.**

Prevention: **Given the reasons I have just presented, to early undergo a mammogram can lead to negative consequences.**

For example, mammography could show anomalies that, after additional diagnostic tests, could be proved to be benign. This type of error is called a false positive. If for women aged between 50 and 69 this risk is minimal, for young women is higher due to the denser breast tissue. In addition, breast cancer could not be seen by mammograms because it is too small and therefore the exam may appear normal although cancer is present. This is a false-negative result. These risks always exist, but they are higher for young women. As all medical testing, waiting for the outcome of the mammogram can generate a state of anxiety and the procedure sometimes can be perceived as painful. Radiation exposure also have health consequences. Although the exposure is minimal, for women under the age of 50 the risk is higher than the benefits of mammography. Furthermore, screening could lead to over-treatment for tumours that are benign. Over diagnosis represents approximately 1-10% of diagnosed cancers. This would expose young women to the negative effects of anti-cancer therapies, without a real need. In the absence of scientific evidence of the effectiveness of mammographic screening for young women, the inclusion of young women in the program would entail additional costs for society. These financial resources could be used to prevent other diseases.

Promotion: **For these reasons, it is recommended that young women follow the indications for breast cancer screening.**

Prevention: **For these reasons, it is recommended that young women do not ignore the indications related to breast cancer screening.**

Control Leaflet

Healthy eating associated with an active lifestyle is a useful way for disease prevention. An adequate and balanced diet plan guarantees an optimal supply of nutrients to meet the needs of your body. A balanced diet also allows to receive substances that play a protective and/or preventive role against diseases.

This booklet - thought for people of all ages without any particular diseases - explains the scientific reasoning behind the recommendation to follow a healthy diet even in the absence of particular weight or health disorders. Anyway, in case of doubts or problems, we suggest to contact your doctor.

'Man is what he eats.'

We eat several times a day, for a lifetime. With a life expectancy around 80, this corresponds to approximately 85.000 meals. Considering that each meal lasts an average of thirty minutes, we spend at least five years at a table. Adding the meal preparation time, the count easily rises to ten years or more. Nutrition is therefore a topic that deserves special attention. Balanced meals and a healthy lifestyle give a fundamental contribution to our daily well-being and efficiency.

«Fast casual» instead of «fast food»

'Fast casual' means eating healthy and fast and it is not a contradiction. The 'snack culture' is an old idea. Those who want to have a quick meal, however, must pay attention to its composition: dairy products, fruit, vegetables, salads and whole meal products are the basis for a new, healthy 'fast casual' menu.

The right fats for every need

The choice of fats must be made according to the intended use. Fats and oils, if heated for a long time, change with chemical reactions that can create unwanted substances harmful to health. In order to avoid such reactions, it is advisable to cook with fats that mainly contain saturated fatty acids, which are more stable at high temperatures.

Eating healthy is easy

Food provides the body with both the necessary energy and nutrients that allow it to function properly. The diet must therefore provide a correct caloric intake and a sufficient amount of nutrients. No food is so complete that it contains everything the body needs. The basic rule is therefore to eat everything and in a varied way.

Food pyramid

The food pyramid facilitates the quantitative choice between the various food groups. It is a useful tool for all those who want to have a healthy diet. It provides clear indications on what to eat, how often, and in what quantity.

How do I interpret it?

A balanced diet requires the foods at the base of the pyramid be consumed in greater quantities. Climbing up to the vertex, the quantities of food consumed should be limited. Nothing is forbidden, every food finds its place in a balanced diet, but the recommended quantities will depend on its location in the pyramid.

Give food the importance that deserves, eating healthier. Your health and well-being will be better!

Note: grey rounded rectangles show the common parts of promotion and prevention video-messages; orange rounded rectangles show the promotion video-message specific parts (text in bold); blue rounded rectangles show the prevention video-message specific parts (text in bold); the green rounded rectangle shows the content of the control leaflet. The Videos created for Study 1 can be retrieved from https://youtu.be/mperSG5_9yQ and https://youtu.be/KnhRUnDoSV0. Both videos last 3:28 minutes. The videos created for Study 2 can be retrieved from https://youtu.be/btM3HrvYDlQ, https://youtu.be/BZPjFPUQuvw, https://youtu.be/-lXzGpcmzD4, https://youtu.be/jRi8Y-sZvSc. A translation of the Italian voice-over has been provided in this Table.

For peer review only

Post-test Measures

Intention to ask for breast cancer screening. Intention was measured by the question "I am evaluating the idea to have a mammography screening for breast cancer in the next 2-3 years",[8]. Two further questions were added: "I have the intention to have a mammography screening for breast cancer in the next 2-3 years" and "I will take an appointment for a mammography screening for breast cancer in the next 2-3 years". Participants replied on a 5-point scale from 1 (definitely yes) to 5 (definitely not); participants' scores ranged 1-5, $M = 2.61$, and $S.D. = 1.14$, with higher scores indicating greater intention. Data from the present sample show that internal consistency was good, Cronbach's $\alpha = .97$, $rs > .94$.

For peer review only

Study 2

Measures

Pre-test Measures

Pre-test covariates were measured as for study 1. Intention to ask for breast cancer screening was asked during the pre-test with the three items applied in Study 1.

Trait Regulatory Orientation. The Regulatory Focus Questionnaire,[9] was applied in the pre-test phase. The questions asked how frequently several specific events occur in the participant's life. Six questions capture the promotion focus, and the other five the prevention focus. Participants replied on a 5-point scale from 1 (never) to 5 (very often). The scores for promotion and prevention scales were calculated averaging the answers on given items after reverse score: data show good internal consistency for both promotion, $\alpha = .66$, rs > .33, and prevention, $\alpha = .74$, rs > .47. The individual's chronic orientation was calculated by subtracting promotion score to prevention score,[9].

Experimental manipulation

Video Messages. Six video-messages were developed for the present study:

- Two video-messages emphasising prevention concerns;
- Two video-messages emphasising promotion concerns;
- Two video-messages without any prompt to regulatory orientation.

Supplemental Table 2 shows the content of the voice-text of the six video-messages.

Post-test Measures

Intention to ask for breast cancer screening. As for Study 1. Participants' scores ranged 1-5, M = 2.99, and S.D. = 1.5, with higher scores indicating greater intention

Supplemental Table 2: voice-text of the video messages for Study 2.

	STUDY 2		
	Promotion video-messages	Prevention video-messages	Control video-messages
VIDEO 1	For the early detection of breast cancer, experts recommend mammography to women aged 50 and over. Mammography is the most effective medical examination for the early detection of breast cancer. It consists of an X-ray exposure that allows you to identify even very small tumors, before they are palpable or recognizable. Women over the age of 50 are invited to undergo a mammogram every 2 years at an accredited radiology center. For women between 50 and 69, the benefits of the exam outweigh the risks. And before the age of 50?		
VIDEO 1	**To respect their health, women under 50 are excluded from the breast cancer screening program.** (PROMOTION FOCUS)	**To avoid adverse health effects, women under 50 are excluded from the breast cancer screening program.** (PREVENTION FOCUS)	**Women under 50 are excluded from the breast cancer screening program.** (CONTROL GROUP)
VIDEO 1	It is a medical recommendation: before the age of 50, the risks of the examination are greater than the benefits.		
VIDEO 1	**In the absence of proven risk, to take care of your health, doctors advise to not undergo a mammogram before the age of 50.** (PROMOTION FOCUS)	**In the absence of proven risk, to avoid adverse effects for your health, doctors advise to not undergo a mammogram before the age of 50.** (PREVENTION FOCUS)	**In the absence of proven risk, doctors advise to not undergo a mammogram before the age of 50.** (CONTROL GROUP)
VIDEO 1	Mammography is a breast test that allows you to detect even many small tumours. Over 50 years, it is done every two years. Women under the age of 50 are excluded from the breast cancer screening program, except in case of genetic predisposition or family history of breast cancer. What are the reasons for this decision?		
VIDEO 1	**The observance of the age threshold determines a decreasing of the probability of false positives: breast anomalies that are benign form.** (PROMOTION FOCUS)	**The observance of the age threshold allows avoiding the negative consequences caused by false positives: breast abnormalities that are benign form.** (PREVENTION FOCUS)	**In young women, false positives are highly likely. False positive are breast anomalies that are benign form.** (CONTROL GROUP)
VIDEO 1	The risk of false positives in young women is higher, because the breast tissue is denser.		
VIDEO 1	**It is advised not to make mammograms before the age of 50 in order not to expose themselves to anti-cancer treatments not recommended as they are often directed to benign anomalies.** (PROMOTION FOCUS)	**It is advisable not to make mammograms before the age of 50 to avoid exposure to non-recommended anti-cancer treatments as they are often directed to benign anomalies.** (PREVENTION FOCUS)	**The breast cancer screening could lead to an exposition of non-recommended anti-cancer treatments, as they are often directed to benign anomalies.** (CONTROL GROUP)
VIDEO 1	In fact, breast cancer is much rarer in women under the age of 50.		
VIDEO 1	**Excluding younger women from screening allows them to be protected from unnecessary radiation exposure. Furthermore, this choice promotes psychological well-being against stress and anxiety. For these reasons, mammographic screening involves only women over 50 years. If you are under 50 and want to take care of your health, we recommend that you respect the age threshold.** (PROMOTION FOCUS)	**Excluding younger women from screening allows you to avoid unnecessary radiation exposure. Furthermore, this choice avoids psychological discomforts such as stress and anxiety. For these reasons, mammographic screening involves only women over 50 years. If you are under 50 years old and want to avoid negative consequences for your health, we recommend that you respect the age threshold.** (PREVENTION FOCUS)	**Radiation exposure poses health risks. In the case of mammography, the exposure is minimal, but for women under the age of 50 the risk is higher than the benefits. Furthermore, as any other medical procedure, waiting for the outcome can generate anxiety and stress. For these reasons, mammographic screening involves only women over 50 years. If you are under 50 years old, we recommend that you respect the age threshold.** (CONTROL GROUP)
VIDEO 1	A conscious prevention, it's worthit!		

Note: grey rounded rectangles show the common parts of the video-messages; the orange rounded rectangles show the promotion video-message specific parts (text in bold); the blue rounded rectangles show the prevention video-message specific parts (text in bold); the green rounded rectangle shows the content of the control leaflet.

References

[1]Shim, M., Kelly, B., & Hornik, R. (2006). Cancer information scanning and seeking behavior is associated with knowledge, lifestyle choices, and screening. *Journal of Health Communication, 11(2),* 157–172.

[2]Daley, M. B., Lerman, C. L., Ross, E., Schwartz, M. D., Sands, C. B., & Masny, A. (1996). Gail model breast cancer risk components are poor predictors of risk perception and screening behaviour. *Breast Cancer Research and Treatment, 41,* 59–70.

[3]Champion, V. L., Skinner, C. S., Menon, U., Rawl, S., Giesler, R. B., Monahan, P., & Daggy, J. (2004). A breast cancer fear scale: Psychometric development. *Journal of Health Psychology, 9(6),* 753-762.

[4] Labrie, N. H., Ludolph, R. A., & Schulz, P. J. (2020). Mammography perceptions and practices among women aged 30–49: The role of screening programme availability and cultural affiliation. Patient Education and Counseling, 103(2), 369-375.

[5]Zaichkowsky, J. L. (1994). The personal involvement inventory: Reduction, revision, and application to advertising. Journal of Advertising, 23 (4), 59-70.

[6]Champion, V. L., Mohahan, P. O., Springston, J. K., Russell, K, Zollinger, T. W., Saywell, R. M. Jr., & Maraj, M. (2008). Measuring mammography and breast cancer beliefs in African American women. *Journal of Health Psychology, 13(6),* 827-837.

[7] Freitas, A. L., & Higgins, E. T. (2002). Enjoying goal-directed action: The role of regulatory fit. *Psychological science*, 13(1), 1-6.

[8]Hersch J., Barratt A., Jansen J., Irwig L., McGeechan K., Jacklyn G., & McCaffery K. (2015). Use of a decision aid including information on over detection to support informed choice about breast cancer screening: a randomized controlled trial. *The Lancet, 385(9978),* 1642-1652.

[9] Higgins, E. T., Friedman, R. S., Harlow, R. E., Idson, L. C., Ayduk, O. N., & Taylor, A. (2001). Achievement orientations from subjective histories of success: promotion pride versus prevention pride. *European Journal of Social Psychology, 31(1),* 3-23.

For peer review only

STROBE 2007 (v4) Statement—Checklist of items that should be included in reports of *cross-sectional studies*

Section/Topic	Item #	Recommendation	Reported on page #
**Title and abstract**	1	(*a*) Indicate the study's design with a commonly used term in the title or the abstract	#1; #2
		(*b*) Provide in the abstract an informative and balanced summary of what was done and what was found	#2
**Introduction**			
Background/rationale	2	Explain the scientific background and rationale for the investigation being reported	#4-7
Objectives	3	State specific objectives, including any prespecified hypotheses	#6-7
**Methods**			
Study design	4	Present key elements of study design early in the paper	#7; #11
Setting	5	Describe the setting, locations, and relevant dates, including periods of recruitment, exposure, follow-up, and data collection	#8; #11
Participants	6	(*a*) Give the eligibility criteria, and the sources and methods of selection of participants	#7-8; #10
Variables	7	Clearly define all outcomes, exposures, predictors, potential confounders, and effect modifiers. Give diagnostic criteria, if applicable	#8; #12
Data sources/ measurement	8*	For each variable of interest, give sources of data and details of methods of assessment (measurement). Describe comparability of assessment methods if there is more than one group	#8; #11
Bias	9	Describe any efforts to address potential sources of bias	#8-10; #10-12
Study size	10	Explain how the study size was arrived at	#7; #11
Quantitative variables	11	Explain how quantitative variables were handled in the analyses. If applicable, describe which groupings were chosen and why	#8-9; #12
Statistical methods	12	(*a*) Describe all statistical methods, including those used to control for confounding	#9; #12
		(*b*) Describe any methods used to examine subgroups and interactions	#9-11; #12
		(*c*) Explain how missing data were addressed	#9; #12
		(*d*) If applicable, describe analytical methods taking account of sampling strategy	Not applicable
		(*e*) Describe any sensitivity analyses	Not applicable
**Results**			

For peer review only

Participants	13*	(a) Report numbers of individuals at each stage of study—eg numbers potentially eligible, examined for eligibility, confirmed eligible, included in the study, completing follow-up, and analysed	#7; #10-11
		(b) Give reasons for non-participation at each stage	#7; #10-11
		(c) Consider use of a flow diagram	#8
Descriptive data	14*	(a) Give characteristics of study participants (eg demographic, clinical, social) and information on exposures and potential confounders	#7; #12
		(b) Indicate number of participants with missing data for each variable of interest	#9; #12
Outcome data	15*	Report numbers of outcome events or summary measures	#9-12
Main results	16	(*a*) Give unadjusted estimates and, if applicable, confounder-adjusted estimates and their precision (eg, 95% confidence interval). Make clear which confounders were adjusted for and why they were included	#9-12
		(*b*) Report category boundaries when continuous variables were categorized	Not applicable
		(*c*) If relevant, consider translating estimates of relative risk into absolute risk for a meaningful time period	Not applicable
Other analyses	17	Report other analyses done—eg analyses of subgroups and interactions, and sensitivity analyses	Not applicable
**Discussion**			
Key results	18	Summarise key results with reference to study objectives	#12-16
Limitations	19	Discuss limitations of the study, taking into account sources of potential bias or imprecision. Discuss both direction and magnitude of any potential bias	#15
Interpretation	20	Give a cautious overall interpretation of results considering objectives, limitations, multiplicity of analyses, results from similar studies, and other relevant evidence	#12-16
Generalisability	21	Discuss the generalisability (external validity) of the study results	#12-16
**Other information**			
Funding	22	Give the source of funding and the role of the funders for the present study and, if applicable, for the original study on which the present article is based	#16

*Give information separately for cases and controls in case-control studies and, if applicable, for exposed and unexposed groups in cohort and cross-sectional studies.

Note: An Explanation and Elaboration article discusses each checklist item and gives methodological background and published examples of transparent reporting. The STROBE checklist is best used in conjunction with this article (freely available on the Web sites of PLoS Medicine at http://www.plosmedicine.org/, Annals of Internal Medicine at http://www.annals.org/, and Epidemiology at http://www.epidem.com/). Information on the STROBE Initiative is available at www.strobe-statement.org.

For peer review only
